# Range-Wide Phylogeography and Ecological Niche Modeling Provide Insights into the Evolutionary History of the Mongolian Racerunner (*Eremias argus*) in Northeast Asia

**DOI:** 10.3390/ani14071124

**Published:** 2024-04-07

**Authors:** Lili Tian, Rui Xu, Dali Chen, Natalia B. Ananjeva, Rafe M. Brown, Mi-Sook Min, Bo Cai, Byambasuren Mijidsuren, Bin Zhang, Xianguang Guo

**Affiliations:** 1 Chengdu Institute of Biology, Chinese Academy of Sciences, Chengdu 610223, China; tianll@cib.ac.cn (L.T.); xurui@cib.ac.cn (R.X.); caibo@cib.ac.cn (B.C.); 2 University of Chinese Academy of Sciences, Beijing 100049, China; 3 Department of Pathogenic Biology, West China School of Basic Medical Sciences and Forensic Medicine, Sichuan University, Chengdu 610041, China; chendali@scu.edu.cn; 4 Zoological Institute, Russian Academy of Sciences, St. Petersburg 199034, Russia; natalia.ananjeva@zin.ru; 5 Biodiversity Institute, Department of Ecology and Evolutionary Biology, University of Kansas, Lawrence, KS 66045, USA; rafe@ku.edu; 6 Conservation Genome Resource Bank for Korean Wildlife, Research Institute for Veterinary Science, College of Veterinary Medicine, Seoul National University, Seoul 151-742, Republic of Korea; minbio@yahoo.co.kr; 7 Plant Protection Research Institute, Mongolian University of Life Sciences, Ulaanbaatar 210153, Mongolia; byambasuren.m@muls.edu.mn; 8 College of Life Sciences and Technology, Inner Mongolia Normal University, Hohhot 010022, China; zhangbin@imnu.edu.cn

**Keywords:** Bayesian phylogeographic diffusion, ecological niche modeling, landscape, Last Glacial Maximum, Northeast Asia, population dynamics, racerunner lizard

## Abstract

**Simple Summary:**

The Mongolian racerunner (*Eremias argus*) is a genetically diverse species with a wide distribution across Northeast Asia. The present study is the first to assess the genetic variation in *E. argus* across its entire geographic range using newly generated mtDNA cyt *b* and previously published data. We show the complex genetic pattern of this species, where most genetic divergence is not associated with geographic regions. This pattern is the result of multiple influences–robust dispersal ability resulting from ecological features, anthropogenic influence of translocation, and past climate change. Geographic distance has primarily shaped the genetic structure of the species, as indicated by a strong pattern of isolation by distance across all populations. The population growth of *E. argus* from the Last Interglacial to the Last Glacial Maximum is likely a consequence of an increase in favorable habitats with mild climates following the development of monsoons in East Asia since the mid-Late Pleistocene. Overall, the population genetic structure of *E. argus* was likely driven by a combination of climatic and geographic changes rather than direct ice sheet coverage. Our results highlight the importance of combining genetic approaches with environmental data when assessing the impact of Pleistocene climatic oscillations.

**Abstract:**

The Mongolian racerunner, *Eremias argus*, is a small lizard endemic to Northeast Asia that can serve as an excellent model for investigating how geography and past climate change have jointly influenced the evolution of biodiversity in this region. To elucidate the processes underlying its diversification and demography, we reconstructed the range-wide phylogeographic pattern and evolutionary trajectory, using phylogenetic, population genetic, landscape genetic, Bayesian phylogeographic reconstruction and ecological niche modeling approaches. Phylogenetic analyses of the mtDNA cyt *b* gene revealed eight lineages that were unbounded by geographic region. The genetic structure of *E. argus* was mainly determined by geographic distance. Divergence dating indicated that *E. argus* and *E. brenchleyi* diverged during the Mid-Pliocene Warm Period. *E. argus* was estimated to have coalesced at~0.4351 Ma (Marine Isotope Stage 19). Bayesian phylogeographic diffusion analysis revealed out-of-Inner Mongolia and rapid colonization events from the end of the Last Interglacial to the Last Glacial Maximum, which is consistent with the expanded suitable range of the Last Glacial Maximum. Pre-Last Glacial Maximum growth of population is presented for most lineages of *E. argus.* The Glacial Maximum contraction model and the previous multiple glacial refugia hypotheses are rejected. This may be due to an increase in the amount of climatically favorable habitats in Northeast Asia. Furthermore, *E. argus barbouri* most likely represents an invalid taxon. The present study is the first to report a range-wide phylogeography of reptiles over such a large region in Northeast Asia. Our results make a significant contribution towards understanding the biogeography of the entire Northeast Asia.

## 1. Introduction

Pleistocene glacial-interglacial cycles (2.6–0.012 Ma), which caused the major environmental changes, have played an important role in shaping the genetic diversity of species [1,2,3]. The Last Glacial Maximum (LGM; 0.026–0.019 Ma) was a major climatic event characterized by low temperatures and arid climates [4]. Due to the low temperatures caused by ice cover and the unsuitable arid climates during the LGM, the distribution ranges of temperate-adapted taxa are generally thought to have contracted [3,5]. However, for the cold-adapted taxa of the Northern Hemisphere or biota from non-glaciated regions, the Glacial Maximum (GM) contraction model may not be universally applicable [6,7]. For example, *Plethodon serratus* has been shown to fit a model of GM expansion [8], which is contrary to the common assumption of contraction into refugia during the LGM. Recently, another good case for testing the responses of cold-adapted and warm-adapted ectotherms in the Northern Hemisphere has been documented in European vipers [9]. Thus, the effects of the Pleistocene glacial cycles on the biotic elements are expected to vary between regions and differ between species, depending largely on the natural history and adaptation (e.g., environmental tolerance and dispersal ability) of organisms (e.g., [10,11,12,13]).

The non-static definition of Northeast Asia (NEA, [14]) includes the Mongolian plateau, northern China, the Japanese archipelago, the Korean Peninsula and the mountainous regions of the Russian Far East, all of which fall within a latitudinal range similar to that of central and southern Europe [15,16]. The NEA is characterized by a wet monsoon region in the east and a mid-latitude, westerly controlled dry inland region in the west in summer, where the ecosystem and environment are vulnerable to rainfall variability [17]. In addition to the rainy season, East Asian summers are also characterized by high temperatures. Temperature has pervasive effects on behavior, physiology, dispersal and migration due to its significant influence on the structure and function of biological systems [18]. Owing to the mild climate of the NEA based on monsoons and relatively high temperatures, and the biotic zones of Asia being at higher northern latitudes than on other continents, glacial advances in this region were not as extensive as in Europe and North America where lineages currently inhabiting formerly glaciated areas were pushed into southern glacial refugia [19,20,21] and subsequently expanded into modern regions after the LGM [2,22].

The Mongolian racerunner, *Eremais argus*, is a small oviparous and heliothermic lizard of the NEA, with a distribution ranging from the coast to the desert in China, Korea, North Korea, Mongolia and Russia, ranging from the arid zone to the humid zones [23,24,25,26]. Except for the population in Korea, which is listed as an endangered species [27,28], and that in North Korea, which has rather limited records, the populations of *E. argus* in the other three countries are relatively abundant, thus providing an excellent system for evaluating the phylogeographic pattern and investigating the effects of past climatic changes on the demographic history of the terrestrial lizard in the NEA. In addition, *E. argus* shows considerable intra-specific variation, as two subspecies have been proposed based on morphological characters and putative vicariant events [29]: the nominate form *E. argus argus* Peters, 1869 (with an eye-like dorsal pattern and more than 50 rows of dorsal scales on the midbody) and *E. argus barbouri* Schmidt, 1925 (with a striped dorsal pattern and fewer than 50 scales on the midbody). However, the details of the evolutionary history and population structure have not been rigorously investigated due to the small number of sampling localities in previous studies [17,28,30,31,32]. There are still significant geographic sampling gaps for *E. argus* in Mongolia and Russia, and even in northern China. The present study is therefore the first to assess the genetic variation in *E. argus* across its entire geographic range.

This study is motivated by the goals of obtaining a more detailed and complete picture of the genetic structure of the population, and of tracing the population history of *E. argus* under conditions of widespread and sufficient sampling, as well as sophisticated analyses based on phylogeography, landscape genetics and population dynamics. We combined analyses of divergence time, ENM and Bayesian phylogeographic diffusion. Specifically, we aimed to (i) depict the phylogeographic structure and timing of genetic diversification within the Mongolian racerunner; (ii) reconstruct colonization routes; (iii) explore the changing distribution during past typical periods and predict the future suitable ranges; (iv) test GM expansion versus GM contraction models for *E. argus*; (v) clarify the effect of variability in landscape features on the genetic structure of *E. argus*; and (vi) test the two-subspecies hypothesis and assess the taxonomic status within *E. argus*.

## 2. Materials and Methods

### 2.1. Sample Collection

**Figure 1:**
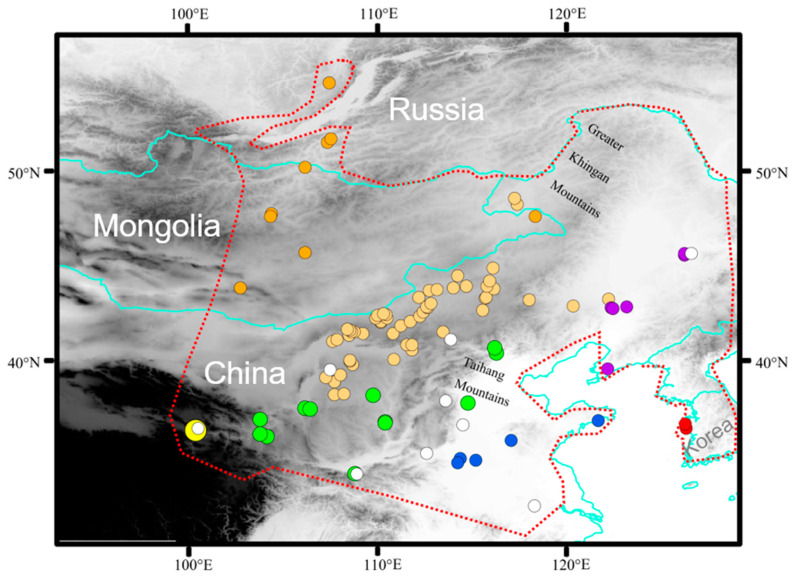
Geographic sampling of *Eremias argus*. Sampling locations are plotted on a map of the study areas and colored according to seven regions: dark orange indicates northern region; light orange indicates central region; green indicates southern region; yellow indicates western region; red indicates eastern region; blue indicates southwestern region; purple indicates northeastern region. White solid circles indicate samples from Zhao et al. [32]. Red dashed lines indicate the range of *E. argus*.

A total of 508 individuals of *E. argus* were collected during 2007–2021 from 96 sites throughout its known distribution range, including China, Mongolia, Russia and Korea. Liver samples and voucher specimens were fixed in 95% ethanol and deposited in the Chengdu Institute of Biology (CIB), Chinese Academy of Sciences. All animal procedures were approved by the Animal Care and Use Committee of CIB (approval number: CIB-20121220A). A total of 106 specimens from 9 localities were used in the study by Zhao et al. [32]. Specific information on all specimens is provided in Table S1, and all sampling locations of 614 specimens are shown in Figure 1.

As the range of *E. argus* and the Mongolian toad (*Strauchbufo raddei*) overlap in most of the modern areas, we categorized all sampling sites a priori into seven geographic regions (Northern region: Mongolia and Russia; Southwestern region: Qinghai Province; Northeast region: Liaoning and Heilongjiang provinces; Central region: Inner Mongolia; Southern region: the Ningxia, Shanxi, Shaanxi, Hebei, Gansu provinces and Beijing Municipality; Southeastern region: Shandong, Anhui and Henan provinces; Eastern Region: Korean Peninsula) and five landscapes (canyon, plateau, river basin, steppe, and lowland forest) [33,34].

### 2.2. DNA Extraction, Amplification and Sequence Analysis

Genomic DNA was extracted from ethanol preserved liver or tail tissue samples using the HiPure Universal DNA Kit (Magen Biotech, Guangzhou, China). To incorporate the data published by Zhao et al. [32], we amplified the mitochondrial DNA cytochrome *b* (cyt *b*) gene sequence in this study. The complete mtDNA cyt *b* gene sequence was amplified using the primers L14919 and H16064 from Burbrink et al. [35]. Detailed amplification and sequencing methods were adapted from Liu et al. [36]. Negative controls were included in all amplifications. All PCR products were commercially purified, and double strand sequenced. Sequences are deposited in GenBank under accession numbers OR019119–OR019626 (see Table S1). Three outgroup sequences of *Eremias* lizards (*E. brenchleyi*, *E. multiocellata*, and *E. velox*) were obtained from GenBank (see Table S3). The generated sequences were aligned using Clustal X v2.0 [37]. No indels were found in the cyt *b* matrices. General characterizations of DNA variation and haplotypes were generated in DnaSP v6.12.03 [38].

### 2.3. Phylogenetic Analysis

We used Bayesian inference (BI) and maximum likelihood (ML) to reconstruct phylogenetic relationships among cyt *b* haplotypes. We also used PartitionFinder v2.1.1 [39] to select the best partitioning strategy and the optimal nucleotide substitution model for the dataset using the Bayesian Information Criterion (BIC, see Table S2). MrBayes v3.2.6 [40] was used for partitioned Bayesian phylogenetic analyses, with four independent runs for twenty million generations and sampling every 5000 generations. A 50% majority-rule consensus tree and the posterior probability (PP) of clades were assessed by combining the sampled trees from the two independent runs after a 25% burn-in period. IQ-TREE v1.6.7 [41] was used to construct the ML tree. We used an ultrafast bootstrap approximation approach with 5000 bootstraps. FigTree v1.4.4 [42] was used for tree visualization, and Microsoft PowerPoint was used for tree editing.

### 2.4. Bayesian Test of Topology Hypothesis

*Eremias barbouri* was first described in 1925 by Karl Patterson Schmidt on the basis of a male racerunner lizard (holotype, AMNH 24045) collected by Clifford Hillhouse Pope from Mai Tai Chao, northern Shanxi Province, China [43]. This species was named after Dr. Thomas Barbour of the Museum of Comparative Zoology. In 1935, Clifford Hillhouse Pope placed Mai Tai Chao in Suiyuan Province, a region that is now part of the Inner Mongolia Autonomous Region, China [44]. Later, in 1935, Yaichirô Okada treated *E. barbouri* as a subspecies of *E. argus*, namely *E. argus barbouri* [45]. Since then, the idea that there are two subspecies of *E. argus* has become a popular one [24,25,26,29]. Here we tested the taxonomic status within *E. argus*—Are populations of *E. a. barbouri* and nominate subspecies monophyletic? Bayes factors (BF, [46]) were used to compare the unconstrained Bayesian tree topology with the Bayesian tree with ‘hard’ constraints. We constrained the topology for the putative groupings above and performed a stepping-stone run of 10 million generations (50 steps with convergence being reached at each step), from which we obtained a mean marginal likelihood estimate. The marginal likelihood estimate from the constrained topology was compared using BF with that from the unconstrained MrBayes run of the same length. The stepping=stone function [47] implemented in MrBayes v3.2.2 [40] provides an improved marginal estimation over the harmonic mean estimation.

### 2.5. Divergence Times Estimation

We used a relaxed lognormal clock approach to estimate the divergence time of lineages, as implemented in BEAST v1.8.4 [48]. Owing to limited reliable fossil evidence, we used secondary calibrations of robust divergence time calculations, adopting a two-step approach similar to that used by Liu et al. [10]. First, we retrieved the cyt *b* and 12S rRNA sequences from GenBank corresponding to 136 species in the family Lacertidae and three outgroup species representing Amphisbaenidae, Bipedidae and Gerrhosauridae (see Table S3) to introduce the calibration points. We randomly selected two representatives from the two most distant subclades of *E. argus* for the BEAST analyses (Figure S1). We then combined the dataset to obtain the most recent common ancestor (MRCA) of *E. argus*. Following previous studies [10,49,50,51], we set nine calibration points with a normal distribution of probability densities. In the second step, all *E. argus* sequences were used to estimate the time of divergence of the internal lineages with the above age estimate for the MRCA of *E. argus* (0.51 ± 0.2 Ma). Models, priors and parameters are given in Table S2.

### 2.6. Phylogeographic Diffusion in Continuous Space

Based on the current and predicted suitable ranges of *E. argus*, there are no obvious potential barriers that can be recorded to define discrete areas, so it is arbitrary to divide the species range into discrete operational areas a priori. We therefore inferred the spatial diffusion of genealogies using a continuous Bayesian phylogeographic method which models the geographic spread of genealogies across space with continuous diffusion processes conditional on sampling location, defined as bivariate traits representing latitude and longitude for each accession [52]. We first selected the best-fitting diffusion model by performing marginal likelihood estimation using generalized stepping-stone sampling on three available relaxed random walk (RRW) models, as well as the time-homogeneous Brownian motion process. Bayesian phylogeographic diffusion analysis was performed using BEAST v1.10.4 [53]. The Markov chain Monte Carlo (MCMC) was run for 50 million generations with trees and parameters sampled every 3000 generations. The convergence of the MCMC chains was checked using Tracer v1.7.1 [54] to ensure adequate mixing and convergence. Finally, the sampled trees were annotated using TreeAnnotator v2.6.2 and the results of phylogeographic reconstruction were produced in SpreaD3 [55].

### 2.7. Population Genetic Analysis

DnaSP v6.12.03 [38] was used to calculate the number of haplotypes (NH), haplotype diversity (HD), nucleotide diversities (ND) and mean number of base pair differences (MNPD) for two clades, seven subclades (excluding the single-haplotype clade, which referred to two sequences) and seven regions. PopART v1.7 [56] was used to generate the median-joining network (MJN) of all individuals and the results were compared with the resulting phylogenetic tree to visualize relationships between haplotypes within subclades. To assess whether genetic differentiation occurs across changing landscapes [57,58,59,60], we used five landscapes to implement hierarchical analysis of molecular variance (AMOVA) based on mitochondrial haplotype frequencies in Arlequin v3.5 [61]. We used the similar division of landscapes (Steppe: Inner Mongolia, Beijing, Shanxi, Hebei, Mongolia and Russia; River: Heilongjiang and Liaoning provinces; Canyon: Shaanxi and Gansu provinces; Lowland Forest: Shandong, Henan and Anhui provinces and Korea; Plateau: Gonghe County, Qinghai Province.) in Othman et al. [33]. Variation was calculated between groups, between populations within groups and within populations.

### 2.8. Testing Correlations between Genetic, Environmental, and Geographic Distances

To estimate an isolation-by-distance (IBD) matrix, we calculated pairwise Euclidean distances (in kilometers) using the Geographic Distance Matrix Generator v1.2.3 [62], and the genetic distance (*F*st/(1−*F*st)) using Arelquin v3.5. Mantel tests for genetic distance and geographic distance, and partial Mantel tests for genetic and geographic distance with control of the indicator (geographic distance and barriers, respectively) were implemented in IBD v1.52 [63]. To estimate a matrix of isolation-by-environment (IBE), nine climate factors (see Table S4) were used to calculate the environmental distance using the R vegan package [64]. We excluded those variables with Pearson correlation coefficients r > 0.8 based on pairwise comparison of raster files in SDMtoolbox v2.0 [65]. Mantel tests for genetic distance and environmental distance, and partial Mantel tests for genetic and environmental distance controlling for the indicator (geographic distance and environmental distance, respectively) were calculated in ZT v1.1 [66]. To estimate a matrix of isolation by resistance (IBR), we developed a present-day ENM of for the species in MAXENT v3.4.4 [67], and generated a friction layer, where grid cells with higher suitability scores were assigned lower friction values. We estimated circuit distances using Circuitscape v3.5.8 [68,69]. Finally, the Mantel test for genetic distance and resistance was calculated in ZT.

### 2.9. Inference of Demographic Histories

We performed demographic analyses for the resulting genetic structure of the species (including two clades and seven subclades, see Results). We calculated Fu’s *Fs* [70], Tajima’s *D* [71] statistics, with significance tested using 10,000 bootstrap replicates with DnaSP v6.12.03 [38]. Mismatch distributions (MD) of pairwise nucleotide differences and the Ramos-Onsins and Rozas [72] *R*_2_ statistic were also calculated using Arlequin v3.5 [61], with 10,000 coalescent simulations. Harpening’s Raggedness Index (*Rg*) and Sum of Squares Deviation (*SSD*) were calculated to test the goodness of fit between the observed and expected distributions. Finally, Bayesian skyline plots (BSP) were implemented in BEAST v1.8.4 [48] to estimate the changes in effective population size on an evolutionary time scale. We used a strict clock to construct BSPs for each subclade (see Table S2). We used a mean substitution rate of 6.23%/site/million years, with a 95% HPD of 3.2–8.97%/site/million years (derived from the divergence dating described above). Plots for each analysis were visualized using Tracer v1.7.1 [54].

### 2.10. Ecological Niche Modeling

We conducted ENM to predict a suitable habitat for *E. argus* during the Mid-Pliocene Warm Period and the Quaternary climate fluctuations. We obtained climate data at six epochs, including the Current (1979–2013), MPWP (3.205 Ma) and MIS 19 (0.787 Ma) from the PaleoClim database (http://www.paleoclim.org (accessed on 28 December 2023); [73]), Present (1960–1990), Last Glacial Maximum (LGM, 0.026–0.019 Ma), Last Interglacial (LIG, 0.14–0.12 Ma) and future (2070s) from the WorldClim 1.4 (http://www.worldclim.org (accessed on 28 December 2023); [74]). MIROC6 under the Shared Socio-economic Pathways 126 and 585 (SSP 126/585), representing different carbon dioxide concentrations [75,76], was employed for the 2070s (only SSP 126/585 included full climate factors). LGM and LIG data from the WorldClim 1.4 database were calibrated for temperature data through ArcGIS, as the temperature value in the original data is 10 times the true value. Eight variables from four epochs (excluding MPWP and MIS 19, where six variables were retained, see Table S5) were retained for subsequent analysis (see Table S4). We excluded those variables with Pearson correlation coefficients r > 0.8 based on pairwise comparison of raster files in SDMtoolbox v2.0 [64]. We obtained 96 occurrence records of *E. argus* from the individuals sampled in this study, and 220 occurrence records downloaded from GBIF (https://www.gbif.org/ (accessed on 3 May 2022)) and the literature (e.g., [32,77,78,79]) (see Table S6). All sampling sites were also rarified at a spatial distance of 120 km using SDMtoolbox v2.0 [64], resulting in a total of 74 sampling sites (see Table S7). We used the maximum entropy algorithm to model the ecological niches of the species in MAXENT v3.4.4 [67], with the two sets of data from PaleoClim and WorldClim analyzed separately. In Maxent, 70% of the distribution data were randomly selected as the training set and 30% as the test set for 100 bootstrap replicates. We used the ENMeval package [80] in R to manage model complexity and determine the optimal combination of MaxEnt feature classes and regularization multipliers. The optimal model had a regularization multiplier of 3.0 and an LQHP features class. The remaining parameters were set as the default. To avoid over-extrapolation of the current and past ENM projections, we used a multivariate environmental similarity surface (MESS) analysis [81]. This analysis was performed to check whether the estimated projections contained combinations of climatic variables that were not represented in the training dataset. The MESS analysis indicates the locations of analogous and non-analogous habitats with respect to the training points [81]. Negative MESS values indicate areas where the projection is less reliable because at least one variable is outside the range found during calibration of the current model [81]. The area under the receiver operator characteristic curve (AUC) was used to assess the model reliability of the prediction results, ranging from 0.5 to 1.0, with AUC > 0.8 indicating a fair model. The potential suitable distribution area of each period was calculated in ArcGIS based on SDMtoolbox. The Maxent results were processed in ArcGIS v10.2.

## 3. Results

### 3.1. Sequence Characteristics and Phylogenetic Relationship

We generated a data set of 1043 bp. A total of 617 sequences (including three outgroups) yielded 282 haplotypes. The data set contained 761 invariable sites, 282 polymorphic sites and 195 parsimony-informative sites.

**Figure 2:**
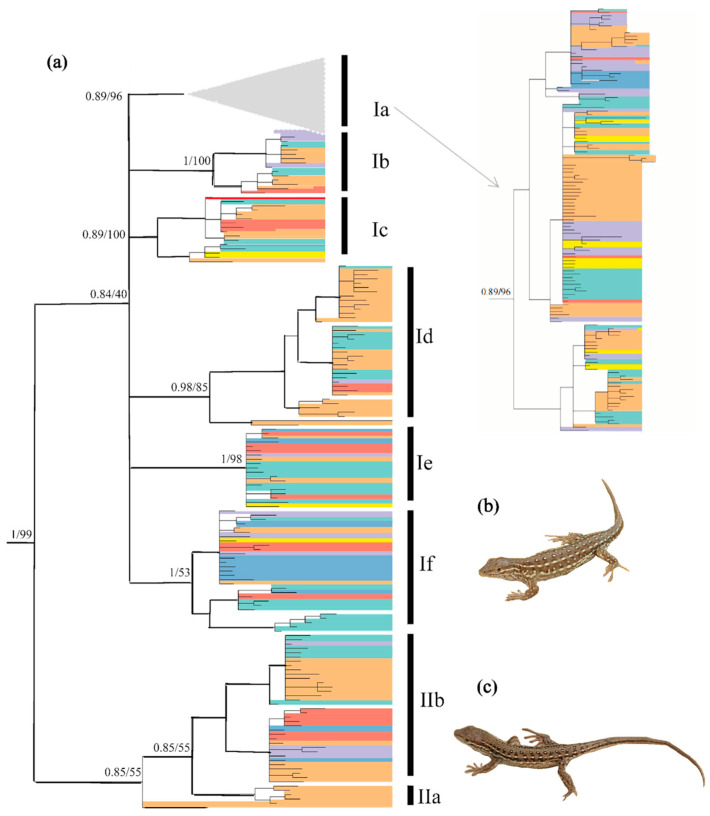
(**a**) Hypothesized phylogenetic relationships of *Eremias argus* included in this study, illustrated by the 50% majority-rule consensus tree resulting from partitioned Bayesian analyses. Bayesian posterior probabilities and maximum likelihood bootstrap values are shown. Distribution of the haplotype group across the species range refers to Figure 1; (**b**) *Eremais argus* with eye-like dorsal pattern; (**c**) *Eremais argus* with a striped dorsal pattern. Photos by X.G. (**b**,**c**) show two morphotypes of *Eremias argus*, and their reciprocal monophyly is rejected in the phylogenetic tree.

Bayesian inference and ML analyses produced a highly congruent topology, with only minor conflicts at recent nodes. Therefore, only the BI tree with both PP and UFBoot from ML is shown in Figure 2. Two clades with eight subclades were revealed. Subclade IIa contained only one haplotype. The relationship between clades I and II was resolved with strong support from PP (1.0) and UFBoot (100%). Owing to limited phylogenetically informative sites, the relationship of several subclades was not resolved, with relatively low PP values for Subclade Ia (0.89), Subclade Ic (0.89) and Subclade IIb (0.85), and low UFBoot values for Subclade Id (85%), Subclade If (53%) and Subclade IIb (55%). Most of these eight subclades did not correspond to the seven regions identified, and several haplotypes were shared by many individuals from different regions (see Figure S2).

### 3.2. Bayesian Test of Two Subspecies Hypothesis

We compared the marginal likelihood estimates of topologically constrained and unconstrained runs of MrBayes for possible monophyletic groups (subspecies) within *E. argus.* The marginal likelihood values of the constrained and unconstrained models were −10,508.45 and −9622.64, respectively. There was quite strong (BF > 1700) evidence against the constrained topologies. Thus, the alternative phylogenetic hypothesis—Two subspecies hypotheses—Was significantly rejected.

### 3.3. Timeframe for the Diversification of E. argus

**Figure 3:**
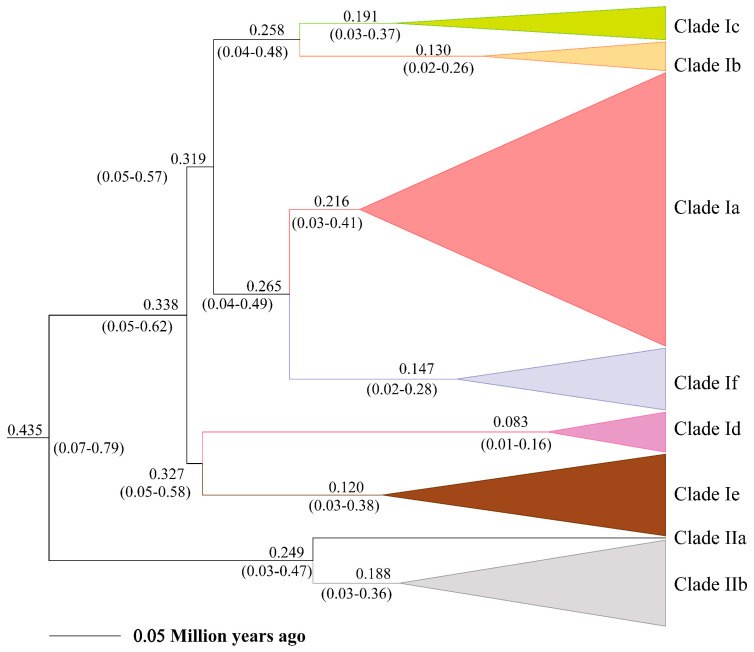
Estimate of divergence time of major lineages within *Eremias argus* derived from mtDNA cyt *b* data.

The posterior distribution of divergence times and their 95% highest posterior density interval (HPD) based on the phylogeny of Lacertidae and within *E. argus* are shown in Figure S1 and Figure 3, respectively. The divergence time between *E. argus* and *E. brenchleyi* was approximately 2.7 ± 0.6 Ma (95% HPD, 1.62–3.92 Ma), which is slightly younger than that reported by Zhao et al. [32]. The MRCA of *E. argus* was estimated to be about 0.4351 ± 0.1 Ma (95% HPD, 0.0719–0.7984 Ma). This is in agreement with the Pleistocene fossil record of *Eremias* cf. *argus* from the Qinling Mountains in China (493 ± 55 ka B.P.) [82]. Furthermore, the estimated age of the haplotypes from the Baikal region fauna fell within the timeframe of the fossil record of *Eremias* cf. *argus* in Transbaikalia of the Late Pleistocene and Holocene [83]. The mean substitution rate within *E. argus* was estimated to be 6.23%/site/million years, with a 95% HPD of 3.2–8.97%/site/million years. 

### 3.4. Environment, Geography, and Landscape Heterogeneity Impact on Genetic Structure

**Figure 4:**
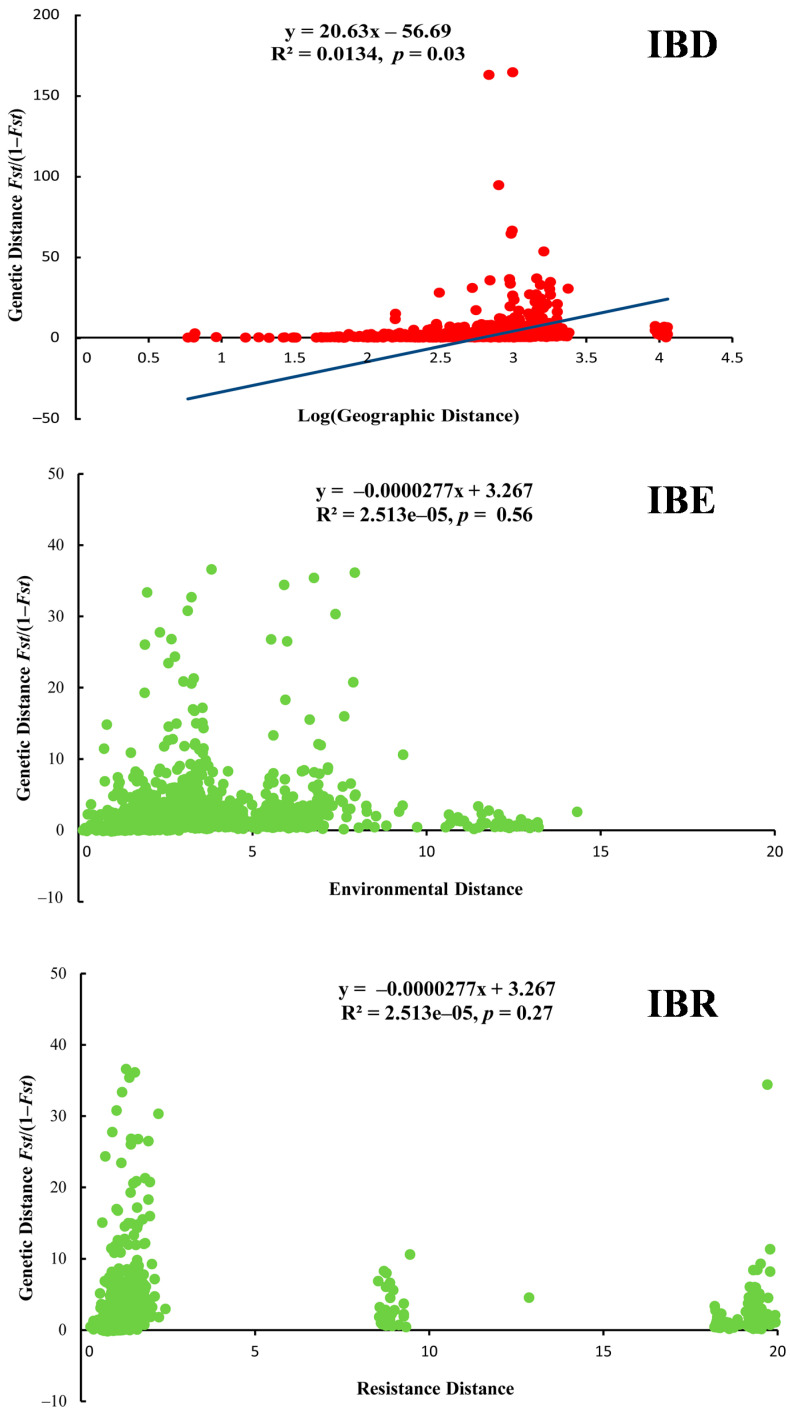
Correlations between genetic, environmental, geographic distances and resistance (IBD, IBE and IBR analysis results of *Eremias argus*).

For the Mantel test, we found that only IBD showed the significant positive correlation (r = 0.11, *p* = 0.03); IBE (r = −0.01, *p* = 0.56) and IBR (r = −0.05, *p* = 0.27) showed the non-significant negative correlation (Figure 4). Thus, geographic distance made an important contribution to shaping genetic structure, compared to the contributions of environment and landscape. For the partial Mantel test of IBD, based on the control of geographic barriers (e.g., Yellow River and Taihang Mts.), the value of r (0.44) and *p* (< 0.001) showed a significant positive correlation. Meanwhile, under the control of geographic distance, the value of r (−0.04) and *p* (0.72) was presented. Geographic barriers mainly affected the genetic structure. Moreover, the leading role of IBD also provided a reliable condition for simulating the migration of ancestral populations over time. For the partial Mantel test of IBE, based on the control of geographic distance (r = −0.001, *p* = 0.72) and the environmental distance (r = 0.11, *p* = 0.03), the correlation was presented. Bonferroni correction or other multiple testing methods were not used.

### 3.5. The Spatiotemporal Diffusion of E. argus

**Figure 5:**
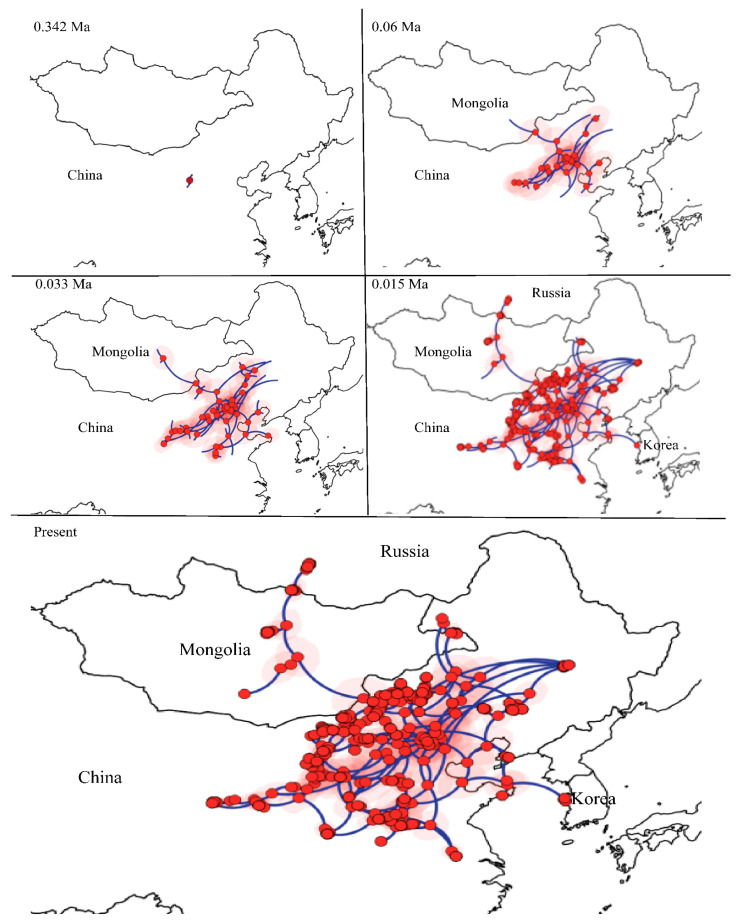
Spatiotemporal reconstruction of the geographic dispersal of *Eremias argus* using the Bayesian phylogeographic method. Nine snapshots indicate that *Eremias argus* originated in Northeast Asia.

We selected the BRW model as the best fitting diffusion model for the mitochondrial lineages of *E. argus* based on the value of BF and ESS (>200). Individuals of *E. argus* started to disperse from the NEA and then gradually dispersed in different directions, eventually to Mongolia, Russia and Korea. The most likely ancestral distribution of the MRCR of *E. argus* was located in Inner Mongolia. From 0.342 Ma to 0.06 Ma, the ancestral population began to disperse and then underwent regional dispersal in China. Around 0.06 Ma, *E. argus* moved northwards and reached Mongolia, spread eastwards and reached Shandong Province. From the end of the LIG to the LGM (~0.033 Ma), *E. argus* underwent the rapid dispersal. At ~0.015 Ma, *E. argus* populations moved westward to Gonghe County of Qinghai, northward to Russia, and the Shandong population moved eastward to Korea. As a result, the entire distribution of *E. argus* was represented (Figure 5).

### 3.6. Genetic Diversity and Genetic Structure

The nucleotide diversity (ND) of cyt *b* ranged from 0.0036 (Subclade Ib) to 0.027 (Subclade Ie) among eight subclades, and the HD of *E. argus* ranged from 0.396 (Ib) to 0.982 (Ia). The genetic diversity indices of the central region (Inner Mongolia) showed the highest haplotype diversity (0.965) and relatively higher nucleotide diversity (0.0213) than other regions except the southern region (0.0267). And these two indices of Mongolia (ND, 0.00397; HD, 0.881), Russia (ND, 0.00098; HD, 0.627), Gonghe County (ND, 0.000436; HD, 0.881) and Korea (10 same sequences) were all lower than those of other regions (Table S8). The variances between populations within seven groups and within populations were 34.98% (*Fct* = 0.34983, *p* < 0.001) and 33.11% (*Fct* = 0.050928, *p* < 0.001), respectively. The variance between groups was 31.9% (*Fct* = 0.68095, *p* < 0.001), indicating that different landscapes played an important role in shaping the genetic structure (see Table S8).

We used seven subclades excluding IIa which contained only one haplotype, to analyze the median-joining network (see Figure S2). The MJN of Subclade Ia produced a more complex pattern than other subclades, including two typical star-like networks; however, the two central haplotypes were not the most abundant in this subclade (Figure S2). One central haplotype (Hap32) was shared by individuals from Inner Mongolia and Northeast of China, and the other represented a missing haplotype. A similar network structure was present in the other six subclades, which showed no obvious geographic structure, but rather a star-like topology.

### 3.7. Historical Demographic Change

The subclades Ia, Id and IIb presented similar results for negative but statistically insignificant Tajima’s *D*, negative Fu’s *Fs* with statistically significant (see Table S9). For Subclade Ib, negative Tajima’s *D* were statistically significant and negative Fu’s *Fs* were statistically insignificant. For subclades Ie and If, the values of Fu’s *Fs* and Tajima’s *D* were both positive, with statistically significant Fu’s *Fs* for the subclade If, while with statistically insignificant values for Ie. Subclade Ic was the only one that presented non-significant positive Fu’s *Fs* and non-significant negative Tajima’s *D*. The MD result of all these seven subclades was multimodal, ragged and erratic (see Figure S3). Low and non-significant *Rg* and *SSD* values were presented in the subclades Ia and Ib. Low and significant *Rg* and *SSD* values were presented in the subclades Ic, Ie and If. Low and significant *Rg* and insignificant *SSD* values were presented in the subclades IIb and Id (see Table S9). The *R*_2_ values of these seven subclades were significant but large (>0.16).

**Figure 6:**
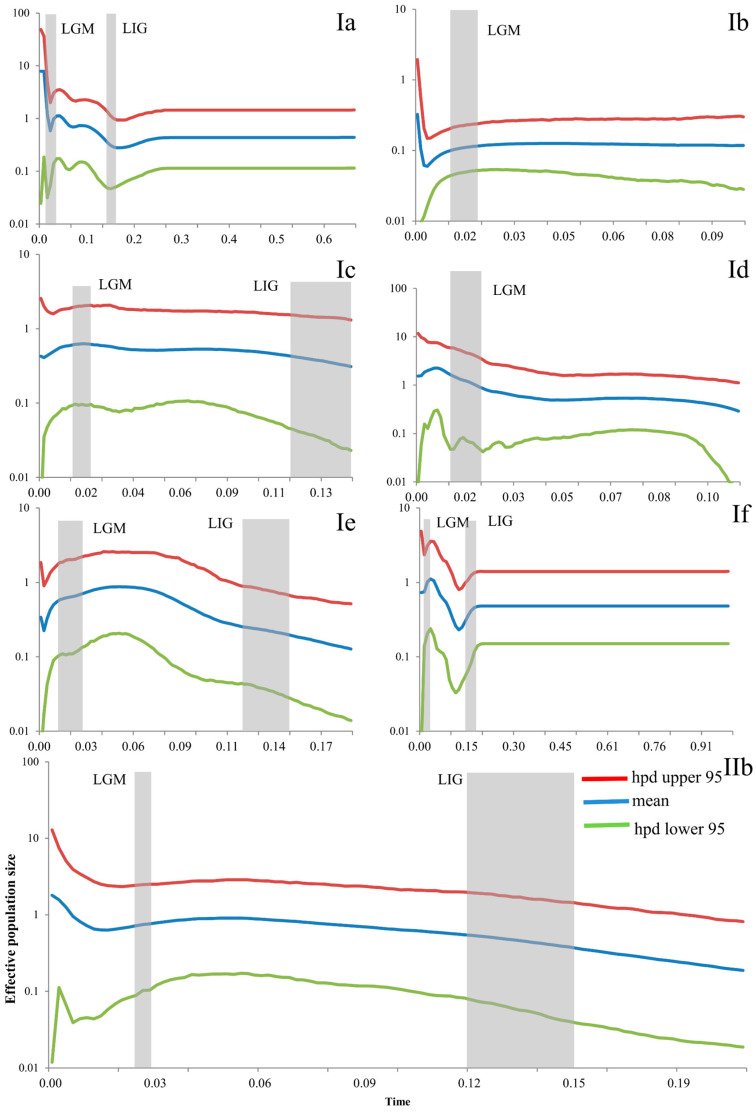
Bayesian skyline plots (BSP) of the seven subclades for *Eremias argus*. The y-axes represent the estimated effective population size on a log scale (*Ne* × τ/10^6^, the product of the female effective population size and generation length in years); x-axes represent time in millions of years ago (Ma). Vertical dark-grey bars represent the duration of the Last Glacial Maximum (LGM), or the Last Interglacial (LIG). **Ia**–**IIb** refers to the clade recovered in Figure 2.

Bayesian skyline plots of these seven subclades showed different patterns (Figure 6). Subclade Ia showed a slight population growth from the LIG to the LGM, and a rapid growth in the LGM. Subclade Ib showed a stable and almost decreasing pattern in the LGM and a population growth in the more recent period. Subclade Ic was stable from the LIG to the LGM and even in the present. Subclade Id also showed population growth during the LGM. Subclade Ie showed rapid growth from the LIG to the LGM and slight decrease after the LGM. Subclade If showed a population decline during the LIG and LGM, but a rather stable population before the LIG and a rapid expansion from the LIG to the LGM. The population of Subclade IIb increased slightly from the LIG to the LGM (Figure 6).

### 3.8. Temporal Changes of Suitable Distributional Areas

For the variables obtained from both WorldClim and PaleoClim, the three variables that contributed most to the distribution of *E. argus* were annual mean temperature (bio1, 38.28% in WorldClim and 41.61% in PaleoClim), annual precipitation (bio12, 21.50% in WorldClim and 20.13% in PaleoClim), and precipitation of driest month (bio14, 15.30% in WorldClim and 21.61% in PaleoClim) (see Tables S4 and S5). This indicates that temperature and humidity together influence the potential geographic distribution pattern of *E. argus*. Response curves for each environmental variable are presented in Figures S4 and S5, showing non-linear relationships between the probability of occurrence and environmental variables. The probability of occurrence became significant when the annul mean temperature was greater than 5 °C, and peaked when annual precipitation was approximately 200 mm, indicating that *E. argus* is adapted to arid and semi-arid temperate environments. 

**Figure 7:**
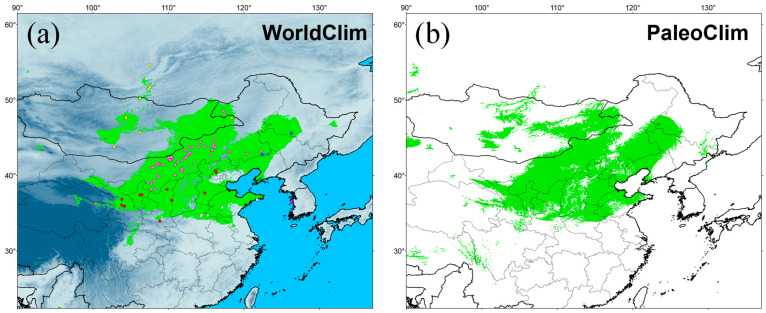
Potentially suitable distribution area obtained with MaxEnt for *Eremias argus* at present. Green indicates highly suitable habitat area. Climate variables are from WorldClim (**a**) and PaleoClim (**b**).

**Figure 8:**
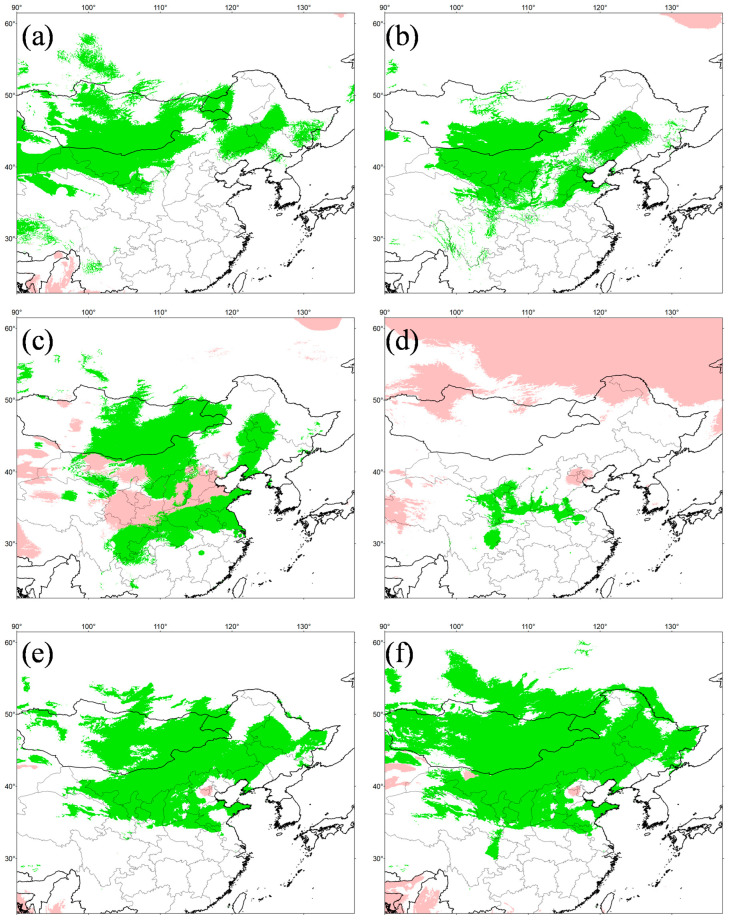
Potentially suitable distribution area in six different periods for *Eremias argus*. (**a**) MPWP, Mid-Pliocene Warm Period; (**b**) MIS 19, Marine Isotope Stage 19; (**c**) LIG, Last Interglacial (**d**) LGM, Last Glacial Maximum; (**e**) SSP 126 for 2070s, Shared Socio-economic Pathway 126; (**f**) SSP 585, Shared Socio-economic Pathway 585 for 2070s. (**a**,**b**) show the separate projections for MPWP and MIS 19 by running the model in current times with the variables in Paleoclim. (**c**–**f**) show the separate projections for LIG, LGM, SSP 126 and SSP 585 by running the model in current times with the variables in Wordclim. Negative MESS scores, shown as similarity <0 by pink, indicate areas without current equivalents of climatic conditions.

The simulation results were highly credible, as the AUC values for the WorldClim and PaleoClim data were 0.945 ± 0.007 and 0.940 ± 0.009, respectively. The maximum logistic thresholds for sensitivity and specificity were 0.3088 and 0.3652, respectively. The simulation results of WorldClim and PaleoClim showed a similar geographic distribution pattern in the present (Figure 7), with the highly suitable habitat of *E. argus* mainly in northern China and partly located in Mongolia and Russia, which is consistent with the occurrence data. The results also suggest that mountains may be barriers to the distribution of *E. argus*, such as the Greater Khingan Mountains and the Taihang Mountains (see Figure 1). The total highly suitable habitat area (HSHA) is 2.24 × 10^6^ km^2^ and 2.20 × 10^6^ km^2^ for the WorldClim and PaleoClim data, respectively. In the LIG period, the total HSHA was 3.24 × 10^6^ km^2^ in total, which was a larger area compared to the present (Figure 8). During the coldest period, the LGM, the HSHA decreased significantly to 0.37 × 10^6^ km^2^, existing only in central China. And after 50 years from now, in the 2070s, the HSHA of *E. argus* will increase significantly, whether under SSP 126 or SSP 585 situations, mainly manifested as further expansion towards the north. The total HSHA is 3.54 × 10^6^ km^2^ under SSP 126 and 5.49 × 10^6^ km^2^ under SSP 585. In MIS 19, when the barrier effect of the mountains was more pronounced, the HSHA was 2.47 × 10^6^ km^2^. The northward shift of the HSHA occurred in the MPWP period, mainly in the arid regions of the north, where it was 3.00 × 10^6^ km^2^.

In addition, the MESS quantitative measure showed a high similarity of climate conditions between the current and other different scenarios, except the LIG, providing the reliability of the simulation results.

## 4. Discussion

### 4.1. Phylogeographic Structure of E. argus and Its Drivers

Bayesian phylogenetic analysis suggested that *E. argus* is composed of eight undifferentiated geographic lineages, albeit with relatively low PP and UFBoot values. A similar population genetic structure unrelated to geographic region is also reflected in a widely distributed toad, *Bufo gargarizans*, throughout East and Northeast Asia [84]. Previous studies of *E. argus* [31,32], based on limited numbers of individuals (<130) and one gene, respectively, also showed no geographic specificity. We propose two explanations for the weak phylogeographic pattern of *E. argus* in relation to its ecological adaptation. First, *E. argus* is a habitat generalist, occurring in forests, shrublands, grasslands, wetlands, rocky areas, and deserts [26]. This allows it to disperse over long distances over land compared to its sister species (*E. brenchleyi*). These characteristics of *E. argus* have probably played an important role in its widespread distribution across the NEA. If the historical range expansion involved many individuals and occurred soon after genetic differentiation, the undifferentiated phylogeographic pattern observed could result. Second, we suggest that anthropogenic impacts have contributed to the lack of a clear phylogeographic pattern in *E. argus*. In China, *E. argus* has been used in traditional Chinese medicine for thousands of years [85]. More recently, *E. argus* has also been used as a pet or as food for snakes. A recent online search for lizard farms or lizard fun reveals many sites operating in different regions of China. Any escape or release of translocated individuals, followed by reproduction with native individuals would contribute to obscuring phylogeographic patterns. Similar hypotheses have been proposed for the weak phylogeographic pattern of the Asian toad in relation to its adaptations to environmental stressors [84].

In our study, analyses of IBD, IBR and IBE were implemented and compared. Compared to environmental and climatic resistance, only the geographic distance showed a positive correlation with genetic diversity, which differs from the perspective of Zhao et al. [32], who only used the IBD analysis. Thus, isolation-by-distance has a strong effect on the divergence of populations across the distribution range.

### 4.2. Out of Inner Mongolia: The Spatial-Temporal Evolution of E. argus

This work provides the first evidence that the modern populations of *E. argus* may have originated in Inner Mongolia before dispersing and diversifying over a wide area of the NEA. The highest diversity indices of Inner Mongolia also support the origin hypothesis, which contradicts with the tentative hypothesis of Zhao [86], who considered Central Asia as a possible origin of *E. argus* without providing reliable evidence. The estimated divergence time of *E. argus* and *E. brenchleyi* was 2.7 Ma (95% HPD, 1.62–3.92 Ma), which is younger than that of Zhao et al. [32], i.e., 4.1 Ma with the 95% credible interval (CI) ranging from 2.4 Ma to 6.8 Ma. Despite the slight difference, our estimates of divergence were also within the 95% CI of the estimate of Zhao et al. [32]. On the other hand, this study presents a more precise result, based on more credible calibration points and a sufficient range-wide sampling dataset.

The method of phylogeographic diffusion has been widely used for many species without obvious range barriers to find the most likely distribution of the most recent common ancestor, and to show the dispersal route (e.g., [87]). We therefore used this model to predict the colonization routes of *E. argus* (Figure 5). The onset of dispersal from Inner Mongolia (0.342 Ma) roughly followed the time of divergence of Clade I and Clade II (0.4351 Ma). The warm and humid climate during the LIG may have facilitated the dispersal events from the end of the LIG into the LGM (~0.033 Ma). After the LGM (~0.015 Ma), the distribution of *E. argus* expanded greatly. *E. argus* also reached Gonghe County (Qinghai), Russia and Korea also around this time (0.015 Ma). Recently, young populations have been established, which is consistent with the low genetic diversity of the populations in Gonghe, Russia and Korea. Among these regions, *E. argus* has been recognized as an endangered species in Korea [88,89]. The Korean Peninsula population originated recently from Shandong Province, as reflected by the tip position of the Korean Peninsula haplotype in the MJN (red in Ic; Figure S2). On the other hand, the Korea population is more similar to the Shandong population based on morphological characters, i.e., the eye-like dorsal pattern. Therefore, we concluded that the Korea population is derived from the Shandong population. Future studies based on multiple nuclear loci are highly desirable. Such studies will not only confirm or falsify the phylogeographic patterns of this model species suggested by our mtDNA data but will also be able to reveal details of the genetic characteristics (cline shape, width, fitness) within these putative contact zones.

Low temperatures and arid climates caused by Quaternary climate oscillations and the uplift of the QTP [90,91] may have led to a reduction in suitable habitats for *E. argus* in MIS 19 and the LGM. The extensive uplift of the QTP has influenced the process of aridity in inland of Asia by strengthening the monsoon circulation, blocking the warm and humid air currents of the Indian Ocean, and influencing the combination of the winter and summer monsoons of East Asia [92]. From 3.6 Ma to 2.6 Ma (MPWP), the East Asian winter and summer monsoons strengthened simultaneously, and monsoon-induced precipitation increased. At the same time, the distribution of *E. argus* is at its maximum. From 2.6 Ma to the present, the East Asian winter monsoon strengthened and the summer monsoon was weakened, resulting in a decrease in precipitation. During this period, the suitable distribution decreased compared to that of the MPWP. The change in the contribution of different variables coincided with the change in the suitable range of different periods. During the LGM, the temperature decreased; thus, the contribution of different quarters was different before the LGM. Considering the effect of hydric conditions on embryonic and offspring survival of lizards [93], it is reasonable to predict the significant role of precipitation in different quarters.

According to the literature [23] and GBIF data, the distribution of *E. argus* in North Korea was quite limited. Interestingly, the suitable range in the Korean Peninsula shrank and even disappeared from the MPWP to the MIS 19. However, the suitable range associated with the Korean Peninsula and Shandong reappeared during the LIG (Figure 8). The Korean Peninsula was characterized by a subtropical mountain climate [94] and was not greatly affected by global climate changes during the Pleistocene, which probably provided favorable conditions for *E. argus* to serve as a suitable range connected to the Shandong Peninsula for the exposure of large continental shelves. Thus, we postulate that *E. argus* may have become extinct on the Korean Peninsula before the LIG, and the Shandong population then migrated to the Korean Peninsula after the LGM (Figure 5).

### 4.3. Pre-LGM Expansion for Most Lineages of E. argus

Temperate East Asia was dominated by summer precipitation and did not develop large glaciers during major glacial periods [95]. Milder climate cycles may have resulted in different phylogeographic patterns in East Asian species than in Europe and North America. Several recent studies have suggested that the past distributions of species in East Asia during the LIG and LGM periods underwent a so-called ‘pre-LGM expansion’ [96]. This refers to the unusually similar distribution patterns between the current and the LGM scenarios. This has been further supported by studies of the phylogeography and demographic history of Southeast Asian species [96,97,98,99].

One of the key findings of the present study is that the paleo-distributions of *E. argus* are consistent with the ‘pre-LGM expansion’ pattern, which is further supported by its demographic history. BSP results indicated recent expansions for most subclades (except Ic) of *E. argus.* Furthermore, Bayesian diffusion analysis indicated that populations started to disperse rapidly from the end of the LIG to the LGM (0.033 Ma), and this scenario was consistent with BSP results, which indicated population expansions from the end of the LIG to the LGM for all subclades except Subclade Ib (Figure 6). The pre-LGM expansion mode of *E. argus* was inconsistent with a previous study [31], which proposed that the population expansion event started about 4400 years ago. Due to the limited data and insufficient sampling, the results of Qu et al. [31] may have been misleading. In addition, the limited calibration points used for molecular dating in Qu et al. [31] may also have biased the BSP analysis.

However, based on the ENM results, the HASA of *E. argus* seems to have decreased during the LGM compared to those during the LIG (Figure 8), which seems to be inconsistent with those of the Bayesian diffusion analysis and the BSP analysis. In addition, the current distribution of *E. argus* seems to be more similar to its LIG distribution than to that of the LGM. This discrepancy could be plausibly explained as follows. We acknowledge that the demography of glacial population expansion is not only consistent with the ecology of *E. argus*, but also fits well with the environmental history of Northeast Asia. During the LGM, climate change in these regions appeared to be moderate, as indicated by limited glacial expansion in nearby mountains [95,100]. This would have alleviated the low temperature stress associated with glacial expansion. Additionally, because *E. argus* has developed a strong adaptation to a diverse habitat, the aridification caused by glaciers would not put much stress on its persistence. On the other hand, the subsequent strengthening of the monsoon in East Asia since the mid-Late Pleistocene would have created a variety of new habitats for the “habitat generalist” [101]. As expected, the expansion of steppe-desert fields during glacial periods would have provided *E. argus* with a suitable matrix for burrowing, allowing them to effectively buffer the climate extremes. Consequently, although the overall size of their HSHA was reduced during the LGM (Figure 8), the localized effective niche spaces may have expanded with the development of the monsoons in East Asia since the mid-Late Pleistocene. In addition, *E. argus* can maintain extremely high biomass in diverse communities, so it is reasonable to assume that even at the LGM, population densities were well below the carrying capacity of the habitats, allowing *E. argus* to expand. In any case, there was no apparent refugium during the LGM, contradicting the previous “multiple refugia hypothesis” derived from haplotype group splitting [31,32]. Similar trends have also been observed in plants [102,103], the tree frog *Hyla sarda* [104], and some insects, including the semi-aquatic beetle *Microvelia douglasi douglasi* [98].

Our results suggest that *E. argus* populations were probably not adversely affected by recent glaciations during the Late Pleistocene to Holocene and did not experience drastic population size declines. This was probably due to the development of monsoons in East Asia and the lowering of the elevation of eastern China since the mid-Late Pleistocene [100], which increased the localized effective niches for *E. argus*. Overall, our results suggest a significant role of the LIG and LGM in shaping the demographic history of the Mongolian racerunner; this study thus contributes to the growing idea that species’ responses to Pleistocene climate fluctuations were more diverse than previously thought.

### 4.4. Implications for the Infraspecific Taxonomy

Historically, the category of subspecies has been used to diagnose geographically distinct populations that were thought to be in the early stages of speciation [105,106]. Overall, genetic studies of a wide range of organisms have found that morphologically defined subspecies often correspond to phylogenetic lineages (e.g., [107]). The finding that many subspecies correspond to distinct phylogenetic lineages has often led to the (usually unstated) assumption that most or all currently recognized subspecies are evolutionarily coherent and divergent [108,109].

*Eremias argus* exhibits considerable intraspecific variation, with two subspecies, *E. a. argus* Peters, 1869 and *E. a. barbouri* Schmidt, 1925. Furthermore, as documented by Zhao et al. [26], the distribution range of *E. a. argus* included the eastern part of Inner Mongolia, Heilongjiang, Jilin, Liaoning, Shandong, Anhui, Jiangsu, part of Hebei, the eastern of Mongolia, Russia and Korea, and the range of *E. a. barbouri* included part of Inner Mongolia, Shanxi, Shannxi, Ningxia, Gansu, Henan and part of Hebei. However, we found that the two subspecies, which were distinguished by morphological characters of dorsal pattern and midbody scales, did not coincide with the range proposed by Zhao et al. [26].

We found that the distribution of the two forms was not geographically specific as intended by Zhao et al. [26] and Szczerbak [29]. Some sampled sites hosted both forms. In addition, there are a few individuals in the sampled specimens that show intermediate patterns between a clean eye-like pattern and a clean striped pattern for the dorsal pattern. For instance, two forms coexist in the Abaga Banner population of Inner Mongolia. Notably, the reciprocal monophyly of both subspecies was statistically rejected based on hypothesis testing using the Bayes factor. Thus, we find a weak and inconsistent correspondence between morphological patterns and putative subspecies ranges. Furthermore, we were unable to reconcile the genetic lineages and spatial character transitions we identified with subspecies ranges as currently understood.

Taken together, it seems unlikely that the name *E. a. barbouri* refers to a distinct entity. At present, recognizing *E. argus* as a widely distributed species with no subspecific taxa appears to best convey the evolutionary history of the species and the often spatially idiosyncratic patterns of phenotypic variation. We believe that this fundamentally conservative arrangement is preferable to recognizing subspecies whose unclear boundaries, distributions and morphological diagnoses have confused many herpetologists working in the field and in natural history collections.

## 5. Conclusions

*Eremias argus* is a genetically diverse species with a wide distribution across Northeast Asia. To our knowledge, the present study is the first to report the phylogeography of a reptile over such a broad region in NEA. We show the complex genetic pattern of this species, where most genetic divergence is not associated with geographic regions. We suggest that this pattern is the result of multiple influences—robust dispersal ability resulting from ecological features, anthropogenic influence of translocation, and past climate change. A strong pattern of isolation by distance across all populations suggests that geographic distance has primarily shaped the genetic structure of the species. The population growth of *E. argus* from the LIG to the LGM is likely a consequence of an increase in favorable habitats (localized effective niches) with mild climates following the development of monsoons in East Asia since the mid-Late Pleistocene. Overall, the population genetic structure of *E. argus* was likely driven by a combination of climatic and geographic changes rather than direct ice sheet coverage. The scenario of non-refugium and population expansion from the LIG to the LGM that we propose here may provide insight into understanding the origin and maintenance of biodiversity in other hotspots of climatic complexity. Future research using population genomics and/or landscape genomics, together with adequate and dense sampling across the range, will fully unravel the mechanisms underlying genetic divergence, population dynamics and adaptive evolution of *E. argus*.

## Data Availability

The data supporting the results of this study can be found in the manuscript. All sequences generated during this study have been deposited in GenBank (https://www.ncbi.nlm.nih.gov/genbank/, accessed on 28 December 2023).

## Supplementary Materials

The following supporting information can be downloaded at: https://www.mdpi.com/article/10.3390/ani14071124/s1, Figure S1: Molecular dating of the most recent common ancestor (MRCA) for *Eremias argus* using calibrations in outgroups. Orange circles indicated the different calibration points. Figure S2: Median-joining networks of mtDNA cyt *b* haplotypes of *Eremias argus*. Distribution of haplotype group by species entire range refers to Figure 1. Short bars crossing network branches indicate mutation steps; small dark circles indicate median vectors inferred by PopART v1.7 software. Circle size corresponds to the relative number of individuals sharing a particular haplotype, with the smallest in each clade being one individual. Figure S3: Mismatch distributions (MD) analysis for several subclades of *Eremias argus*. The green line corresponds to the expected frequencies of sudden population expansion, while the red line corresponds to the observed values. Figure S4: Response curves for each environmental variable from WorldClim in ENM when set all other environmental variables at their average sample value. The curves show the mean response of the 100 replicate Maxent runs (red) and the mean +/− one standard deviation (blue). Figure S5: Response curves for each environmental variable from PaleoClim in ENM when set all other environmental variables at their average sample value. The curves show the mean response of the 100 replicate Maxent runs (red) and the mean +/− one standard deviation (blue). Table S1: Samples information and haplotype numbers of *Eremais argus* in this study. Table S2: Data on the phylogenetic, molecular dating, Bayesian phylogeographic diffusion and BSP analyses, including partitions, models and parameters. Table S3: Sequences of Lacertidae and three outgroups retrieved form GenBank. Table S4: Climatic factors from WorldClim used in ENM and their contribution rates. Table S5: Climatic factors from PaleoClim used in ENM and their contribution rates. Table S6: Occurrence records with coordinates for *Eremias argus* retrieved from literature and GBIF. Table S7: Coordinates of *Eremais argus* used for ecological niche modeling. Table S8: Hierarchical analysis of AMOVA for *Eremias argus*. Table S9: Descriptive statistics by subclades/clades of *Eremias argus*. References [110,111,112,113,114,115,116,117,118,119,120,121,122,123,124,125,126,127,128,129,130,131,132,133,134,135,136,137,138,139,140,141,142,143,144,145,146,147,148,149,150,151,152,153,154,155,156,157,158,159,160,161,162,163,164,165,166,167,168,169,170,171,172,173,174,175,176,177] are cited in Table S3, Table S6 and Table S7.
