# Supplementary material for: Range-Wide Phylogeography and Ecological Niche Modeling Provide Insights into the Evolutionary History of the Mongolian Racerunner (Eremias argus) in Northeast Asia
Source: Animals (Basel). 2024 Apr 7;14(7):1124. doi: 10.3390/ani14071124 (PMC11011046; doi:10.3390/ani14071124)
Supplement: Supplementary file 1 [file animals-14-01124-s001.zip › animals-2927375-supplementary.pdf]

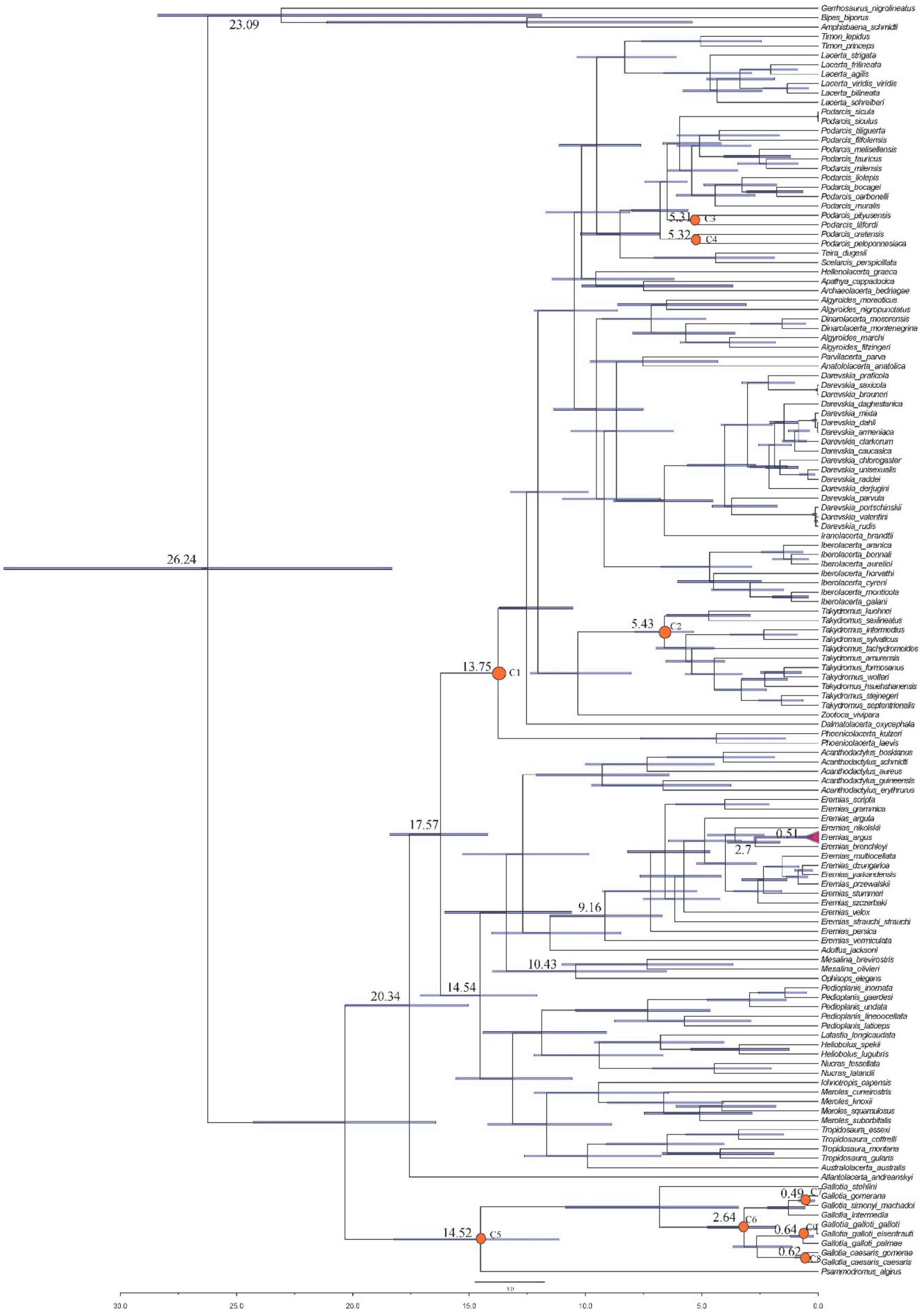

Millions of years ago

Figure S1. Molecular dating of the most recent common ancestor (MRCA) for *Eremias argus* using calibrations in outgroups. Orange circles indicated the different calibration points.

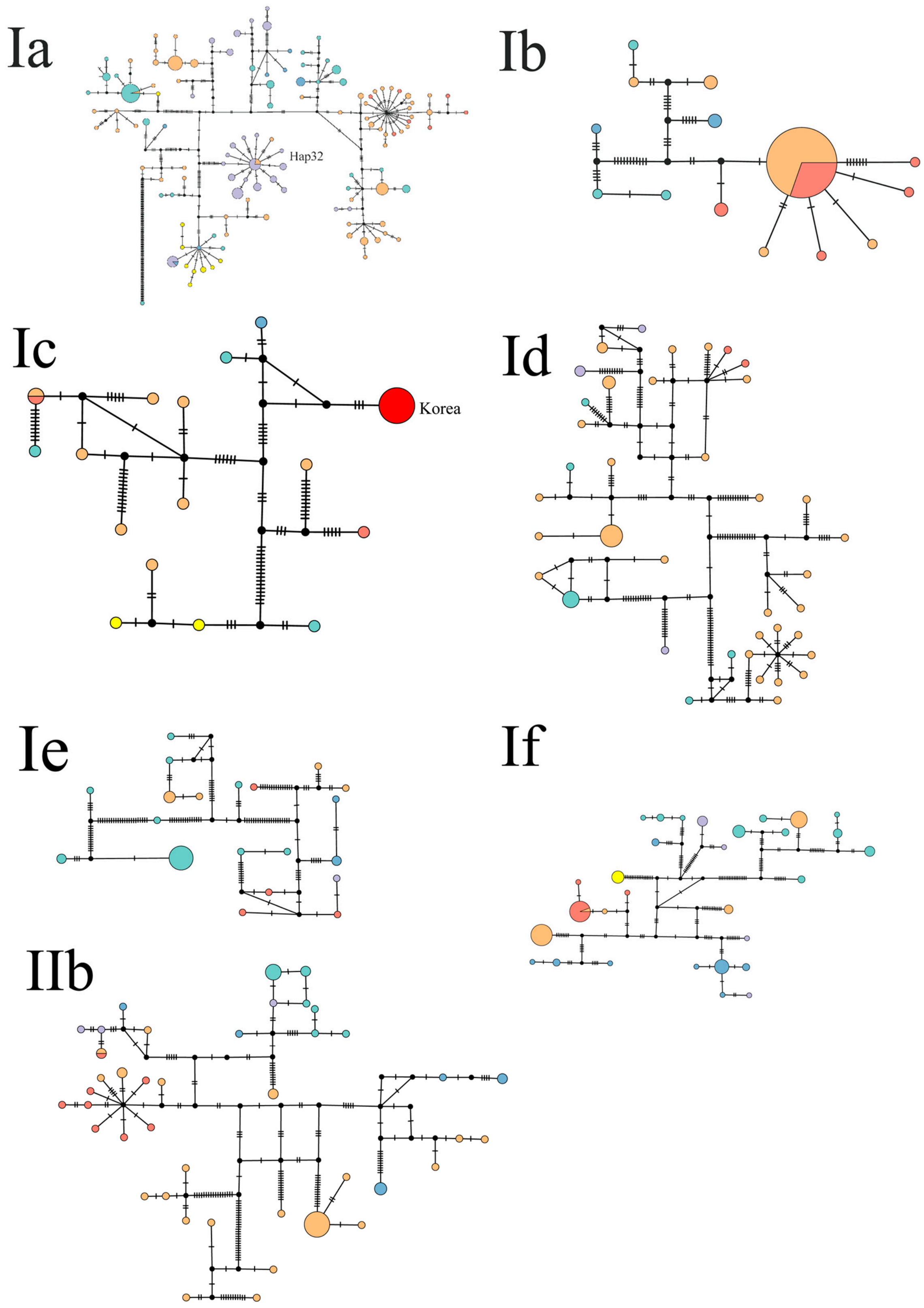

Figure S2. Median-joining networks of mtDNA cyt *b* haplotypes of *Eremias argus*. Distribution of haplotype group by species entire range refers to Figure 1. Short bars crossing network branches indicate mutation steps; small dark circles indicate median vectors inferred by PopART v1.7 software. Circle size corresponds to the relative number of individuals sharing a particular haplotype, with the smallest in each clade being one individual.

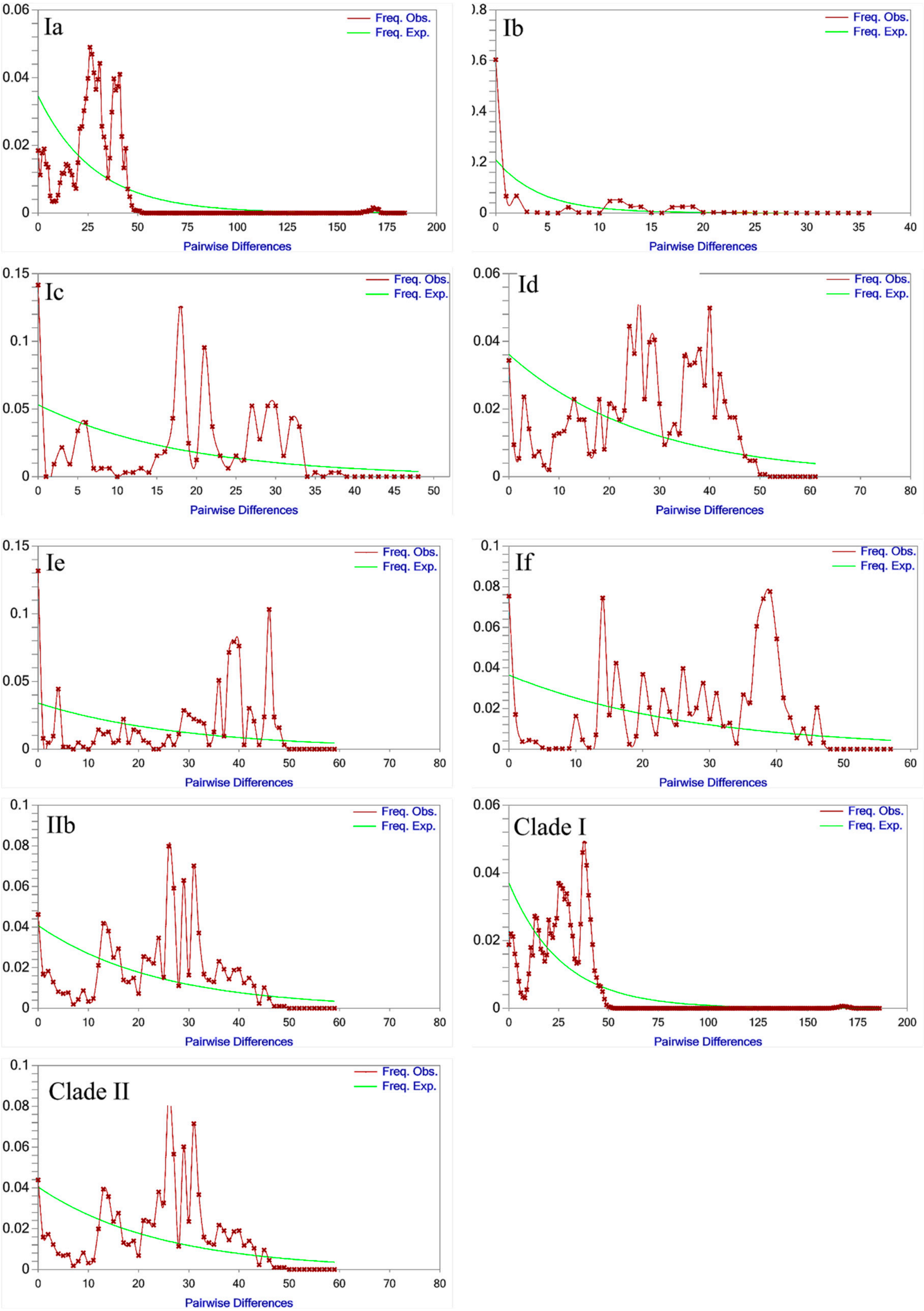

Figure S3. Mismatch distributions (MD) analysis for several subclades of *Eremias argus*. The green line corresponds to the expected frequencies of sudden population expansion, while the red line corresponds to the observed values.

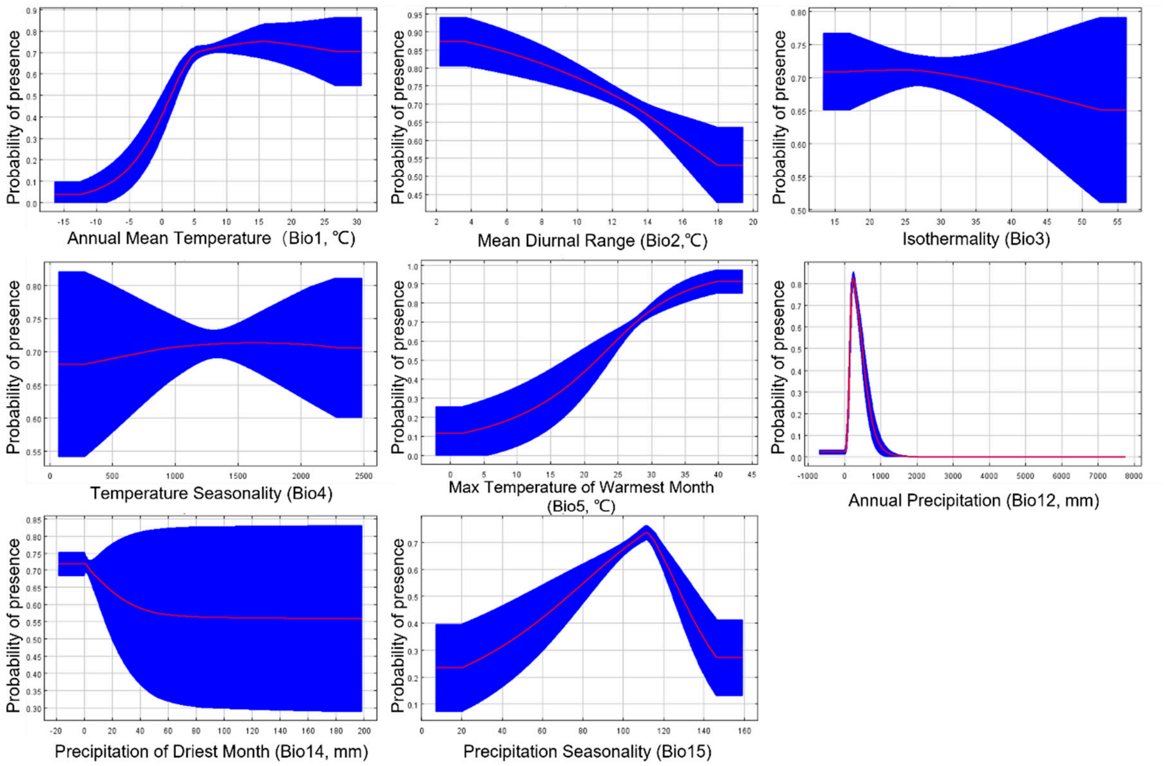

Figure S4. Response curves for each environmental variable from WorldClim in ENM when set all other environmental variables at their average sample value. The curves show the mean response of the 100 replicate Maxent runs (red) and the mean +/– one standard deviation (blue).

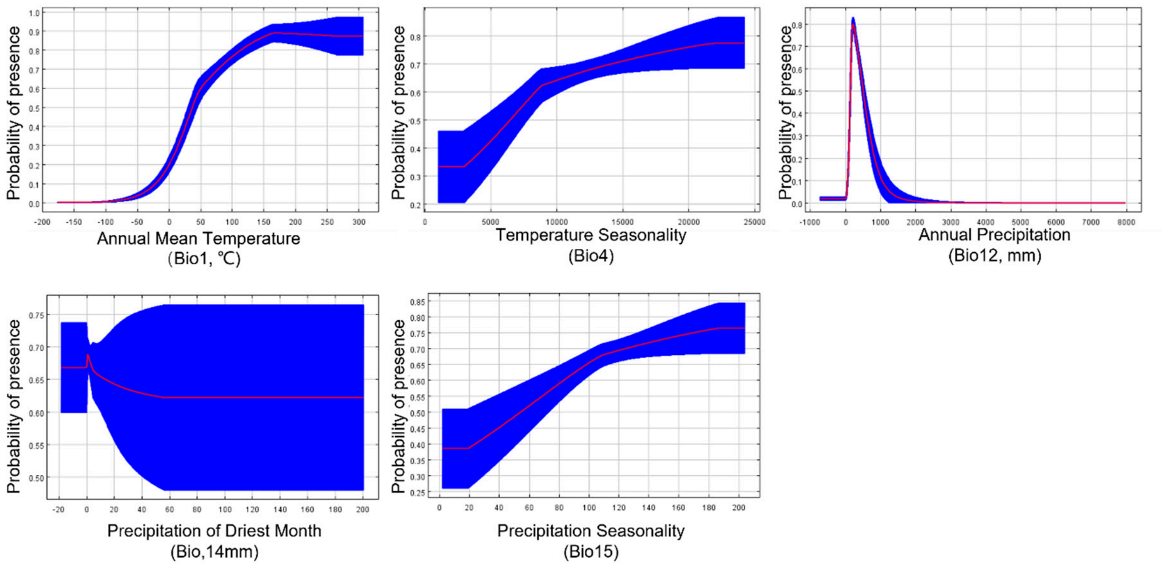

Figure S5. Response curves for each environmental variable from PaleoClim in ENM when set all other environmental variables at their average sample value. The curves show the mean response of the 100 replicate Maxent runs (red) and the mean +/– one standard deviation (blue).

Table S1 Samples information and corresponded haplotype numbers of *Eremias argus* used for phylogenetic analysis in this study.

Subspecies	Sample number	Haplotype	Subclade	Latitude (N)	Longitude (E)	Country	Geographical origin	GenBank accession no.
E. argus argus	EA2_CZ1	Hap102	Ia	32.3	118.3	China	Chuzhou City, Anhui	HM120774
E. argus argus	EA2_CZ2	Hap102	Ia	32.3	118.3	China	Chuzhou City, Anhui	HM120774
E. argus argus	EA2_CZ3	Hap102	Ia	32.3	118.3	China	Chuzhou City, Anhui	HM120775
E. argus argus	EA2_CZ4	Hap102	Ia	32.3	118.3	China	Chuzhou City, Anhui	HM120776
E. argus argus	EA2_CZ5	Hap102	Ia	32.3	118.3	China	Chuzhou City, Anhui	HM120776
E. argus argus	EA3_CZ6	Hap103	Id	32.3	118.3	China	Chuzhou City, Anhui	HM120763
E. argus argus	EA42_CZ7	Hap139	Id	32.3	118.3	China	Chuzhou City, Anhui	HM120776
E. argus argus	EA42_CZ8	Hap139	Id	32.3	118.3	China	Chuzhou City, Anhui	HM120784
E. argus argus	EA42_CZ9	Hap139	Id	32.3	118.3	China	Chuzhou City, Anhui	HM120763
E. argus argus	EA42_CZ10	Hap139	Id	32.3	118.3	China	Chuzhou City, Anhui	HM120763
E. argus argus	EA42_CZ11	Hap139	Id	32.3	118.3	China	Chuzhou City, Anhui	HM120763
E. argus argus	EA43_CZ12	Hap140	Id	32.3	118.3	China	Chuzhou City, Anhui	HM120763
E. argus argus	EA44_CZ13	Hap141	Id	32.3	118.3	China	Chuzhou City, Anhui	HM120774
E. argus argus	Guo1858	Hap82	If	33.01	112.52	China	Funiushan Mountain National Nature Reserve, Nanyang City, Henan	OR019270
E. argus argus	Guo1859	Hap82	If	33.01	112.52	China	Funiushan Mountain National Nature Reserve, Nanyang City, Henan	OR019271
E. argus barbouri	Guo1532	Hap72	If	33.98	108.83	China	Zhuxiping village, Xi'an City, Shaanxi	OR019207
E. argus barbouri	Guo1496	Hap71	Ia	33.99	108.84	China	Liyuanping village, Xi'an City, Shaanxi	OR019186
E. argus barbouri	Guo1497	Hap71	Ia	33.99	108.84	China	Liyuanping village, Xi'an City, Shaanxi	OR019187
E. argus barbouri	Guo1502	Hap71	Ia	33.99	108.84	China	Liyuanping village, Xi'an City, Shaanxi	OR019190
E. argus barbouri	Guo1503	Hap71	Ia	33.99	108.84	China	Liyuanping village, Xi'an City, Shaanxi	OR019191
E. argus barbouri	Guo1506	Hap71	Ia	33.99	108.84	China	Liyuanping village, Xi'an City, Shaanxi	OR019192
E. argus barbouri	Guo1508	Hap71	Ia	33.99	108.84	China	Liyuanping village, Xi'an City, Shaanxi	OR019194

E. argus barbouri	Guo1509	Hap71	Ia	33.99	108.84	China	Liyuanping village, Xi’an City, Shaanxi	OR019195
E. argus barbouri	Guo1510	Hap71	Ia	33.99	108.84	China	Liyuanping village, Xi’an City, Shaanxi	OR019196
E. argus barbouri	Guo1511	Hap71	Ia	33.99	108.84	China	Liyuanping village, Xi’an City, Shaanxi	OR019197
E. argus barbouri	Guo1514	Hap71	Ia	33.99	108.84	China	Liyuanping village, Xi’an City, Shaanxi	OR019200
E. argus barbouri	Guo1519	Hap71	Ia	33.99	108.84	China	Liyuanping village, Xi’an City, Shaanxi	OR019204
E. argus barbouri	Guo1600	Hap71	Ia	33.99	108.84	China	Liyuanping village, Xi’an City, Shaanxi	OR019217
E. argus barbouri	Guo1513	Hap74	Ia	33.99	108.84	China	Liyuanping village, Xi’an City, Shaanxi	OR019199
E. argus barbouri	Guo1515	Hap75	Ia	33.99	108.84	China	Liyuanping village, Xi’an City, Shaanxi	OR019201
E. argus barbouri	Guo1602	Hap81	Ie	33.99	108.84	China	Liyuanping village, Xi’an City, Shaanxi	OR019218
E. argus barbouri	Guo1498	Hap72	If	33.99	108.84	China	Liyuanping village, Xi’an City, Shaanxi	OR019188
E. argus barbouri	Guo1500	Hap72	If	33.99	108.84	China	Liyuanping village, Xi’an City, Shaanxi	OR019189
E. argus barbouri	Guo1507	Hap72	If	33.99	108.84	China	Liyuanping village, Xi’an City, Shaanxi	OR019193
E. argus barbouri	Guo1512	Hap72	If	33.99	108.84	China	Liyuanping village, Xi’an City, Shaanxi	OR019198
E. argus barbouri	Guo1517	Hap72	If	33.99	108.84	China	Liyuanping village, Xi’an City, Shaanxi	OR019202
E. argus barbouri	Guo1520	Hap72	If	33.99	108.84	China	Liyuanping village, Xi’an City, Shaanxi	OR019205
E. argus barbouri	Guo1518	Hap73	If	33.99	108.84	China	Liyuanping village, Xi’an City, Shaanxi	OR019203
E. argus barbouri	EA4_CA1	Hap104	IIb	34	108.9	China	Chang’an District, Xi’an City, Shaanxi	HM120774
E. argus barbouri	EA4_CA2	Hap104	IIb	34	108.9	China	Chang’an District, Xi’an City, Shaanxi	HM120775
E. argus barbouri	EA5_CA3	Hap105	IIb	34	108.9	China	Chang’an District, Xi’an City, Shaanxi	HM120776
E. argus barbouri	EA6_CA4	Hap106	IIb	34	108.9	China	Chang’an District, Xi’an City, Shaanxi	HM120761
E. argus barbouri	EA6_CA5	Hap106	IIb	34	108.9	China	Chang’an District, Xi’an City, Shaanxi	HM120761
E. argus barbouri	EA6_CA6	Hap106	IIb	34	108.9	China	Chang’an District, Xi’an City, Shaanxi	HM120761
E. argus barbouri	EA6_CA7	Hap106	IIb	34	108.9	China	Chang’an District, Xi’an City, Shaanxi	HM120761
E. argus barbouri	EA6_CA8	Hap106	IIb	34	108.9	China	Chang’an District, Xi’an City, Shaanxi	HM120761
E. argus barbouri	EA23_CA9	Hap123	Ia	34	108.9	China	Chang’an District, Xi’an City, Shaanxi	HM120761
E. argus barbouri	EA23_CA10	Hap123	Ia	34	108.9	China	Chang’an District, Xi’an City, Shaanxi	HM120761
E. argus barbouri	EA52_CA11	Hap148	Id	34	108.9	China	Chang’an District, Xi’an City, Shaanxi	HM120761

E. argus barbouri	EA53_CA12	Hap149	Id	34	108.9	China	Chang'an District, Xi'an City, Shaanxi	HM120761
E. argus argus	Guo789	Hap46	Ia	34.61	114.26	China	Zhuxian Town, Kaifeng City, Henan	OR019330
E. argus argus	Guo794	Hap48	Ia	34.61	114.26	China	Zhuxian Town, Kaifeng City, Henan	OR019335
E. argus argus	Guo795	Hap49	Ia	34.61	114.26	China	Zhuxian Town, Kaifeng City, Henan	OR019336
E. argus argus	Guo792	Hap45	Ib	34.61	114.26	China	Zhuxian Town, Kaifeng City, Henan	OR019333
E. argus argus	Guo796	Hap50	Ib	34.61	114.26	China	Zhuxian Town, Kaifeng City, Henan	OR019337
E. argus argus	Guo788	Hap45	Ib	34.61	114.26	China	Zhuxian Town, Kaifeng City, Henan	OR019329
E. argus argus	Guo787	Hap42	If	34.61	114.26	China	Zhuxian Town, Kaifeng City, Henan	OR019328
E. argus argus	Guo793	Hap42	If	34.61	114.26	China	Zhuxian Town, Kaifeng City, Henan	OR019334
E. argus argus	Guo797	Hap42	If	34.61	114.26	China	Zhuxian Town, Kaifeng City, Henan	OR019338
E. argus barbouri	Guo800	Hap42	If	34.61	114.26	China	Zhuxian Town, Kaifeng City, Henan	OR019341
E. argus barbouri	Guo801	Hap42	If	34.61	114.26	China	Zhuxian Town, Kaifeng City, Henan	OR019342
E. argus barbouri	Guo799	Hap43	If	34.61	114.26	China	Zhuxian Town, Kaifeng City, Henan	OR019340
E. argus argus	Guo790	Hap47	If	34.61	114.26	China	Zhuxian Town, Kaifeng City, Henan	OR019332
E. argus barbouri	Guo798	Hap51	If	34.61	114.26	China	Zhuxian Town, Kaifeng City, Henan	OR019339
E. argus barbouri	Guo802	Hap52	If	34.61	114.26	China	Zhuxian Town, Kaifeng City, Henan	OR019343
E. argus argus	Guo804	Hap53	If	34.61	114.26	China	Zhuxian Town, Kaifeng City, Henan	OR019344
E. argus barbouri	Guo805	Hap54	If	34.61	114.26	China	Zhuxian Town, Kaifeng City, Henan	OR019345
E. argus barbouri	Guo806	Hap54	If	34.61	114.26	China	Zhuxian Town, Kaifeng City, Henan	OR019346
E. argus barbouri	Guo782E	Hap42	If	34.81	114.37	China	Henan University, Kaifeng City, Henan	OR019324
E. argus barbouri	Guo783	Hap43	If	34.72	115.2	China	Minquan Forest Farm, Shangqiu City , Henan	OR019325
E. argus argus	Guo785	Hap42	If	34.72	115.2	China	Minquan Forest Farm, Shangqiu City, Henan	OR019326
E. argus argus	Guo786	Hap44	Ia	34.72	115.2	China	Minquan Forest Farm, Shangqiu City, Henan	OR019327
E. argus barbouri	EA1_JY1	Hap101	Ia	35.1	112.6	China	Jiyuan City, Henan	HM120763
E. argus barbouri	EA1_JY2	Hap101	Ia	35.1	112.6	China	Jiyuan City, Henan	HM120763
E. argus barbouri	EA1_JY3	Hap101	Ia	35.1	112.6	China	Jiyuan City, Henan	HM120763
E. argus barbouri	EA1_JY4	Hap101	Ia	35.1	112.6	China	Jiyuan City, Henan	HM120763

E. argus barbouri	EA1_JY5	Hap101	Ia	35.1	112.6	China	Jiyuan City, Henan	HM120763
E. argus argus	EA13_JY6	Hap113	Ie	35.1	112.6	China	Jiyuan City, Henan	HM120771
E. argus argus	EA13_JY7	Hap113	Ie	35.1	112.6	China	Jiyuan City, Henan	HM120771
E. argus argus	EA14_JY8	Hap114	Ie	35.1	112.6	China	Jiyuan City, Henan	HM120772
E. argus argus	EA15_JY9	Hap115	IIb	35.1	112.6	China	Jiyuan City, Henan	HM120772
E. argus argus	EA15_JY10	Hap115	IIb	35.1	112.6	China	Jiyuan City, Henan	HM120773
E. argus argus	EA25_JY11	Hap125	Ic	35.1	112.6	China	Jiyuan City, Henan	HM120773
E. argus barbouri	Guo672	Hap41	Ia	35.97	104.17	China	Taipingpu village, Yuzhong County, Gansu	OR019313
E. argus barbouri	Guo2663	Hap277	Ia	36.1	103.79	China	Jiuzhoutai, Lanzhou City, Gansu	OR019281
E. argus barbouri	Guo2679	Hap278	Ia	36.1	103.79	China	Jiuzhoutai, Lanzhou City, Gansu	OR019282
E. argus argus	Guo2689	Hap277	Ia	36.1	103.79	China	Jiuzhoutai, Lanzhou City, Gansu	OR019285
E. argus barbouri	Guo2788	Hap277	Ia	36.1	103.79	China	Jiuzhoutai, Lanzhou City, Gansu	OR019284
E. argus argus	Guo2790	Hap277	Ia	36.1	103.79	China	Jiuzhoutai, Lanzhou City, Gansu	OR019286
E. argus barbouri	Guo1565	Hap76	Ia	36.1	103.79	China	Jiuzhoutai, Lanzhou City, Gansu	OR019208
E. argus barbouri	Guo1697	Hap280	Ia	36.1	103.79	China	Jiuzhoutai, Lanzhou City, Gansu	OR019230
E. argus barbouri	Guo1691	Hap282	Ia	36.1	103.79	China	Jiuzhoutai, Lanzhou City, Gansu	OR019228
E. argus barbouri	Guo1692	Hap71	Ia	36.1	103.79	China	Jiuzhoutai, Lanzhou City, Gansu	OR019229
E. argus barbouri	Guo1796	Hap279	Ia	36.1	103.79	China	Jiuzhoutai, Lanzhou City, Gansu	OR019231
E. argus barbouri	GXG2601	Hap274	Ia	36.12	111.07	China	Shiyao village, Xiangning County, Shanxi	OR019576
E. argus barbouri	GXG2602	Hap275	Ia	36.12	111.07	China	Shiyao village, Xiangning County, Shanxi	OR019577
E. argus barbouri	GXG2603	Hap276	Ia	36.12	111.07	China	Shiyao village, Xiangning County, Shanxi	OR019578
E. argus barbouri	Guo5170	Hap266	Ia	36.28	100.35	China	Shazhuyu village, Gonghe County, Qinghai	OR019298
E. argus barbouri	Guo5171	Hap267	Ia	36.28	100.35	China	Shazhuyu village, Gonghe County, Qinghai	OR019299
E. argus barbouri	Guo5173	Hap268	Ia	36.28	100.35	China	Shazhuyu village, Gonghe County, Qinghai	OR019301
E. argus barbouri	Guo5174	Hap269	Ia	36.28	100.35	China	Shazhuyu village, Gonghe County, Qinghai	OR019302
E. argus barbouri	Guo5178	Hap272	Ia	36.28	100.35	China	Shazhuyu village, Gonghe County, Qinghai	OR019306
E. argus barbouri	Guo5179	Hap273	Ia	36.28	100.35	China	Shazhuyu village, Gonghe County, Qinghai	OR019307

E. argus barbouri	Guo5181	Hap269	Ia	36.28	100.35	China	Shazhuyu village, Gonghe County, Qinghai	OR019309
E. argus barbouri	Guo5182	Hap273	Ia	36.28	100.35	China	Shazhuyu village, Gonghe County, Qinghai	OR019310
E. argus barbouri	GE0708047	Hap29	Ia	36.28	100.35	China	Shazhuyu village, Gonghe County, Qinghai	OR019147
E. argus barbouri	Guo5176	Hap270	Ic	36.28	100.35	China	Shazhuyu village, Gonghe County, Qinghai	OR019304
E. argus barbouri	Guo5177	Hap271	Ic	36.28	100.35	China	Shazhuyu village, Gonghe County, Qinghai	OR019305
E. argus barbouri	GE0708039	Hap28	If	36.28	100.35	China	Shazhuyu village, Gonghe County, Qinghai	OR019145
E. argus barbouri	GE0708046	Hap28	If	36.28	100.35	China	Shazhuyu village, Gonghe County, Qinghai	OR019146
E. argus barbouri	Guo5172	Hap28	If	36.28	100.35	China	Shazhuyu village, Gonghe County, Qinghai	OR019300
E. argus barbouri	Guo5175	Hap28	If	36.28	100.35	China	Shazhuyu village, Gonghe County, Qinghai	OR019303
E. argus barbouri	Guo5180	Hap28	If	36.28	100.35	China	Shazhuyu village, Gonghe County, Qinghai	OR019308
E. argus barbouri	EA29_GH1	Hap129	Ia	36.4	100.5	China	Gonghe County, Qinghai	HM120802
E. argus barbouri	EA30_GH2	Hap130	Ia	36.4	100.5	China	Gonghe County, Qinghai	HM120803
E. argus barbouri	EA31_GH3	Hap28	If	36.4	100.5	China	Gonghe County, Qinghai	HM120804
E. argus barbouri	EA32_GH4	Hap131	Ia	36.4	100.5	China	Gonghe County, Qinghai	HM120767
E. argus barbouri	EA32_GH5	Hap131	Ia	36.4	100.5	China	Gonghe County, Qinghai	HM120767
E. argus barbouri	EA32_GH6	Hap131	Ia	36.4	100.5	China	Gonghe County, Qinghai	HM120767
E. argus barbouri	EA32_GH7	Hap131	Ia	36.4	100.5	China	Gonghe County, Qinghai	HM120767
E. argus barbouri	EA32_GH8	Hap131	Ia	36.4	100.5	China	Gonghe County, Qinghai	HM120813
E. argus barbouri	EA32_GH9	Hap131	Ia	36.4	100.5	China	Gonghe County, Qinghai	HM120812
E. argus barbouri	EA32_GH10	Hap131	Ia	36.4	100.5	China	Gonghe County, Qinghai	HM120766
E. argus barbouri	EA32_GH11	Hap131	Ia	36.4	100.5	China	Gonghe County, Qinghai	HM120766
E. argus argus	MMS2628	Hap100	Ic	36.44	126.36	Korea	Unyeo Beach, Janggok-ri, Gonam-myeon, Taean-gun, Chungcheongnam-do	OR019603
E. argus argus	MMS2629	Hap100	Ic	36.44	126.36	Korea	Unyeo Beach, Janggok-ri, Gonam-myeon, Taean-gun, Chungcheongnam-do	OR019605
E. argus argus	MMS2630	Hap100	Ic	36.44	126.36	Korea	Unyeo Beach, Janggok-ri, Gonam-myeon, Taean-gun, Chungcheongnam-do	OR019606

E. argus argus	MMS2631	Hap100	Ic	36.44	126.36	Korea	Unyeo Beach, Janggok-ri, Gonam-myeon, Taean-gun, Chungcheongnam-do	OR019607
E. argus argus	MMS2632	Hap100	Ic	36.44	126.36	Korea	Unyeo Beach, Janggok-ri, Gonam-myeon, Taean-gun, Chungcheongnam-do	OR019608
E. argus argus	MMS2406	Hap100	Ic	36.64	126.3	Korea	Dalsanpo Beach, Dalsan- ri, Nam-myeon, Taean-gun, Chungcheongnam-do	OR019609
E. argus argus	MMS2407	Hap100	Ic	36.64	126.3	Korea	Dalsanpo Beach, Dalsan-ri, Nam-myeon, Taean-gun, Chungcheongnam-do	OR019610
E. argus argus	MMS2408	Hap100	Ic	36.64	126.3	Korea	Dalsanpo Beach, Dalsan-ri, Nam-myeon, Taean-gun, Chungcheongnam-do	OR019611
E. argus argus	MMS2409	Hap100	Ic	36.64	126.3	Korea	Dalsanpo Beach, Dalsan-ri, Nam-myeon, Taean-gun, Chungcheongnam-doa	OR019612
E. argus argus	MMS2410	Hap100	Ic	36.64	126.3	Korea	Dalsanpo Beach, Dalsan-ri, Nam-myeon, Taean-gun, Chungcheongnam-do	OR019604
E. argus barbouri	Guo2654	Hap84	Ie	36.53	102.02	China	Caojiapu village, Huzhu County, Qinghai	OR019280
E. argus argus	EA8_HD1	Hap108	IIb	36.6	114.5	China	Handan City, Hebei	HM120762
E. argus argus	EA9_HD2	Hap109	IIb	36.6	114.5	China	Handan City, Hebei	HM120762
E. argus argus	EA10_HD3	Hap110	IIb	36.6	114.5	China	Handan City, Hebei	HM120791
E. argus argus	EA11_HD4	Hap111	Ia	36.6	114.5	China	Handan City, Hebei	HM120791
E. argus argus	EA12_HD5	Hap112	Ia	36.6	114.5	China	Handan City, Hebei	HM120798
E. argus argus	EA22_HD6	Hap122	Id	36.6	114.5	China	Handan City, Hebei	HM120798
E. argus argus	EA24_HD7	Hap124	Ic	36.6	114.5	China	Handan City, Hebei	HM120798
E. argus argus	EA26_HD8	Hap126	Ia	36.6	114.5	China	Handan City, Hebei	HM120798
E. argus argus	EA26_HD9	Hap126	Ia	36.6	114.5	China	Handan City, Hebei	HM120798
E. argus argus	EA26_HD10	Hap126	Ia	36.6	114.5	China	Handan City, Hebei	HM120798
E. argus argus	EA26_HD11	Hap126	Ia	36.6	114.5	China	Handan City, Hebei	HM120799
E. argus argus	EA27_HD12	Hap127	Ia	36.6	114.5	China	Handan City, Hebei	HM120799

E. argus barbouri	Guo1592	Hap62	If	36.71	110.42	China	Nianpan village, Yanchuan County, Shaanxi	OR019211
E. argus barbouri	Guo1440	Hap63	Ic	36.71	110.42	China	Nianpan village, Yanchuan County, Shaanxi	OR019169
E. argus barbouri	Guo1442	Hap64	Ie	36.71	110.42	China	Nianpan village, Yanchuan County, Shaanxi	OR019170
E. argus barbouri	Guo1593	Hap77	Ie	36.71	110.42	China	Nianpan village, Yanchuan County, Shaanxi	OR019212
E. argus barbouri	Guo1594	Hap78	Ie	36.71	110.42	China	Nianpan village, Yanchuan County, Shaanxi	OR019213
E. argus barbouri	Guo1598	Hap79	Ie	36.71	110.42	China	Nianpan village, Yanchuan County, Shaanxi	OR019215
E. argus barbouri	Guo1599	Hap80	Ie	36.71	110.42	China	Nianpan village, Yanchuan County, Shaanxi	OR019216
E. argus barbouri	Guo1439	Hap62	If	36.71	110.42	China	Nianpan village, Yanchuan County, Shaanxi	OR019168
E. argus argus	SJS03	Hap160	IIb	36.84	121.7	China	Yintan district, Weihai City, Shandong	OR019601
E. argus argus	SJS04	Hap160	IIb	36.84	121.7	China	Yintan district, Weihai City, Shandong	OR019602
E. argus barbouri	Guo8895	Hap71	Ia	36.88	103.81	China	Dongjing village, Jingtai County, Gansu	OR019350
E. argus barbouri	Guo8896	Hap71	Ia	36.88	103.81	China	Dongjing village, Jingtai County, Gansu	OR019351
E. argus barbouri	Guo8897	Hap87	Ia	36.88	103.81	China	Dongjing village, Jingtai County, Gansu	OR019352
E. argus barbouri	Guo8898	Hap71	Ia	36.88	103.81	China	Dongjing village, Jingtai County, Gansu	OR019353
E. argus barbouri	Guo8899	Hap71	Ia	36.88	103.81	China	Dongjing village, Jingtai County, Gansu	OR019354
E. argus barbouri	Guo8900	Hap71	Ia	36.88	103.81	China	Dongjing village, Jingtai County, Gansu	OR019355
E. argus barbouri	Guo8901	Hap71	Ia	36.88	103.81	China	Dongjing village, Jingtai County, Gansu	OR019357
E. argus barbouri	Guo8902	Hap71	Ia	36.88	103.81	China	Dongjing village, Jingtai County, Gansu	OR019358
E. argus barbouri	Guo8955	Hap89	Ia	37.44	106.43	China	Tianyuan village, Hongsipu district, Wuzhong City, Ningxia	OR019361
E. argus barbouri	Guo8949	Hap88	Ic	37.45	106.17	China	Tianshuihe village, Hongsipu district, Wuzhong City, Ningxia	OR019360
E. argus argus	ZYCA006	Hap1	Ia	37.72	114.77	China	Dashiqiao village, Zhaoxian County, Hebei	OR019623
E. argus argus	ZYCA007	Hap2	Ib	37.72	114.77	China	Dashiqiao village, Zhaoxian County, Hebei	OR019624
E. argus argus	ZYCA008	Hap3	Ib	37.72	114.77	China	Dashiqiao village, Zhaoxian County, Hebei	OR019625
E.argus barbouri	EA18_YQ1	Hap118	Ia	37.9	113.6	China	Yangquan City, Shanxi	HM120800
E.argus barbouri	EA19_YQ2	Hap119	Ib	37.9	113.6	China	Yangquan City, Shanxi	HM120800
E.argus barbouri	EA21_YQ3	Hap121	Ia	37.9	113.6	China	Yangquan City, Shanxi	HM120800

E.argus barbouri	EA21_YQ4	Hap121	Ia	37.9	113.6	China	Yangquan City, Shanxi	HM120800
E.argus barbouri	EA21_YQ5	Hap121	Ia	37.9	113.6	China	Yangquan City, Shanxi	HM120801
E.argus barbouri	EA21_YQ6	Hap121	Ia	37.9	113.6	China	Yangquan City, Shanxi	HM120802
E.argus barbouri	EA35_YQ7	Hap134	Ia	37.9	113.6	China	Yangquan City, Shanxi	HM120802
E.argus barbouri	EA36_YQ8	Hap135	Ia	37.9	113.6	China	Yangquan City, Shanxi	HM120802
E.argus barbouri	EA37_YQ9	Hap136	Ic	37.9	113.6	China	Yangquan City, Shanxi	HM120802
E. argus barbouri	Guo1468	Hap68	If	38.17	109.78	China	Dahetan village, Yulin City, Shaanxi	OR019148
E. argus barbouri	Guo1606	Hap68	If	38.17	109.78	China	Dahetan village, Yulin City, Shaanxi	OR019219
E. argus barbouri	Guo1609	Hap71	Ia	38.17	109.78	China	Dahetan village, Yulin City, Shaanxi	OR019222
E. argus barbouri	Guo1610	Hap71	Ia	38.17	109.78	China	Dahetan village, Yulin City, Shaanxi	OR019223
E. argus barbouri	Guo1611	Hap74	Ia	38.17	109.78	China	Dahetan village, Yulin City, Shaanxi	OR019224
E. argus barbouri	Guo1612	Hap74	Ia	38.17	109.78	China	Dahetan village, Yulin City, Shaanxi	OR019225
E. argus barbouri	Guo1614	Hap71	Ia	38.17	109.78	China	Dahetan village, Yulin City, Shaanxi	OR019226
E. argus barbouri	Guo1448	Hap65	Ie	38.17	109.78	China	Dahetan village, Yulin City, Shaanxi	OR019171
E. argus barbouri	Guo1449	Hap66	Ie	38.17	109.78	China	Dahetan village, Yulin City, Shaanxi	OR019172
E. argus barbouri	Guo1451	Hap66	Ie	38.17	109.78	China	Dahetan village, Yulin City, Shaanxi	OR019173
E. argus barbouri	Guo1452	Hap65	Ie	38.17	109.78	China	Dahetan village, Yulin City, Shaanxi	OR019174
E. argus barbouri	Guo1453	Hap66	Ie	38.17	109.78	China	Dahetan village, Yulin City, Shaanxi	OR019175
E. argus barbouri	Guo1454	Hap66	Ie	38.17	109.78	China	Dahetan village, Yulin City, Shaanxi	OR019176
E. argus barbouri	Guo1460	Hap66	Ie	38.17	109.78	China	Dahetan village, Yulin City, Shaanxi	OR019178
E. argus barbouri	Guo1461	Hap66	Ie	38.17	109.78	China	Dahetan village, Yulin City, Shaanxi	OR019179
E. argus barbouri	Guo1471	Hap66	Ie	38.17	109.78	China	Dahetan village, Yulin City, Shaanxi	OR019180
E. argus barbouri	Guo1476	Hap66	Ie	38.17	109.78	China	Dahetan village, Yulin City, Shaanxi	OR019183
E. argus barbouri	Guo1477	Hap66	Ie	38.17	109.78	China	Dahetan village, Yulin City, Shaanxi	OR019184
E. argus barbouri	Guo1522	Hap66	Ie	38.17	109.78	China	Dahetan village, Yulin City, Shaanxi	OR019206
E. argus barbouri	Guo1571	Hap66	Ie	38.17	109.78	China	Dahetan village, Yulin City, Shaanxi	OR019210
E. argus barbouri	Guo1595	Hap66	Ie	38.17	109.78	China	Dahetan village, Yulin City, Shaanxi	OR019214

E. argus barbouri	Guo1607	Hap66	Ie	38.17	109.78	China	Dahetan village, Yulin City, Shaanxi	OR019220
E. argus barbouri	Guo1456	Hap67	If	38.17	109.78	China	Dahetan village, Yulin City, Shaanxi	OR019177
E. argus barbouri	Guo1463	Hap67	If	38.17	109.78	China	Dahetan village, Yulin City, Shaanxi	OR019626
E. argus barbouri	Guo1473	Hap69	If	38.17	109.78	China	Dahetan village, Yulin City, Shaanxi	OR019181
E. argus barbouri	Guo1475	Hap70	If	38.17	109.78	China	Dahetan village, Yulin City, Shaanxi	OR019182
E. argus barbouri	Guo1479	Hap67	If	38.17	109.78	China	Dahetan village, Yulin City, Shaanxi	OR019185
E. argus barbouri	Guo1570	Hap69	If	38.17	109.78	China	Dahetan village, Yulin City, Shaanxi	OR019209
E. argus barbouri	Guo1608	Hap69	If	38.17	109.78	China	Dahetan village, Yulin City, Shaanxi	OR019221
E. argus barbouri	Guo1682	Hap69	If	38.17	109.78	China	Dahetan village, Yulin City, Shaanxi	OR019227
E. argus barbouri	GXG2309	Hap224	Id	38.18	107.7	China	Hulagalaji, Otog Front Banner, Inner Mongolia	OR019541
E. argus barbouri	GXG2496	Hap234	Id	38.23	108.23	China	Taolimu, Otog Front Banner, Inner Mongolia	OR019558
E. argus barbouri	GXG2501	Hap235	Id	38.23	108.23	China	Taolimu, Otog Front Banner, Inner Mongolia	OR019559
E. argus barbouri	GXG2502	Hap236	Ia	38.23	108.23	China	Taolimu, Otog Front Banner, Inner Mongolia	OR019560
E. argus barbouri	GXG2323	Hap225	IIb	38.23	108.23	China	Taolimu, Otog Front Banner, Inner Mongolia	OR019543
E. argus barbouri	GXG2295	Hap221	Ia	38.89	107.71	China	Shahuchaidamu, Wushen Banner, Inner Mongolia	OR019538
E. argus barbouri	GXG2296	Hap222	Ia	38.89	107.71	China	Shahuchaidamu, Wushen Banner, Inner Mongolia	OR019539
E. argus barbouri	GXG2297	Hap223	Ia	38.89	107.71	China	Shahuchaidamu, Wushen Banner, Inner Mongolia	OR019540
E. argus barbouri	Guo893	Hap55	If	38.89	107.71	China	Shahuchaidamu, Wushen Banner, Inner Mongolia	OR019359
E. argus barbouri	GXG2324	Hap55	If	38.23	108.23	China	Taolimu, Otog Front Banner, Inner Mongolia	OR019544
E. argus barbouri	GXG2294	Hap55	If	38.89	107.71	China	Shahuchaidamu, Wushen Banner, Inner Mongolia	OR019537
E. argus barbouri	GXG2288	Hap220	Ia	39.1	107.27	China	Saiyinwusu, Otog Banner, Inner Mongolia	OR019536
E. argus barbouri	GXG2438	Hap233	Ia	39.1	107.27	China	Saiyinwusu, Otog Banner, Inner Mongolia	OR019557
E. argus barbouri	GXG2286	Hap55	If	39.1	107.27	China	Saiyinwusu, Otog Banner, Inner Mongolia	OR019535
E. argus barbouri	GXG2320	Hap55	If	39.1	107.27	China	Saiyinwusu, Otog Banner, Inner Mongolia	OR019542
E. argus barbouri	GXG2327	Hap221	Ia	39.27	108.04	China	Gekewusu, Otog Banner, Inner Mongolia	OR019546
E. argus barbouri	GXG2521	Hap238	Id	39.27	108.04	China	Gekewusu, Otog Banner, Inner Mongolia	OR019562
E. argus barbouri	GXG2523	Hap240	Id	39.27	108.04	China	Gekewusu, Otog Banner, Inner Mongolia	OR019564

E. argus barbouri	GXG2328	Hap55	If	39.27	108.04	China	Gekewusu, Otog Banner, Inner Mongolia	OR019545
E. argus barbouri	GXG2329	Hap226	IIb	39.27	108.04	China	Gekewusu, Otog Banner, Inner Mongolia	OR019547
E. argus barbouri	GXG2520	Hap237	IIb	39.27	108.04	China	Gekewusu, Otog Banner, Inner Mongolia	OR019561
E. argus barbouri	GXG2522	Hap239	IIb	39.27	108.04	China	Gekewusu, Otog Banner, Inner Mongolia	OR019563
E. argus barbouri	EA34_ETK1	Hap133	Ia	39.5	107.5	China	Otog Banner, Inner Mongolia	HM120767
E. argus barbouri	EA45_ETK2	Hap55	If	39.5	107.5	China	Otog Banner, Inner Mongolia	HM120767
E. argus barbouri	EA45_ETK3	Hap55	If	39.5	107.5	China	Otog Banner, Inner Mongolia	HM120767
E. argus barbouri	EA45_ETK4	Hap55	If	39.5	107.5	China	Otog Banner, Inner Mongolia	HM120767
E. argus barbouri	EA45_ETK5	Hap55	If	39.5	107.5	China	Otog Banner, Inner Mongolia	HM120767
E. argus barbouri	EA45_ETK6	Hap55	If	39.5	107.5	China	Otog Banner, Inner Mongolia	HM120767
E. argus barbouri	EA46_ETK7	Hap142	Id	39.5	107.5	China	Otog Banner, Inner Mongolia	HM120768
E. argus barbouri	EA47_ETK8	Hap143	Id	39.5	107.5	China	Otog Banner, Inner Mongolia	HM120768
E. argus barbouri	EA48_ETK9	Hap144	Id	39.5	107.5	China	Otog Banner, Inner Mongolia	HM120769
E. argus barbouri	EA49_ETK10	Hap145	Id	39.5	107.5	China	Otog Banner, Inner Mongolia	HM120769
E. argus barbouri	EA50_ETK11	Hap146	IIb	39.5	107.5	China	Otog Banner, Inner Mongolia	HM120770
E. argus barbouri	EA51_ETK12	Hap147	Id	39.5	107.5	China	Otog Banner, Inner Mongolia	HM120770
E. argus argus	SJS02_12	Hap151	Ia	39.56	122.17	China	Laomao Mountain, Pulandian City, Liaoning	OR019585
E. argus argus	SJS02_13	Hap151	Ia	39.56	122.17	China	Laomao Mountain, Pulandian City, Liaoning	OR019586
E. argus argus	SJS02_14	Hap151	Ia	39.56	122.17	China	Laomao Mountain, Pulandian City, Liaoning	OR019587
E. argus argus	SJS02_22	Hap151	Ia	39.56	122.17	China	Laomao Mountain, Pulandian City, Liaoning	OR019594
E. argus argus	SJS02_26	Hap151	Ia	39.56	122.17	China	Laomao Mountain, Pulandian City, Liaoning	OR019598
E. argus argus	SJS02_19	Hap155	Ia	39.56	122.17	China	Laomao Mountain, Pulandian City, Liaoning	OR019591
E. argus argus	SJS02_20	Hap156	Ia	39.56	122.17	China	Laomao Mountain, Pulandian City, Liaoning	OR019592
E. argus argus	SJS02_28	Hap159	Ia	39.56	122.17	China	Laomao Mountain, Pulandian City, Liaoning	OR019600
E. argus argus	SJS02_11	Hap150	Id	39.56	122.17	China	Laomao Mountain, Pulandian City, Liaoning	OR019584
E. argus argus	SJS02_23	Hap150	Id	39.56	122.17	China	Laomao Mountain, Pulandian City, Liaoning	OR019595
E. argus argus	SJS02_15	Hap152	If	39.56	122.17	China	Laomao Mountain, Pulandian City, Liaoning	OR019588

E. argus argus	SJS02_21	Hap157	If	39.56	122.17	China	Laomao Mountain, Pulandian City, Liaoning	OR019593
E. argus argus	SJS02_16	Hap153	IIb	39.56	122.17	China	Laomao Mountain, Pulandian City, Liaoning	OR019589
E. argus argus	SJS02_17	Hap154	IIb	39.56	122.17	China	Laomao Mountain, Pulandian City, Liaoning	OR019590
E. argus argus	SJS02_24	Hap154	IIb	39.56	122.17	China	Laomao Mountain, Pulandian City, Liaoning	OR019596
E. argus argus	SJS02_27	Hap154	IIb	39.56	122.17	China	Laomao Mountain, Pulandian City, Liaoning	OR019599
E. argus argus	SJS02_25	Hap158	IIb	39.56	122.17	China	Laomao Mountain, Pulandian City, Liaoning	OR019597
E. argus barbouri	GXG2334	Hap229	IIb	39.79	108.68	China	Chahanhushu, Hangjin Banner, Inner Mongolia	OR019550
E. argus barbouri	GXG2535	Hap24	Ia	39.79	108.68	China	Chahanhushu, Hangjin Banner, Inner Mongolia	OR019567
E. argus barbouri	GXG2333	Hap228	Id	39.79	108.68	China	Chahanhushu, Hangjin Banner, Inner Mongolia	OR019548
E. argus barbouri	GXG2533	Hap241	Id	39.79	108.68	China	Chahanhushu, Hangjin Banner, Inner Mongolia	OR019565
E. argus barbouri	GXG2332	Hap227	IIb	39.79	108.68	China	Chahanhushu, Hangjin Banner, Inner Mongolia	OR019549
E. argus barbouri	GXG2534	Hap242	IIb	39.79	108.68	China	Chahanhushu, Hangjin Banner, Inner Mongolia	OR019566
E. argus barbouri	GXG2553	Hap246	Ia	40.01	108.52	China	Tabitu, Hangjin Banner, Inner Mongolia	OR019573
E. argus barbouri	GXG2552	Hap216	Ib	40.01	108.52	China	Tabitu, Hangjin Banner, Inner Mongolia	OR019572
E. argus barbouri	Guo903	Hap8	Ib	40.01	108.52	China	Tabitu, Hangjin Banner, Inner Mongolia	OR019363
E. argus barbouri	GXG2341	Hap8	Ib	40.01	108.52	China	Tabitu, Hangjin Banner, Inner Mongolia	OR019551
E. argus barbouri	GXG2549	Hap8	Ib	40.01	108.52	China	Tabitu, Hangjin Banner, Inner Mongolia	OR019569
E. argus barbouri	GXG2546	Hap243	Id	40.01	108.52	China	Tabitu, Hangjin Banner, Inner Mongolia	OR019568
E. argus barbouri	GXG2551	Hap245	Id	40.01	108.52	China	Tabitu, Hangjin Banner, Inner Mongolia	OR019571
E. argus barbouri	GXG2550	Hap244	IIb	40.01	108.52	China	Tabitu, Hangjin Banner, Inner Mongolia	OR019570
E. argus barbouri	GXG2596	Hap247	Ia	40.05	110.85	China	Gongyigai village, Jungar Banner, Inner Mongolia	OR019574
E. argus barbouri	GXG2597	Hap247	Ia	40.05	110.85	China	Gongyigai village, Jungar Banner, Inner Mongolia	OR019575
E. argus argus	Guo9888	Hap95	Ia	40.41	116.31	China	Jiuduhe town, Huairou district, Beijing City	OR019373
E. argus argus	Guo9889	Hap96	Ia	40.41	116.31	China	Jiuduhe town, Huairou district, Beijing City	OR019374
E. argus argus	Guo9890	Hap95	Ia	40.41	116.31	China	Jiuduhe town, Huairou district, Beijing City	OR019375
E. argus argus	Guo9891	Hap95	Ia	40.41	116.31	China	Jiuduhe town, Huairou district, Beijing City	OR019376
E. argus argus	Guo9892	Hap97	If	40.41	116.31	China	Jiuduhe town, Huairou district, Beijing City	OR019377

E. argus barbouri	GXG1750	Hap161	IIb	40.58	111.81	China	Naomuqitai village, Helingeer County, Inner Mongolia	OR019381
E. argus argus	Guo9906	Hap98	If	40.66	116.19	China	Baihe fortress, Yanqing district, Beijing City	OR019378
E. argus argus	Guo9907	Hap99	If	40.66	116.19	China	Baihe fortress, Yanqing district, Beijing City	OR019379
E. argus argus	Guo9914	Hap98	If	40.66	116.2	China	Baihe Bridge 5, Yanqing district, Beijing City	OR019380
E. argus barbouri	Guo1306	Hap24	Ia	40.85	111.57	China	Wusutu National Forest Park, Hohhot City, Inner Mongolia	OR019166
E. argus argus	Guo1305	Hap8	Ib	40.85	111.57	China	Wusutu National Forest Park, Hohhot City, Inner Mongolia	OR019165
E. argus barbouri	Guo1308	Hap8	Ib	40.85	111.57	China	Wusutu National Forest Park, Hohhot City, Inner Mongolia	OR019167
E. argus barbouri	Guo372	Hap249	Ia	40.87	111.81	China	Wusutu National Forest Park, Hohhot City, Inner Mongolia	OR019294
E. argus barbouri	Guo376	Hap24	Ia	40.87	111.81	China	Wusutu National Forest Park, Hohhot City, Inner Mongolia	OR019296
E. argus barbouri	Guo371	Hap8	Ib	40.87	111.81	China	Wusutu National Forest Park, Hohhot City, Inner Mongolia	OR019293
E. argus barbouri	Guo375	Hap8	Ib	40.87	111.81	China	Wusutu National Forest Park, Hohhot City, Inner Mongolia	OR019295
E. argus barbouri	Guo370	Hap248	IIb	40.87	111.81	China	Wusutu National Forest Park, Hohhot City, Inner Mongolia	OR019292
E. argus barbouri	Guo378	Hap248	IIb	40.87	111.81	China	Wusutu National Forest Park, Hohhot City, Inner Mongolia	OR019297
E. argus barbouri	GXG2222	Hap71	Ia	41.04	107.62	China	Linhe district, Bayanaoer City, Inner Mongolia	OR019522
E. argus barbouri	EA16_XH1	Hap116	Id	41.1	113.9	China	Xinghe County, Ulanqab City, Inner Mongolia	HM120799
E. argus barbouri	EA16_XH2	Hap116	Id	41.1	113.9	China	Xinghe County, Ulanqab City, Inner Mongolia	HM120799
E. argus barbouri	EA16_XH3	Hap116	Id	41.1	113.9	China	Xinghe County, Ulanqab City, Inner Mongolia	HM120799
E. argus barbouri	EA16_XH4	Hap116	Id	41.1	113.9	China	Xinghe County, Ulanqab City, Inner Mongolia	HM120799
E. argus barbouri	EA16_XH5	Hap116	Id	41.1	113.9	China	Xinghe County, Ulanqab City, Inner Mongolia	HM120764
E. argus barbouri	EA16_XH6	Hap116	Id	41.1	113.9	China	Xinghe County, Ulanqab City, Inner Mongolia	HM120764
E. argus barbouri	EA16_XH7	Hap116	Id	41.1	113.9	China	Xinghe County, Ulanqab City, Inner Mongolia	HM120762
E. argus barbouri	EA16_XH8	Hap116	Id	41.1	113.9	China	Xinghe County, Ulanqab City, Inner Mongolia	HM120762
E. argus barbouri	EA16_XH9	Hap116	Id	41.1	113.9	China	Xinghe County, Ulanqab City, Inner Mongolia	HM120800
E. argus barbouri	EA17_XH10	Hap117	Id	41.1	113.9	China	Xinghe County, Ulanqab City, Inner Mongolia	HM120800
E. argus barbouri	EA20_XH11	Hap120	Ib	41.1	113.9	China	Xinghe County, Ulanqab City, Inner Mongolia	HM120800
E. argus barbouri	EA33_XH12	Hap132	Id	41.1	113.9	China	Xinghe County, Ulanqab City, Inner Mongolia	HM120800
E. argus barbouri	GXG2206	Hap57	Ie	41.11	107.87	China	Liangergedan, Wuyuan County, Inner Mongolia	OR019519

E. argus barbouri	GXG2207	Hap57	Ie	41.11	107.87	China	Liangergedan, Wuyuan County, Inner Mongolia	OR019520
E. argus barbouri	GXG2085	Hap211	Ia	41.34	108.5	China	Wind-eroded Ice Mortar Geopark, Urad Middle Banner, Inner Mongolia	OR019496
E. argus barbouri	GXG2086	Hap212	Ia	41.34	108.5	China	Wind-eroded Ice Mortar Geopark, Urad Middle Banner, Inner Mongolia	OR019497
E. argus barbouri	GXG2087	Hap211	Ia	41.34	108.5	China	Wind-eroded Ice Mortar Geopark, Urad Middle Banner, Inner Mongolia	OR019498
E. argus argus	Guo1187	Hap58	Ic	41.34	108.51	China	Laoyao, Urad Middle Banner, Inner Mongolia	OR019156
E. argus barbouri	GXG1989	Hap8	Ib	41.43	109.23	China	Houmaohuduge, Urad Middle Banner, Inner Mongolia	OR019476
E. argus barbouri	GXG1990	Hap8	Ib	41.43	109.23	China	Houmaohuduge, Urad Middle Banner, Inner Mongolia	OR019477
E. argus barbouri	GXG1992	Hap8	Ib	41.43	109.23	China	Houmaohuduge, Urad Middle Banner, Inner Mongolia	OR019479
E. argus barbouri	GXG1994	Hap8	Ib	41.43	109.23	China	Houmaohuduge, Urad Middle Banner, Inner Mongolia	OR019480
E. argus barbouri	GXG1991	Hap205	Ic	41.43	109.23	China	Houmaohuduge, Urad Middle Banner, Inner Mongolia	OR019478
E. argus barbouri	GXG1946	Hap193	Ia	41.44	110.84	China	Saiwusu village, Darhan-Muminggan Joint County, Inner Mongolia	OR019451
E. argus barbouri	GXG1947	Hap193	Ia	41.44	110.84	China	Saiwusu village, Darhan-Muminggan Joint County, Inner Mongolia	OR019452
E. argus barbouri	GXG2367	Hap231	Id	41.51	108.78	China	Bayinhaitaisumu, Urad Middle Banner, Inner Mongolia	OR019553
E. argus barbouri	GXG2359	Hap230	IIb	41.51	108.78	China	Bayinhaitaisumu, Urad Middle Banner, Inner Mongolia	OR019552
E. argus barbouri	GXG2368	Hap232	Ia	41.52	108.65	China	Tatuer, Urad Middle Banner, Inner Mongolia	OR019554
E. argus barbouri	GXG2371	Hap57	Ie	41.52	108.65	China	Tatuer, Urad Middle Banner, Inner Mongolia	OR019555
E. argus barbouri	GXG2373	Hap8	Ib	41.52	108.65	China	Tatuer, Urad Middle Banner, Inner Mongolia	OR019556
E. argus barbouri	GXG1758	Hap163	Ia	41.55	113.48	China	Houershuan village, Shangdu County, Inner Mongolia	OR019383
E. argus barbouri	GXG1759	Hap164	Ia	41.55	113.48	China	Houershuan village, Shangdu County, Inner Mongolia	OR019384
E. argus barbour	GXG1757	Hap162	IIb	41.55	113.48	China	Houershuan village, Shangdu County, Inner Mongolia	OR019382
E. argus argus	Guo1169	Hap56	Ia	41.61	108.52	China	Xiaertu, Urad Middle Banner, Inner Mongolia	OR019151
E. argus argus	Guo1170	Hap57	Ie	41.61	108.52	China	Xiaertu, Urad Middle Banner, Inner Mongolia	OR019153

E. argus barbouri	Guo1175	Hap8	Ib	41.69	108.41	China	Haoraotu, Urad Middle Banner, Inner Mongolia	OR019154
E. argus barbouri	ROM37590	Hap8	Ib	41.85	111.21	China	Wulanhua town, Siziwang Banner, Inner Mongolia	OR019583
E. argus barbouri	GXG1942	Hap8	Ib	42.02	110.16	China	Chagandele, Darhan-Muminggan Joint County, Inner Mongolia	OR019448
E. argus barbouri	ROM37439	Hap24	Ia	42.07	111.73	China	Chaganerige, Siziwang Banner, Inner Mongolia	OR019581
E. argus barbouri	GXG1944	Hap192	Ia	42.08	110.17	China	South of Bayinwulangacha, Darhan-Muminggan Joint County, Inner Mongolia	OR019450
E. argus barbouri	GXG2224	Hap216	Ib	42.08	110.17	China	South of Bayinwulangacha, Darhan-Muminggan Joint County, Inner Mongolia	OR019523
E. argus barbouri	GXG1943	Hap8	Ib	42.09	110.17	China	North of Bayinwulangacha, Darhan-Muminggan Joint County, Inner Mongolia	OR019449
E. argus barbouri	Guo1235	Hap24	Ia	42.26	110.51	China	NE Chaganhadasumu, Darhan-Muminggan Joint Banner, Inner Mongolia	OR019162
E. argus argus	Guo1234	Hap60	Ie	42.26	110.51	China	NE Chaganhadasumu, Darhan-Muminggan Joint Banner, Inner Mongolia	OR019161
E. argus barbouri	Guo1236	Hap30	If	42.26	110.51	China	NE Chaganhadasumu, Darhan-Muminggan Joint Banner, Inner Mongolia	OR019163
E. argus barbouri	GXG1954	Hap24	Ia	42.27	109.9	China	Bayinhua town, Darhan-Muminggan Joint Banner, Inner Mongolia	OR019455
E. argus barbouri	GXG1955	Hap194	Ia	42.27	109.9	China	Bayinhua town, Darhan-Muminggan Joint Banner, Inner Mongolia	OR019456
E. argus barbouri	GXG1956	Hap24	Ia	42.27	109.9	China	Bayinhua town, Darhan-Muminggan Joint Banner, Inner Mongolia	OR019457
E. argus barbouri	GXG2233	Hap24	Ia	42.27	109.9	China	Bayinhua town, Darhan-Muminggan Joint Banner, Inner Mongolia	OR019526
E. argus barbouri	GXG2237	Hap24	Ia	42.27	109.9	China	Bayinhua town, Darhan-Muminggan Joint Banner, Inner Mongolia	OR019528

E. argus barbouri	GXG2232	Hap217	Ib	42.27	109.9	China	Bayinhua town, Darhan-Muminggan Joint Banner, Inner Mongolia	OR019525
E. argus barbouri	GXG1953	Hap8	Ib	42.27	109.9	China	Bayinhua town, Darhan-Muminggan Joint Banner, Inner Mongolia	OR019454
E. argus barbouri	GXG2236	Hap8	Ib	42.27	109.9	China	Bayinhua town, Darhan-Muminggan Joint Banner, Inner Mongolia	OR019527
E. argus barbouri	GXG2238	Hap218	Id	42.27	109.9	China	Bayinhua town, Darhan-Muminggan Joint Banner, Inner Mongolia	OR019529
E. argus barbouri	GXG2239	Hap219	IIb	42.27	109.9	China	Bayinhua town, Darhan-Muminggan Joint Banner, Inner Mongolia	OR019530
E. argus barbouri	GXG2240	Hap219	IIb	42.27	109.9	China	Bayinhua town, Darhan-Muminggan Joint Banner, Inner Mongolia	OR019531
E. argus argus	GXG2178	Hap8	Ib	42.34	112.24	China	Gejige, Siziwang Banner, Inner Mongolia	OR019509
E. argus argus	GXG2187	Hap162	IIb	42.34	112.24	China	Gejige, Siziwang Banner, Inner Mongolia	OR019510
E. argus argus	GXG2188	Hap8	Ib	42.34	112.24	China	Gejige, Siziwang Banner, Inner Mongolia	OR019511
E. argus argus	GXG2189	Hap162	IIb	42.34	112.24	China	Gejige, Siziwang Banner, Inner Mongolia	OR019512
E. argus argus	GXG2192	Hap162	IIb	42.34	112.24	China	Gejige, Siziwang Banner, Inner Mongolia	OR019513
E. argus argus	GXG2193	Hap8	Ib	42.34	112.24	China	Gejige, Siziwang Banner, Inner Mongolia	OR019514
E. argus argus	GXG2194	Hap213	Ia	42.34	112.24	China	Gejige, Siziwang Banner, Inner Mongolia	OR019515
E. argus argus	GXG2195	Hap162	IIb	42.34	112.24	China	Gejige, Siziwang Banner, Inner Mongolia	OR019516
E. argus argus	GXG2196	Hap214	IIb	42.34	112.24	China	Gejige, Siziwang Banner, Inner Mongolia	OR019517
E. argus argus	GXG2197	Hap8	Ib	42.34	112.24	China	Gejige, Siziwang Banner, Inner Mongolia	OR019518
E. argus barbouri	GXG2242	Hap24	Ia	42.41	110.01	China	G210-118, Darhan-Muminggan Joint Banner, Inner Mongolia	OR019532
E. argus barbouri	GXG2257	Hap8	Ib	42.41	110.01	China	G210-118, Darhan-Muminggan Joint Banner, Inner Mongolia	OR019533
E. argus barbouri	GXG2258	Hap24	Ia	42.41	110.01	China	G210-118, Darhan-Muminggan Joint Banner, Inner	OR019534

							Mongolia	
E. argus barbouri	GXG2231	Hap187	Ia	42.47	110.44	China	Xiridele, Darhan-Muminggan Joint Banner, Inner Mongolia	OR019524
E. argus barbouri	GXG1952	Hap8	Ib	42.48	110.33	China	E'ru, Darhan-Muminggan Joint Banner, Inner Mongolia	OR019453
E. argus argus	GXG2158	Hap162	IIb	42.56	112.42	China	G209-184, Sonid Right Banner, Inner Mongolia	OR019507
E. argus argus	GXG2160	Hap162	IIb	42.56	112.42	China	G209-184, Sonid Right Banner, Inner Mongolia	OR019508
E. argus argus	Guo1222	Hap59	Id	42.65	115.58	China	Zhuha, Zhenglan Banner, Inner Mongolia	OR019159
E. argus barbouri	Guo1237	Hap61	Ic	42.65	115.58	China	Zhuha, Zhenglan Banner, Inner Mongolia	OR019164
E. argus argus	Guo1807	Hap250	Ia	42.77	122.47	China	Zhanggutai Town, Zhangwu County, Liaoning	OR019232
E. argus argus	Guo1808	Hap250	Ia	42.77	122.47	China	Zhanggutai Town, Zhangwu County, Liaoning	OR019233
E. argus argus	Guo1809	Hap250	Ia	42.77	122.47	China	Zhanggutai Town, Zhangwu County, Liaoning	OR019234
E. argus argus	Guo1810	Hap250	Ia	42.77	122.47	China	Zhanggutai Town, Zhangwu County, Liaoning	OR019235
E. argus argus	Guo1811	Hap251	Ia	42.77	122.47	China	Zhanggutai Town, Zhangwu County, Liaoning	OR019236
E. argus argus	Guo1812	Hap252	Ia	42.77	122.47	China	Zhanggutai Town, Zhangwu County, Liaoning	OR019237
E. argus argus	Guo1813	Hap253	Ia	42.77	122.47	China	Zhanggutai Town, Zhangwu County, Liaoning	OR019238
E. argus argus	Guo1814	Hap250	Ia	42.77	122.47	China	Zhanggutai Town, Zhangwu County, Liaoning	OR019239
E. argus argus	Guo1815	Hap250	Ia	42.77	122.47	China	Zhanggutai Town, Zhangwu County, Liaoning	OR019240
E. argus argus	Guo1817	Hap250	Ia	42.77	122.47	China	Zhanggutai Town, Zhangwu County, Liaoning	OR019242
E. argus argus	Guo1816	Hap252	Ia	42.77	122.47	China	Zhanggutai Town, Zhangwu County, Liaoning	OR019241
E. argus argus	Guo1818	Hap253	Ia	42.77	122.47	China	Zhanggutai Town, Zhangwu County, Liaoning	OR019243
E. argus argus	Guo1832	Hap250	Ia	42.77	122.47	China	Zhanggutai Town, Zhangwu County, Liaoning	OR019254
Eremias argus	Guo1833	Hap258	Ia	42.77	122.47	China	Zhanggutai Town, Zhangwu County, Liaoning	OR019255
E. argus argus	ROM37512	Hap25	Ia	42.82	112.67	China	Abrahamtara Town, Sonid Right Banner, Inner Mongolia	OR019582
E. argus argus	Guo1821	Hap254	Ia	42.82	122.38	China	Beidianzi village, Zhangwu County, Liaoning	OR019244
E. argus argus	Guo1822	Hap36	Ia	42.82	122.38	China	Beidianzi village, Zhangwu County, Liaoning	OR019245
E. argus argus	Guo1823	Hap255	If	42.82	122.38	China	Beidianzi village, Zhangwu County, Liaoning	OR019246
E. argus argus	Guo1824	Hap256	Ia	42.82	122.38	China	Beidianzi village, Zhangwu County, Liaoning	OR019247
E. argus argus	Guo1825	Hap256	Ia	42.82	122.38	China	Beidianzi village, Zhangwu County, Liaoning	OR019248

E. argus argus	Guo1826	Hap254	Ia	42.82	122.38	China	Beidianzi village, Zhangwu County, Liaoning	OR019249
E. argus argus	Guo1827	Hap254	Ia	42.82	122.38	China	Beidianzi village, Zhangwu County, Liaoning	OR019250
E. argus argus	Guo1828	Hap256	Ia	42.82	122.38	China	Beidianzi village, Zhangwu County, Liaoning	OR019251
E. argus argus	Guo1829	Hap257	Ia	42.82	122.38	China	Beidianzi village, Zhangwu County, Liaoning	OR019252
E. argus argus	Guo1830	Hap256	Ia	42.82	122.38	China	Beidianzi village, Zhangwu County, Liaoning	OR019253
E. argus argus	Guo1844	Hap259	Ia	42.86	123.2	China	Jinshatan, Erniu town, Kangping County, Liaoning	OR019256
E. argus argus	Guo1845	Hap260	Ia	42.86	123.2	China	Jinshatan, Erniu town, Kangping County, Liaoning	OR019257
E. argus argus	Guo1846	Hap259	Ia	42.86	123.2	China	Jinshatan, Erniu town, Kangping County, Liaoning	OR019258
E. argus argus	Guo1847	Hap261	Ia	42.86	123.2	China	Jinshatan, Erniu town, Kangping County, Liaoning	OR019259
E. argus argus	Guo1848	Hap261	Ia	42.86	123.2	China	Jinshatan, Erniu town, Kangping County, Liaoning	OR019260
E. argus argus	Guo1849	Hap262	Ia	42.86	123.2	China	Jinshatan, Erniu town, Kangping County, Liaoning	OR019261
E. argus argus	Guo1850	Hap263	Ia	42.86	123.2	China	Jinshatan, Erniu town, Kangping County, Liaoning	OR019262
E. argus argus	Guo1851	Hap261	Ia	42.86	123.2	China	Jinshatan, Erniu town, Kangping County, Liaoning	OR019263
E. argus argus	Guo1852	Hap260	Ia	42.86	123.2	China	Jinshatan, Erniu town, Kangping County, Liaoning	OR019264
E. argus argus	Guo1853	Hap181	Id	42.86	123.2	China	Jinshatan, Erniu town, Kangping County, Liaoning	OR019265
E. argus argus	Guo1854	Hap263	Ia	42.86	123.2	China	Jinshatan, Erniu town, Kangping County, Liaoning	OR019266
E. argus argus	Guo1855	Hap263	Ia	42.86	123.2	China	Jinshatan, Erniu town, Kangping County, Liaoning	OR019267
E. argus argus	Guo1856	Hap260	Ia	42.86	123.2	China	Jinshatan, Erniu town, Kangping County, Liaoning	OR019268
E. argus argus	Guo1857	Hap264	Ia	42.86	123.2	China	Jinshatan, Erniu town, Kangping County, Liaoning	OR019269
E. argus argus	GXG1885	Hap8	Ib	42.86	112.58	China	G208-108, Sonid Right Banner, Inner Mongolia	OR019441
E. argus argus	GXG1877	Hap162	IIb	42.86	112.58	China	G208-108, Sonid Right Banner, Inner Mongolia	OR019439
E. argus argus	GXG1878	Hap187	Ia	42.86	112.58	China	G208-108, Sonid Right Banner, Inner Mongolia	OR019440
E. argus argus	GXG1774	Hap167	Id	42.89	120.34	China	Changqin village, Aohan Banner, Inner Mongolia	OR019390
E. argus argus	GXG1775	Hap167	Id	42.89	120.34	China	Changqin village, Aohan Banner, Inner Mongolia	OR019391
E. argus argus	GXG1783	Hap168	Id	42.89	120.34	China	Changqin village, Aohan Banner, Inner Mongolia	OR019392
E. argus argus	GXG1784	Hap169	IIb	42.89	120.34	China	Changqin village, Aohan Banner, Inner Mongolia	OR019393
E. argus argus	GXG1786	Hap170	IIb	42.89	120.34	China	Changqin village, Aohan Banner, Inner Mongolia	OR019394

E. argus argus	GXG1787	Hap171	IIb	42.89	120.34	China	Changqin village, Aohan Banner, Inner Mongolia	OR019395
E. argus argus	GXG1788	Hap172	IIb	42.89	120.34	China	Changqin village, Aohan Banner, Inner Mongolia	OR019396
E. argus argus	GXG1791	Hap168	Id	42.89	120.34	China	Changqin village, Aohan Banner, Inner Mongolia	OR019397
E. argus argus	GXG1795	Hap168	Id	42.89	120.34	China	Changqin village, Aohan Banner, Inner Mongolia	OR019398
E. argus argus	GXG1797	Hap169	IIb	42.89	120.34	China	Changqin village, Aohan Banner, Inner Mongolia	OR019399
E. argus argus	GXG2208	Hap215	Id	43.03	112.81	China	G209-115, Sonid Right Banner, Inner Mongolia	OR019521
E. argus argus	GXG1768	Hap26	Ia	43.24	118.03	China	Heyan village, Keshiketeng Banner, Inner Mongolia	OR019385
E. argus argus	GXG1769	Hap165	Ia	43.24	118.03	China	Heyan village, Keshiketeng Banner, Inner Mongolia	OR019386
E. argus argus	GXG1770	Hap26	Ia	43.24	118.03	China	Heyan village, Keshiketeng Banner, Inner Mongolia	OR019387
E. argus argus	GXG1771	Hap166	Id	43.24	118.03	China	Heyan village, Keshiketeng Banner, Inner Mongolia	OR019388
E. argus argus	GXG1772	Hap26	Ia	43.24	118.03	China	Heyan village, Keshiketeng Banner, Inner Mongolia	OR019389
E. argus argus	GXG1799	Hap36	Ia	43.24	122.24	China	Bahutasumu, Kerqin Right Banner, Inner Mongolia	OR019400
E. argus argus	GXG1820	Hap175	IIa	43.24	122.24	China	Bahutasumu, Kerqin Right Banner, Inner Mongolia	OR019416
E. argus argus	GXG1821	Hap36	Ia	43.24	122.24	China	Bahutasumu, Kerqin Right Banner, Inner Mongolia	OR019417
E. argus argus	GXG1822	Hap175	IIa	43.24	122.24	China	Bahutasumu, Kerqin Right Banner, Inner Mongolia	OR019418
E. argus argus	GXG1824	Hap176	Ia	43.24	122.24	China	Bahutasumu, Kerqin Right Banner, Inner Mongolia	OR019419
E. argus argus	GXG1934	Hap189	Ia	43.33	115.69	China	Hongergaole town, Abaga Banner, Inner Mongolia	OR019445
E. argus argus	GXG1935	Hap190	Ia	43.33	115.69	China	Hongergaole town, Abaga Banner, Inner Mongolia	OR019446
E. argus argus	GXG1936	Hap191	Ib	43.33	115.69	China	Hongergaole town, Abaga Banner, Inner Mongolia	OR019447
E. argus argus	GXG1833	Hap177	Id	43.33	115.69	China	Hongergaole town, Abaga Banner, Inner Mongolia	OR019420
E. argus argus	GXG1836	Hap21	If	43.33	115.69	China	Hongergaole town, Abaga Banner, Inner Mongolia	OR019421
E. argus argus	GXG1839	Hap22	If	43.33	115.69	China	Hongergaole town, Abaga Banner, Inner Mongolia	OR019422
E. argus argus	GXG1933	Hap22	If	43.33	115.69	China	Hongergaole town, Abaga Banner, Inner Mongolia	OR019444
E. argus argus	GXG1876	Hap186	Ia	43.34	112.2	China	Wusutu, Erenhot City, Inner Mongolia	OR019438
E. argus argus	GXG1875	Hap8	Ib	43.34	112.2	China	Wusutu, Erenhot City, Inner Mongolia	OR019437
E. argus argus	GXG2101	Hap8	Ib	43.34	112.2	China	Wusutu, Erenhot City, Inner Mongolia	OR019500
E. argus argus	GXG2115	Hap8	Ib	43.34	112.2	China	Wusutu, Erenhot City, Inner Mongolia	OR019503

E. argus argus	GXG2118	Hap8	Ib	43.34	112.2	China	Wusutu, Erenhot City, Inner Mongolia	OR019506
E. argus argus	GXG2100	Hap162	IIb	43.34	112.2	China	Wusutu, Erenhot City, Inner Mongolia	OR019499
E. argus argus	GXG2113	Hap162	IIb	43.34	112.2	China	Wusutu, Erenhot City, Inner Mongolia	OR019501
E. argus argus	GXG2114	Hap162	IIb	43.34	112.2	China	Wusutu, Erenhot City, Inner Mongolia	OR019502
E. argus argus	GXG2116	Hap162	IIb	43.34	112.2	China	Wusutu, Erenhot City, Inner Mongolia	OR019504
E. argus argus	GXG2117	Hap162	IIb	43.34	112.2	China	Wusutu, Erenhot City, Inner Mongolia	OR019505
E. argus argus	GXG1909	Hap188	Ia	43.36	115.75	China	Hongergaole town, Abaga Banner, Inner Mongolia	OR019442
E. argus argus	WDC057	Hap20	Ia	43.36	115.75	China	Hongergaole town, Abaga Banner, Inner Mongolia	OR019613
E. argus argus	WDC058	Hap21	If	43.36	115.75	China	Hongergaole town, Abaga Banner, Inner Mongolia	OR019614
E. argus argus	WDC060	Hap22	If	43.36	115.75	China	Hongergaole town, Abaga Banner, Inner Mongolia	OR019615
E. argus argus	WDC061	Hap22	If	43.36	115.75	China	Hongergaole town, Abaga Banner, Inner Mongolia	OR019616
E. argus argus	WDC062	Hap8	Ib	43.36	115.75	China	Hongergaole town, Abaga Banner, Inner Mongolia	OR019617
E. argus argus	WDC063	Hap22	If	43.36	115.75	China	Hongergaole town, Abaga Banner, Inner Mongolia	OR019618
E. argus argus	WDC064	Hap23	Ia	43.36	115.75	China	Hongergaole town, Abaga Banner, Inner Mongolia	OR019619
E. argus argus	WDC065	Hap21	If	43.36	115.75	China	Hongergaole town, Abaga Banner, Inner Mongolia	OR019620
E. argus argus	WDC067	Hap22	If	43.36	115.75	China	Hongergaole town, Abaga Banner, Inner Mongolia	OR019621
E. argus argus	WDC082	Hap8	Ib	43.36	115.75	China	Hongergaole town, Abaga Banner, Inner Mongolia	OR019622
E. argus argus	GXG1918	Hap22	If	43.36	115.75	China	Hongergaole town, Abaga Banner, Inner Mongolia	OR019443
E. argus argus	GXG2055	Hap210	Ia	43.73	112.75	China	G331-6155, Sonid Left Banner, Inner Mongolia	OR019491
E. argus argus	GXG2056	Hap209	Ia	43.73	112.75	China	G331-6155, Sonid Left Banner, Inner Mongolia	OR019492
E. argus argus	GXG2058	Hap8	Ib	43.73	112.75	China	G331-6155, Sonid Left Banner, Inner Mongolia	OR019493
E. argus argus	GXG2060	Hap22	If	43.73	112.75	China	G331-6155, Sonid Left Banner, Inner Mongolia	OR019494
E. argus argus	GXG2062	Hap8	Ib	43.73	112.75	China	G331-6155, Sonid Left Banner, Inner Mongolia	OR019495
E. argus argus	GXG1862	Hap90	Ic	43.76	113.13	China	G331-6155, Sonid Left Banner, Inner Mongolia	OR019433
E. argus argus	GXG1863	Hap184	Ia	43.76	113.13	China	G331-6155, Sonid Left Banner, Inner Mongolia	OR019434
E. argus argus	GXG1864	Hap185	Ia	43.76	113.13	China	G331-6155, Sonid Left Banner, Inner Mongolia	OR019435
E. argus argus	GXG1868	Hap185	Ia	43.76	113.13	China	G331-6155, Sonid Left Banner, Inner Mongolia	OR019436

E. argus argus	GXG2032	Hap185	Ia	43.76	113.13	China	G331-6155, Sonid Left Banner, Inner Mongolia	OR019487
E. argus argus	GXG2036	Hap185	Ia	43.76	113.13	China	G331-6155, Sonid Left Banner, Inner Mongolia	OR019488
E. argus argus	GXG2037	Hap208	Ia	43.76	113.13	China	G331-6155, Sonid Left Banner, Inner Mongolia	OR019489
E. argus argus	GXG2042	Hap209	Ia	43.76	113.13	China	G331-6155, Sonid Left Banner, Inner Mongolia	OR019490
E. argus argus	Guo2504	Hap22	If	43.83	116.14	China	Shuliteamu, Xilinhot City, Inner Mongolia	OR019277
E. argus argus	Guo2505	Hap83	Ie	43.83	116.14	China	Shuliteamu, Xilinhot City, Inner Mongolia	OR019278
E. argus argus	GXG1852	Hap180	Ia	43.84	113.99	China	Tamugate, Abaga Banner, Inner Mongolia	OR019426
E. argus argus	GXG1854	Hap182	Ia	43.84	113.99	China	Tamugate, Abaga Banner, Inner Mongolia	OR019428
E. argus argus	GXG1858	Hap183	Ia	43.84	113.99	China	Tamugate, Abaga Banner, Inner Mongolia	OR019432
E. argus argus	GXG2028	Hap183	Ia	43.84	113.99	China	Tamugate, Abaga Banner, Inner Mongolia	OR019486
E. argus barbouri	KU331054	Hap19	Id	43.84	102.73	Mongolia	Sevrei, Omnogovi	OR019119
E. argus argus	GXG1853	Hap181	Id	43.84	113.99	China	Tamugate, Abaga Banner, Inner Mongolia	OR019427
E. argus argus	GXG1855	Hap22	If	43.84	113.99	China	Tamugate, Abaga Banner, Inner Mongolia	OR019429
E. argus argus	GXG1856	Hap8	Ib	43.84	113.99	China	Tamugate, Abaga Banner, Inner Mongolia	OR019430
E. argus argus	GXG1857	Hap8	Ib	43.84	113.99	China	Tamugate, Abaga Banner, Inner Mongolia	OR019431
E. argus argus	GXG2025	Hap8	Ib	43.84	113.99	China	Tamugate, Abaga Banner, Inner Mongolia	OR019484
E. argus argus	GXG2027	Hap22	If	43.84	113.99	China	Tamugate, Abaga Banner, Inner Mongolia	OR019485
E. argus argus	GXG1960	Hap195	Ia	43.93	115.81	China	Jirigelangtu, Xilinhot City, Inner Mongolia	OR019458
E. argus argus	GXG1961	Hap22	If	43.93	115.81	China	Jirigelangtu, Xilinhot City, Inner Mongolia	OR019459
E. argus argus	GXG1962	Hap22	If	43.95	114.72	China	Arenwusu, Abaga Banner, Inner Mongolia	OR019460
E. argus argus	GXG1965	Hap22	If	43.95	114.72	China	Arenwusu, Abaga Banner, Inner Mongolia	OR019461
E. argus argus	GXG1969	Hap22	If	43.93	115.81	China	Jirigelangtu, Xilinhot City, Inner Mongolia	OR019464
E. argus argus	GXG1970	Hap198	Ia	43.93	115.81	China	Jirigelangtu, Xilinhot City, Inner Mongolia	OR019465
E. argus argus	GXG1971	Hap22	If	43.93	115.81	China	Jirigelangtu, Xilinhot City, Inner Mongolia	OR019466
E. argus argus	GXG1972	Hap199	Ia	43.93	115.81	China	Jirigelangtu, Xilinhot City, Inner Mongolia	OR019467
E. argus argus	GXG1973	Hap200	Ic	43.93	115.81	China	Jirigelangtu, Xilinhot City, Inner Mongolia	OR019468
E. argus argus	GXG1974	Hap199	Ia	43.93	115.81	China	Jirigelangtu, Xilinhot City, Inner Mongolia	OR019469

E. argus argus	GXG1975	Hap201	Ia	43.93	115.81	China	Jirigelangtu, Xilinhot City, Inner Mongolia	OR019470
E. argus argus	GXG1979	Hap22	If	43.93	115.81	China	Jirigelangtu, Xilinhot City, Inner Mongolia	OR019471
E. argus argus	GXG1847	Hap178	Ia	43.95	114.72	China	Arenwusu, Abaga Banner, Inner Mongolia	OR019424
E. argus argus	GXG2000	Hap206	Ia	43.95	114.72	China	Arenwusu, Abaga Banner, Inner Mongolia	OR019481
E. argus argus	GXG2001	Hap206	Ia	43.95	114.72	China	Arenwusu, Abaga Banner, Inner Mongolia	OR019482
E. argus argus	GXG2002	Hap207	Ia	43.95	114.72	China	Arenwusu, Abaga Banner, Inner Mongolia	OR019483
E. argus argus	GXG1845	Hap22	If	43.95	114.72	China	Arenwusu, Abaga Banner, Inner Mongolia	OR019423
E. argus argus	GXG1801	Hap23	Ia	44.2	115.92	China	Talahutuge, Xilinhot City, Inner Mongolia	OR019401
E. argus argus	GXG1803	Hap23	Ia	44.2	115.92	China	Talahutuge, Xilinhot City, Inner Mongolia	OR019402
E. argus argus	GXG1804	Hap23	Ia	44.2	115.92	China	Talahutuge, Xilinhot City, Inner Mongolia	OR019403
E. argus argus	GXG1805	Hap173	IIb	44.2	115.92	China	Talahutuge, Xilinhot City, Inner Mongolia	OR019404
E. argus argus	GXG1806	Hap174	IIb	44.2	115.92	China	Talahutuge, Xilinhot City, Inner Mongolia	OR019405
E. argus argus	GXG1967	Hap196	Ia	44.52	114.23	China	Narenbulage, Abaga Banner, Inner Mongolia	OR019462
E. argus argus	GXG1968	Hap197	Ia	44.52	114.23	China	Narenbulage, Abaga Banner, Inner Mongolia	OR019463
E. argus argus	GXG1981	Hap202	Ia	44.52	114.23	China	Narenbulage, Abaga Banner, Inner Mongolia	OR019472
E. argus argus	GXG1982	Hap203	Ic	44.52	114.23	China	Narenbulage, Abaga Banner, Inner Mongolia	OR019473
E. argus argus	GXG1983	Hap204	Ic	44.52	114.23	China	Narenbulage, Abaga Banner, Inner Mongolia	OR019474
E. argus argus	GXG1984	Hap196	Ia	44.52	114.23	China	Narenbulage, Abaga Banner, Inner Mongolia	OR019475
E. argus argus	GXG1848	Hap179	Id	44.52	114.23	China	Narenbulage, Abaga Banner, Inner Mongolia	OR019425
E. argus argus	GXG1807	Hap23	Ia	44.92	116.12	China	East Ujimqin Banner, Inner Mongolia	OR019406
E. argus argus	GXG1808	Hap23	Ia	44.92	116.12	China	Saiyinwusugacha, East Ujimqin Banner, Inner Mongolia	OR019407
E. argus argus	GXG1809	Hap23	Ia	44.92	116.12	China	East Ujimqin Banner, Inner Mongolia	OR019408
E. argus argus	GXG1810	Hap23	Ia	44.92	116.12	China	East Ujimqin Banner, Inner Mongolia	OR019409
E. argus argus	GXG1811	Hap23	Ia	44.92	116.12	China	East Ujimqin Banner, Inner Mongolia	OR019410
E. argus argus	GXG1812	Hap23	Ia	44.92	116.12	China	East Ujimqin Banner, Inner Mongolia	OR019411
E. argus argus	GXG1813	Hap23	Ia	44.92	116.12	China	East Ujimqin Banner, Inner Mongolia	OR019412
E. argus argus	GXG1814	Hap23	Ia	44.92	116.12	China	East Ujimqin Banner, Inner Mongolia	OR019413

E. argus argus	GXG1815	Hap23	Ia	44.92	116.12	China	East Ujimqin Banner, Inner Mongolia	OR019414
E. argus argus	GXG1816	Hap23	Ia	44.92	116.12	China	East Ujimqin Banner, Inner Mongolia	OR019415
E. argus barbouri	Guo115	Hap36	Ia	45.58	126.22	China	Harbin Taiping International Airport, Heilongjiang	OR019149
E. argus argus	Guo116	Hap37	If	45.58	126.22	China	Harbin Taiping International Airport, Heilongjiang	OR019150
E. argus barbouri	Guo117	Hap38	Ia	45.58	126.22	China	Harbin Taiping International Airport, Heilongjiang	OR019152
E. argus barbouri	Guo118	Hap36	Ia	45.58	126.22	China	Harbin Taiping International Airport, Heilongjiang	OR019155
E. argus barbouri	Guo119	Hap39	Ia	46.91	124.21	China	Keertai village, Dorbod County, Heilongjiang	OR019157
E. argus argus	Guo120	Hap39	Ia	46.91	124.21	China	Keertai village, Dorbod County, Heilongjiang	OR019158
E. argus argus	Guo123	Hap40	Ie	46.91	124.21	China	Keertai village, Dorbod County, Heilongjiang	OR019160
E. argus barbouri	EA7_HRB1	Hap107	IIb	45.7	126.6	China	Harbin City, Heilongjiang	HM120761
E. argus barbouri	EA28_HRB2	Hap128	Ia	45.7	126.6	China	Harbin City, Heilongjiang	HM120783
E. argus barbouri	EA32_HRB3	Hap131	Ia	45.7	126.6	China	Harbin City, Heilongjiang	HM120783
E. argus barbouri	EA38_HRB4	Hap137	Ia	45.7	126.6	China	Harbin City, Heilongjiang	HM120783
E. argus barbouri	EA38_HRB5	Hap137	Ia	45.7	126.6	China	Harbin City, Heilongjiang	HM120783
E. argus barbouri	EA38_HRB6	Hap137	Ia	45.7	126.6	China	Harbin City, Heilongjiang	HM120784
E. argus barbouri	EA39_HRB7	Hap37	If	45.7	126.6	China	Harbin City, Heilongjiang	HM120786
E. argus barbouri	EA39_HRB8	Hap37	If	45.7	126.6	China	Harbin City, Heilongjiang	HM120786
E. argus barbouri	EA39_HRB9	Hap37	If	45.7	126.6	China	Harbin City, Heilongjiang	HM120786
E. argus barbouri	EA40_HRB10	Hap36	Ia	45.7	126.6	China	Harbin City, Heilongjiang	HM120786
E. argus barbouri	EA40_HRB11	Hap36	Ia	45.7	126.6	China	Harbin City, Heilongjiang	HM120787
E. argus barbouri	EA40_HRB12	Hap36	Ia	45.7	126.6	China	Harbin City, Heilongjiang	HM120787
E. argus barbouri	EA40_HRB13	Hap36	Ia	45.7	126.6	China	Harbin City, Heilongjiang	HM120788
E. argus barbouri	EA41_HRB14	Hap138	Id	45.7	126.6	China	Harbin City, Heilongjiang	HM120788
E. argus barbouri	Guo2919	Hap265	Ia	45.73	106.15	Mongolia	Saintsagaan, Dundgovi	OR019291
E. argus barbouri	Guo2908	Hap85	Ie	47.63	118.35	Mongolia	Khalkhgol, Dornod	OR019289
E. argus barbouri	Guo2909	Hap86	Ic	47.63	118.35	Mongolia	Khalkhgol, Dornod	OR019290
E. argus barbouri	CAS 238656	Hap4	Ib	47.78	104.39	Mongolia	6.5 air km SW of Bayannuur, Dashinchilen, Bulgan	OR019120

E. argus barbouri	CAS 238657	Hap5	Ia	47.78	104.39	Mongolia	6.5 air km SW of Bayannuur, Dashinchilen, Bulgan	OR019121
E. argus barbouri	CAS 238658	Hap6	Ib	47.78	104.39	Mongolia	6.5 air km SW of Bayannuur, Dashinchilen, Bulgan	OR019122
E. argus barbouri	CAS 238659	Hap7	Ib	47.78	104.39	Mongolia	6.5 air km SW of Bayannuur, Dashinchilen, Bulgan	OR019123
E. argus barbouri	CAS 238660	Hap8	Ib	47.78	104.39	Mongolia	6.5 air km SW of Bayannuur, Dashinchilen, Bulgan	OR019124
E. argus barbouri	CAS 238661	Hap9	Ia	47.78	104.39	Mongolia	6.5 air km SW of Bayannuur, Dashinchilen, Bulgan	OR019125
E. argus barbouri	CAS 238663	Hap10	Ib	47.78	104.39	Mongolia	6.5 air km SW of Bayannuur, Dashinchilen, Bulgan	OR019126
E. argus barbouri	CAS 238664	Hap8	Ib	47.78	104.39	Mongolia	6.5 air km SW of Bayannuur, Dashinchilen, Bulgan	OR019127
E. argus barbouri	CAS 238665	Hap11	IIb	47.78	104.39	Mongolia	6.5 air km SW of Bayannuur, Dashinchilen, Bulgan	OR019128
E. argus barbouri	CAS 238666	Hap12	IIb	47.78	104.39	Mongolia	6.5 air km SW of Bayannuur, Dashinchilen, Bulgan	OR019129
E. argus barbouri	CAS 238667	Hap8	Ib	47.78	104.39	Mongolia	6.5 air km SW of Bayannuur, Dashinchilen, Bulgan	OR019130
E. argus barbouri	CAS 238668	Hap13	IIb	47.78	104.39	Mongolia	6.5 air km SW of Bayannuur, Dashinchilen, Bulgan	OR019131
E. argus barbouri	CAS 238669	Hap8	Ib	47.78	104.39	Mongolia	6.5 air km SW of Bayannuur, Dashinchilen, Bulgan	OR019132
E. argus barbouri	CAS 238670	Hap4	Ib	47.78	104.39	Mongolia	6.5 air km SW of Bayannuur, Dashinchilen, Bulgan	OR019133
E. argus barbouri	CAS 238671	Hap8	Ib	47.78	104.39	Mongolia	6.5 air km SW of Bayannuur, Dashinchilen, Bulgan	OR019134
E. argus barbouri	CAS 238672	Hap14	IIb	47.65	104.36	Mongolia	23 air km SW of Bayannuur, Dashinchilen, Bulgan	OR019135
E. argus barbouri	CAS 238673	Hap8	Ib	47.65	104.36	Mongolia	23 air km SW of Bayannuur, Dashinchilen, Bulgan	OR019136
E. argus barbouri	CAS 238674	Hap8	Ib	47.65	104.36	Mongolia	23 air km SW of Bayannuur, Dashinchilen, Bulgan	OR019137
E. argus barbouri	CAS 238675	Hap15	IIb	47.65	104.36	Mongolia	23 air km SW of Bayannuur, Dashinchilen, Bulgan	OR019138
E. argus barbouri	CAS 238676	Hap8	Ib	47.65	104.36	Mongolia	23 air km SW of Bayannuur, Dashinchilen, Bulgan	OR019139
E. argus barbouri	CAS 238677	Hap16	IIb	47.65	104.36	Mongolia	23 air km SW of Bayannuur, Dashinchilen, Bulgan	OR019140
E. argus barbouri	CAS 238678	Hap8	Ib	47.65	104.36	Mongolia	23 air km SW of Bayannuur, Dashinchilen, Bulgan	OR019141
E. argus barbouri	CAS 238679	Hap17	IIb	47.65	104.36	Mongolia	23 air km SW of Bayannuur, Dashinchilen, Bulgan	OR019142
E. argus barbouri	CAS 238680	Hap18	Id	47.65	104.36	Mongolia	23 air km SW of Bayannuur, Dashinchilen, Bulgan	OR019143
E. argus barbouri	CAS 238681	Hap8	Ib	47.65	104.36	Mongolia	23 air km SW of Bayannuur, Dashinchilen, Bulgan	OR019144
E. argus argus	NM08015	Hap26	Ia	48.29	117.38	China	Ulan Nuoer Protection Station, New Barag West County, Inner Mongolia	OR019580

E. argus argus	N0011	Hap27	Ia	48.59	117.27	China	Galada Baixin Protection Station, New Barag West County, Inner Mongolia	OR019579
E. argus barbouri	Guo9707	Hap90	Ic	50.24	106.18	Mongolia	Atlanbulag, Selenge	OR019365
E. argus barbouri	Guo9708	Hap91	Ia	50.24	106.18	Mongolia	Atlanbulag, Selenge	OR019366
E. argus barbouri	Guo9709	Hap92	Ia	50.24	106.18	Mongolia	Atlanbulag, Selenge	OR019367
E. argus barbouri	Guo9710	Hap8	Ib	50.24	106.18	Mongolia	Atlanbulag, Selenge	OR019368
E. argus barbouri	Guo9711	Hap8	Ib	50.24	106.18	Mongolia	Atlanbulag, Selenge	OR019369
E. argus barbouri	Guo9712	Hap8	Ib	50.24	106.18	Mongolia	Atlanbulag, Selenge	OR019370
E. argus barbouri	Guo9714	Hap93	Ia	50.24	106.18	Mongolia	Atlanbulag, Selenge	OR019371
E. argus barbouri	Guo9715	Hap94	If	50.24	106.18	Mongolia	Atlanbulag, Selenge	OR019372
E. argus argus	Guo21	Hap30	If	51.73	107.46	Russia	Ivolginsky District, Buryatia Republic	OR019272
E. argus argus	Guo22	Hap30	If	51.73	107.46	Russia	Ivolginsky District, Buryatia Republic,	OR019273
E. argus argus	Guo23	Hap8	Ib	51.73	107.46	Russia	Ivolginsky District, Buryatia Republic,	OR019274
E. argus argus	Guo24	Hap30	If	51.73	107.46	Russia	Ivolginsky District, Buryatia Republic,	OR019275
E. argus argus	Guo25	Hap30	If	51.55	107.36	Russia	Tarbagataysky District, Buryatia Republic	OR019276
E. argus barbouri	Guo26	Hap31	If	51.55	107.36	Russia	Tarbagataysky District, Buryatia Republic	OR019279
E. argus barbouri	Guo27	Hap30	If	51.55	107.36	Russia	Tarbagataysky District, Buryatia Republic	OR019283
E. argus argus	Guo28	Hap8	Ib	51.55	107.36	Russia	Tarbagataysky District, Buryatia Republic	OR019287
E. argus argus	Guo29	Hap8	Ib	51.55	107.36	Russia	Tarbagataysky District, Buryatia Republic	OR019288
E. argus argus	Guo66	Hap30	If	51.76	107.54	Russia	Tarbagataysky District, Buryatia Republic	OR019311
E. argus argus	Guo67	Hap32	Ie	51.76	107.54	Russia	Tarbagataysky District, Buryatia Republic	OR019312
E. argus argus	Guo68	Hap30	If	51.76	107.54	Russia	Tarbagataysky District, Buryatia Republic	OR019314
E. argus argus	Guo69	Hap30	If	51.76	107.54	Russia	Tarbagataysky District, Buryatia Republic	OR019315
E. argus argus	Guo70	Hap30	If	51.76	107.54	Russia	Tarbagataysky District, Buryatia Republic	OR019316
E. argus argus	Guo71	Hap30	If	51.76	107.54	Russia	Tarbagataysky District, Buryatia Republic	OR019317
E. argus argus	Guo72	Hap33	Ie	51.76	107.54	Russia	Tarbagataysky District, Buryatia Republic	OR019318
E. argus argus	Guo73	Hap30	If	51.76	107.54	Russia	Tarbagataysky District, Buryatia Republic	OR019319

E. argus argus	Guo74	Hap30	If	51.76	107.54	Russia	Tarbagataysky District, Buryatia Republic	OR019320
E. argus argus	Guo75	Hap30	If	51.76	107.54	Russia	Tarbagataysky District, Buryatia Republic	OR019321
E. argus argus	Guo79	Hap34	Ie	51.76	107.54	Russia	Tarbagataysky District, Buryatia Republic	OR019331
E. argus argus	Guo76	Hap30	If	51.76	107.54	Russia	Tarbagataysky District, Buryatia Republic	OR019322
E. argus argus	Guo77	Hap8	Ib	51.76	107.54	Russia	Tarbagataysky District, Buryatia Republic	OR019323
E. argus barbouri	Guo84	Hap8	Ib	51.76	107.54	Russia	Tarbagataysky District, Buryatia Republic	OR019347
E. argus barbouri	Guo87	Hap8	Ib	51.76	107.54	Russia	Tarbagataysky District, Buryatia Republic	OR019348
E. argus barbouri	Guo88	Hap35	Ia	51.76	107.54	Russia	Tarbagataysky District, Buryatia Republic	OR019349
E. argus argus	Guo89	Hap8	Ib	51.76	107.54	Russia	Tarbagataysky District, Buryatia Republic	OR019356
E. argus argus	Guo90	Hap30	If	51.76	107.54	Russia	Tarbagataysky District, Buryatia Republic	OR019362
E. argus argus	Guo91	Hap30	If	51.76	107.54	Russia	Tarbagataysky District, Buryatia Republic	OR019364

Sequences of GenBank accession numbers HM120761–HM120800 are from Zhao et al. [32], and their subspecies identity is assigned according to Zhao et al. [26].

References

26. Zhao, E.M.; Zhao, K.T.; Zhou, K.Y. *Fauna Sinica, Reptilia (Squamata: Lacertilia)*; Volume 2; Science Press: Beijing, China, 1999. (In Chinese)

32. Zhao, Q.; Liu, H.X.; Luo, L.G.; Ji, X. Comparative population genetics and phylogeography of two lacertid lizards (*Eremias argus* and *E. brenchleyi*) from China. *Mol. Phylogenet. Evol.* **2011**, *58*, 478–491. https://doi.org/10.1016/j.ympev.2010.12.017.

Table S2 Data on the phylogenetic, molecular dating, Bayesian phylogeographic diffusion and BSP analyses, including partitions, models and parameters.

Analysis	Dataset	Beast Model	Partition identity	Length (bp)	Clock / Tree model
Phylogenetic tree (BI) analysis of the haplotypes of *E. argus*	282 haplotypes of *E. argus*, and three outgroups	GTR+G	cyt *b* 1st codon	423	
			cyt *b* 2nd codon	423	
			cyt *b* 3rd codon	423	
Phylogenetic tree (ML) analysis of the haplotypes of *E. argus*	282 haplotypes of *E. argus*, and three outgroups	GTR+G	cyt *b* 1st codon	423	
			cyt *b* 2nd codon	423	
			cyt *b* 3rd codon	423	
Molecular dating with calibration points of Lacertidae	*Cytb* and 12S rRNA of 142 species involved 3 individuals of *E. argus*	GTR+I+G	cyt *b* 1st codon	1200	Relaxed Uncorrelated Lognormal Yule Random starting tree
		GTR+I+G	cyt *b* 2nd codon	1200	
		GTR+I+G	cyt *b* 3rd codon	1200	
		GTR+I+G	12S rRNA	1049	
Molecular dating of haplotypes within *E. argus*	282 haplotypes of *E. argus*	GTR+G	cyt *b* 1st codon	423	Relaxed Uncorrelated Lognormal/ Constant-size Coalescence Random starting tree
		GTR+G	cyt *b* 2nd codon	423	
		GTR+G	cyt *b* 3rd codon	423	
Molecular dating of sequence within *E. argus*	614 sequences of *E. argus*	GTR+I+G	cyt *b* 1st codon	1041	Relaxed Uncorrelated Lognormal/ Constant-size Coalescence Random starting tree
		GTR+I+G	cyt *b* 2nd codon	1041	
		GTR+G	cyt *b* 3rd codon	1041	
BSP of Clade Ia	All sequences of Clade Ia	GTR+I+G	cyt *b* 1st codon	1041	Strict clock Coalescent: Bayesian Skyline Plot
		GTR+I+G	cyt *b* 2nd codon	1041	
		GTR+G	cyt *b* 3rd codon	1041	

					Random starting tree<br>Piecewise constant
BSP of Clade Ib	All sequences of Clade Ib	GTR<br>GTR<br>GTR	cyt *b* 1st codon<br>cyt *b* 2nd codon<br>cyt *b* 3rd codon	1041<br>1041<br>1041	Strict clock<br>Coalescent: Bayesian Skyline Plot<br>Random starting tree<br>Piecewise liner
BSP of Clade Ic	All sequences of Clade Ic	GTR+G<br>GTR+G<br>GTR	cyt *b* 1st codon<br>cyt *b* 2nd codon<br>cyt *b* 3rd codon	1041<br>1041<br>1041	Coalescent: Bayesian Skyline Plot<br>Random starting tree<br>Piecewise constant
BSP of Clade Id	All sequences of Clade Id	GTR+G<br>GTR+G<br>GTR+G	cyt *b* 1st codon<br>cyt *b* 2nd codon<br>cyt *b* 3rd codon	1041<br>1041<br>1041	Coalescent: Bayesian Skyline Plot<br>Random starting tree<br>Piecewise constant
BSP of Clade Ie	All sequences of Clade Ie	GTR+G<br>GTR+G<br>GTR+G	cyt *b* 1st codon<br>cyt *b* 2nd codon<br>cyt *b* 3rd codon	1041<br>1041<br>1041	Coalescent: Bayesian Skyline Plot<br>Random starting tree<br>Piecewise constant
BSP of Clade If	All sequences of Clade If	GTR+G<br>GTR+G<br>GTR+G	cyt *b* 1st codon<br>cyt *b* 2nd codon<br>cyt *b* 3rd codon	1041<br>1041<br>1041	Coalescent: Bayesian Skyline Plot<br>Random starting tree<br>Piecewise constant
BSP of Clade IIa	All sequences of Clade IIa	GTR+I+G<br>GTR+I+G	cyt *b* 1st codon<br>cyt *b* 2nd codon	1041<br>1041	Coalescent: Bayesian Skyline Plot

		GTR+G	cyt *b* 3rd codon	1041	Random starting tree
					Piecewise constant
Phylogeographic diffusion analysis of *E. argus*	Sequences of different locations	GTR+G	cyt *b* 1st codon	1041	Gamma relaxed random walk model
		GTR+G	cyt *b* 2nd codon	1041	
		GTR+G	cyt *b* 3rd codon	1041	

Table S3 Sequences of lacertid species (Lacertidae) and three outgroups retrieved form GenBank

Taxon	Family	Subfamily	GenBank accession number		Reference
			cyt *b*	12S rRNA	
Acanthodactylus aureus	Lacertidae	Eremiainae	MW496121	MW496121	[110]
Acanthodactylus boskianus	Lacertidae	Eremiainae	MW496112	MW496112	[110]
Acanthodactylus erythrurus	Lacertidae	Eremiainae	MW496113	MW496113	[110]
Acanthodactylus guineensis	Lacertidae	Eremiainae	MW496123	MW496123	[110]
Acanthodactylus schmidti	Lacertidae	Eremiainae	MW496124	MW496124	[110]
Adolfus jacksoni	Lacertidae	Eremiainae	AF206539	AF080387	[111]; [112]
Algyroides fitzingeri	Lacertidae	Lacertinae	MN015168	AF206598	[113]; [111]
Algyroides marchi	Lacertidae	Lacertinae	GQ142133	GQ142080	[49]
Algyroides moreoticus	Lacertidae	Lacertinae	GQ142131	GQ142079	[49]
Algyroides nigropunctatus	Lacertidae	Lacertinae	MW496122	MW496122	[110]
Amphisbaena schmidti	Amphisbaenidae	N/A	AY605475	AY605475	[114]
Anatololacerta anatolica	Lacertidae	Lacertinae	GQ142138	GQ142086	[49]
Apathya cappadocica	Lacertidae	Lacertinae	MN015129	GQ142076	[113]; [49]
Archaeolacerta bedriagae	Lacertidae	Lacertinae	MN015130	AF440599	[113]; [115]
Atlantolacerta andreanskyi	Lacertidae	Eremiainae	MN015162	GQ142070	[113]; [49]
Australolacerta australis	Lacertidae	Eremiainae	MW496118	MW496118	[110]
Bipes biporus	Bipedidae	N/A	AY605481	AY605481	[114]
Dalmatolacerta oxycephala	Lacertidae	Lacertinae	MN015132	KX080609	[113]; [116]
Darevskia armeniaca	Lacertidae	Lacertinae	MG704915	MG704915	[117]
Darevskia brauneri	Lacertidae	Lacertinae	MH481137	MH481137	[117]
Darevskia caucasica	Lacertidae	Lacertinae	MH481131	MH481131	[117]
Darevskia chlorogaster	Lacertidae	Lacertinae	MH481136	MH481136	[117]
Darevskia clarkorum	Lacertidae	Lacertinae	MH481134	MH481134	[117]
Darevskia daghestanica	Lacertidae	Lacertinae	MG704916	MG704916	[117]
Darevskia dahli	Lacertidae	Lacertinae	MH481135	MH481135	[117]
Darevskia derjugini	Lacertidae	Lacertinae	MH481130	MH481130	[117]
Darevskia mixta	Lacertidae	Lacertinae	MG704917	MG704917	[117]
Darevskia parvula	Lacertidae	Lacertinae	MG704918	MG704918	[117]
Darevskia portschinskii	Lacertidae	Lacertinae	MG704919	MG704919	[117]
Darevskia praticola	Lacertidae	Lacertinae	MH481132	MH481132	[117]
Darevskia raddei	Lacertidae	Lacertinae	MH481133	MH481133	[117]
Darevskia rudis	Lacertidae	Lacertinae	MG704920	MG704920	[117]
Darevskia saxicola	Lacertidae	Lacertinae	MG704921	MG704921	[117]
Darevskia unisexualis	Lacertidae	Lacertinae	KX644918	KX644918	[118]
Darevskia valentini	Lacertidae	Lacertinae	MG655240	MG655240	[117]
Dinarolacerta montenegrina	Lacertidae	Lacertinae	GQ142141	GQ142078	[49]
Dinarolacerta mosorensis	Lacertidae	Lacertinae	MN015187	KX080570	[113]; [116]
Eremias argus	Lacertidae	Eremiainae	JQ086345	JQ086345	Kim *et al.* (Unpubl. data)
Eremias arguta	Lacertidae	Eremiainae	KU605241	KU605241	Yu and Lin (Unpubl. data)
Eremias brenchleyi	Lacertidae	Eremiainae	EF490071	EF490071	[119]
Eremias dzungarica	Lacertidae	Eremiainae	MW250881	MW250881	[120]
Eremias grammica	Lacertidae	Eremiainae	KU585904	KU585904	Yu and Lin (Unpubl. data)
Eremias multiocellata	Lacertidae	Eremiainae	MK261077	MK261077	[121]

Eremias nikolskii	Lacertidae	Eremiainae	OK587334	OK587334	[122]
Eremias persica	Lacertidae	Eremiainae	MT554453	FJ445258	[123];[124]
Eremias przewalskii	Lacertidae	Eremiainae	KM507330	KM507330	[125]
Eremias strauchi strauchi	Lacertidae	Eremiainae	JQ690099	JQ690168	[126]
Eremias stummeri	Lacertidae	Eremiainae	KT372881	KT372881	[127]
Eremias szczerbaki	Lacertidae	Eremiainae	OL457296	OL457296	[128]
Eremias velox	Lacertidae	Eremiainae	KM359148	KM359148	[127]
Eremias vermiculata	Lacertidae	Eremiainae	KM104865	KM104865	[129]
Eremias yarkandensis	Lacertidae	Eremiainae	OK585048	OK585048	[130]
Gallotia atlantica	Lacertidae	Gallotiinae	MW496111	MW496111	[110]
Gallotia caesaris caesaris	Lacertidae	Gallotiinae	AF439948	AF439943	[131]
Gallotia caesaris gomerae	Lacertidae	Gallotiinae	AY151842	AY151921	[132]
Gallotia galloti eisentrauti	Lacertidae	Gallotiinae	AM489583	AF439942	Hernandez (Unpub. Data); [131]
Gallotia galloti galloti	Lacertidae	Gallotiinae	AM489592	AY151919	Hernandez (Unpub. Data); [132]
Gallotia galloti palmae	Lacertidae	Gallotiinae	AF439946	AF439941	[131]
Gallotia intermedia	Lacertidae	Gallotiinae	AY151844	AY151923	[132]
Gallotia simonyi machadoi	Lacertidae	Gallotiinae	AF101219	AY151924	[132,133]
Gallotia stehlini	Lacertidae	Gallotiinae	MN015165	AF439944	[113]; [131]
Gerrhosaurus nigrolineatus	Gerrhosauridae	Gallotiinae	EU116512	HQ167134	[134,135]
Heliobolus lugubris	Lacertidae	Eremiainae	JX962945	JX962890	[136]
Heliobolus spekii	Lacertidae	Eremiainae	AF206544	AF042550	[111]; [112]
Hellenolacerta graeca	Lacertidae	Lacertinae	MN015180	GQ142077	[113]; [49]
Iberolacerta aranica	Lacertidae	Lacertinae	MN015141	AF440596	[113]; [115]
Iberolacerta aurelioi	Lacertidae	Lacertinae	MN015164	AF440595	[113]; [115]
Iberolacerta bonnali	Lacertidae	Lacertinae	MN015147	AF440598	[113]; [115]
Iberolacerta cyreni	Lacertidae	Lacertinae	MN015184	AF440592	[113]; [115]
Iberolacerta galani	Lacertidae	Lacertinae	MN015193	DQ497130	[113]; [137]
Iberolacerta horvathi	Lacertidae	Lacertinae	MN015190	AY256653	[113]; [137]
Iberolacerta monticola	Lacertidae	Lacertinae	MN015138	AF440590	[113]; [115]
Ichnotropis capensis	Lacertidae	Eremiainae	MN015176	JX962877	[113]; [136]
Iranolacerta brandtii	Lacertidae	Lacertinae	MN015199	GQ142088	[113]; [49]
Lacerta agilis	Lacertidae	Lacertinae	KC990830	KC990830	[138]
Lacerta bilineata	Lacertidae	Lacertinae	KT722705	KT722705	[139]
Lacerta schreiberi	Lacertidae	Lacertinae	AF206591	MN015139	[111]; [113]
Lacerta strigata	Lacertidae	Lacertinae	MW592672	DQ097094	[140]; [141]
Lacerta trilineata	Lacertidae	Lacertinae	MN015167	AJ238177	[113]; [142]
Lacerta viridis viridis	Lacertidae	Lacertinae	AM176577	AM176577	[143]
Latastia longicaudata	Lacertidae	Eremiainae	JX962946	JX962891	[136]
Meroles cuneirostris	Lacertidae	Eremiainae	JX962929	JX962874	[136]
Meroles knoxii	Lacertidae	Eremiainae	JX962928	JX962873	[136]
Meroles squamulosus	Lacertidae	Eremiainae	MW496120	MW496120	[110]
Meroles suborbitalis	Lacertidae	Eremiainae	JX962927	JX962871	[136]
Mesalina brevirostris	Lacertidae	Eremiainae	AF206606	FJ416173	[111]; [124]
Mesalina olivieri	Lacertidae	Eremiainae	MW496114	MW496114	[110]
Nucras lalandii	Lacertidae	Eremiainae	JX962944	JX962889	[136]
Nucras tessellata	Lacertidae	Eremiainae	AF206612	MW823898	[111]; [144]

Ophisops elegans	Lacertidae	Eremiainae	GQ142116	GQ142069	[49]
Parvilacerta parva	Lacertidae	Lacertinae	GQ142135	GQ142082	[49]
Pedioplanis gaerdesi	Lacertidae	Eremiainae	MW823691	AY192434	[144]; [145]
Pedioplanis inornata	Lacertidae	Eremiainae	MW823830	AY192435	[144]; [145]
Pedioplanis laticeps	Lacertidae	Eremiainae	MW496119	MW496119	[110]
Pedioplanis lineoocellata	Lacertidae	Eremiainae	JX962943	JX962888	[136]
Pedioplanis undata	Lacertidae	Eremiainae	JX962942	AF042549	[136]; [112]
Phoenicolacerta kulzeri	Lacertidae	Lacertinae	FJ460596	FJ460596	[146]
Phoenicolacerta laevis	Lacertidae	Lacertinae	MN015197	KR082613	[113]; [147]
Podarcis bocagei	Lacertidae	Lacertinae	MN015135	AJ250168	[113]; [148]
Podarcis carbonelli	Lacertidae	Lacertinae	MN015143	AF469418	[113]; [149]
Podarcis erhardii	Lacertidae	Lacertinae	AF486218	MW619230	[150]; [151]
Podarcis filfolensis	Lacertidae	Lacertinae	MN015146	AJ001464	[113]; [151]
Podarcis lilfordi	Lacertidae	Lacertinae	AM747719	AJ250159	[153]; [148]
Podarcis liolepis	Lacertidae	Lacertinae	MN015188	KT030702	[113]; [154]
Podarcis melisellensis	Lacertidae	Lacertinae	MT010548	AY185002	[155]; [156]
Podarcis milensis	Lacertidae	Lacertinae	MN015166	MW619252	[113]; [151]
Podarcis muralis	Lacertidae	Lacertinae	FJ460597	FJ460597	[146]
Podarcis pityusensis	Lacertidae	Lacertinae	JX852048	AJ250158	[157]; [148]
Podarcis siculus	Lacertidae	Lacertinae	FJ460598	FJ460598	[146]
Podarcis tauricus	Lacertidae	Lacertinae	ON155608	MW619273	[158];[151]
Podarcis tiliguerta	Lacertidae	Lacertinae	MN015144	AJ001479	[113]; [148]
Podarcis waglerianus	Lacertidae	Lacertinae	MN015189	AJ001466	[113]; [152]
Psammodromus algirus	Lacertidae	Gallotiinae	MW496117	MW496117	[110]
Scelarcis perspicillata	Lacertidae	Lacertinae	MN015156	GQ142074	[113]; [49]
Takydromus amurensis	Lacertidae	Lacertinae	KU641018	KU641018	[159]
Takydromus formosanus	Lacertidae	Lacertinae	KM487175	AY248532	[50]; [160]
Takydromus hsuehshanensis	Lacertidae	Lacertinae	AF217051	AY248483	[161]; [160]
Takydromus intermedius	Lacertidae	Lacertinae	MN239958	AY032597	[162]; [161]
Takydromus kuehnei	Lacertidae	Lacertinae	MZ435950	MZ435950	[163]
Takydromus septentrionalis	Lacertidae	Lacertinae	MK630237	MK630237	[164]
Takydromus sexlineatus	Lacertidae	Lacertinae	KF425529	KF425529	[165]
Takydromus stejnegeri	Lacertidae	Lacertinae	AF217072	AY248477	[161]; [160]
Takydromus sylvaticus	Lacertidae	Lacertinae	JX290083	JX290083	[166]
Takydromus tachydromoides	Lacertidae	Lacertinae	AB080237	AB080237	[166]
Takydromus wolteri	Lacertidae	Lacertinae	JX181764	JX181764	[167]
Teira dugesii	Lacertidae	Lacertinae	MN015169	KP668869	[113]; [168]
Timon lepidus	Lacertidae	Lacertinae	MN015137	GQ142071	[113]; [49]
Timon princeps	Lacertidae	Lacertinae	MN015170	JQ425790	[113]; [169]
Tropidosaura cottrelli	Lacertidae	Eremiainae	JX962939	JX962884	[136]
Tropidosaura essexi	Lacertidae	Eremiainae	JX962938	JX962883	[136]
Tropidosaura gularis	Lacertidae	Eremiainae	JX962933	AF080371	[136];
Tropidosaura montana	Lacertidae	Eremiainae	JX962935	JX962881	[136]
Zootoca vivipara	Lacertidae	Lacertinae	KM401599	KM401599	[170]

References

49. Pavlicev, M.; Mayer, W. Fast radiation of the subfamily Lacertinae (Reptilia: Lacertidae): History or methodical artefact? *Mol. Phylogenet. Evol.* **2009**, *52*, 727–734. https://doi.org/10.1016/j.ympev.2009.04.020.
50. Tseng, S.P.; Li, S.H.; Hsieh, C.H.; Wang, H.Y.; Lin, S.M. Influence of gene flow on divergence dating-implications for the speciation history of *Takydromus* grass lizards. *Mol. Ecol.* **2014**, *23*, 4770–4784. https://doi.org/10.1111/mec.12889.
110. Kirchhof, S.; Lyra, M.L.; Rodriguez, A.; Ineich, I.; Müller, J.; Rödel, M.O., Trape, J.F.; Vences, M.; Boissinot, S. Mitogenome analyses elucidate the evolutionary relationships of a probable Eocene wet tropics relic in the xerophile lizard genus *Acanthodactylus*. *Sci. Rep.* **2021**, *11*, 4858. https://doi.org/10.1038/s41598-021-83422-7.
111. Fu, J. Toward the phylogeny of the family Lacertidae–why 4708 base pairs of mtDNA sequences cannot draw the picture. *Biol. J. Linn. Soc.* **2000**, *71*, 203–217. https://doi.org/10.1111/j.1095-8312.2000.tb01254.x.
112. Harris, D.J.; Arnold, E.N.; Thomas, R.H. Relationships of lacertid lizards (Reptilia: Lacertidae) estimated from mitochondrial DNA sequences and morphology. *Proc. Biol. Sci.* **1998**, *265*, 1939–1948. https://doi.org/10.1098/rspb.1998.0524.
113. Garcia-Porta, J.; Irisarri, I.; Kirchner, M.; Rodríguez, A.; Kirchhof, S.; Brown, J.L.; MacLeod, A.; Turner, A.P.; Ahmadzadeh, F.; Albaladejo, G.; et al. Environmental temperatures shape thermal physiology as well as diversification and genome-wide substitution rates in lizards. *Nat. Commun.* **2019**, *10*, 4077. https://doi.org/10.1038/s41467-019-11943-x.
114. Macey, J.R.; Papenfuss, T.J.; Kuehl, J.V.; Fourcade, H.M.; Boore, J.L. Phylogenetic relationships among amphisbaenian reptiles based on complete mitochondrial genomic sequences. *Mol. Phylogenet. Evol.* **2004**, *33*, 22–31. https://doi.org/10.1016/j.ympev.2004.05.003.
115. Mayer, W., Arribas, O. Phylogenetic relationships of the European lacertid genera *Archaeolacerta* and *Iberolacerta* and their relationships to some other 'Archaeolacertae' (*sensu lato*) from Near East, derived from mitochondrial DNA sequences. *J. Zool. Syst. Evol. Res.* **2003**, *41*, 157–161. https://doi.org/10.1046/j.1439-0469.2003.00223.x.
116. Mendes, J.; Harris, D.J.; Carranza, S.; Salvi, D. Evaluating the phylogenetic signal limit from mitogenomes, slow evolving nuclear genes, and the concatenation approach. New insights into the Lacertini radiation using fast evolving nuclear genes and species trees. *Mol. Phylogenet. Evol.* **2016**, *100*, 254–267. https://doi.org/10.1016/j.ympev.2016.04.016.
117. Murtskhvaladze, M.; Tarkhnishvili, D.; Anderson, C.L.; Kotorashvili, A. Phylogeny of caucasian rock lizards (*Darevskia*) and other true lizards based on mitogenome analysis: Optimisation of the algorithms and gene selection. *PLoS One* **2020**, 15, e0233680. https://doi.org/10.1371/journal.pone.0233680.
118. Komissarov, A.; Korchagin, V.; Kliver, S.; Dobrynin, P.; Semyenova, S.; Vergun, A.; O'Brien, S.; Ryskov, A. The complete mitochondrial genome of the parthenogenetic Caucasian rock lizard *Darevskia unisexualis* (Squamata: Lacertidae) contains long tandem repeat formed by 59 bp monomer. *Mitochondrial DNA B* **2016**, *1*, 875–877. https://doi.org/10.1080/23802359.2016.1253040.
119. Rui, J.L.; Wang, Y.T.; Nie, L.W. The complete mitochondrial DNA genome of *Eremias brenchleyi* (Reptilia: Lacertidae) and its phylogeny position within Squamata reptiles. *Amphib-Reptil.* **2009**, *30*, 25–35. https://doi.org/10.1163/156853809787392793.
120. Wang, S.; Liu, J.; Zhang, B.; Guo, X. The complete mitochondrial genome of *Eremias dzungarica* (Reptilia, Squamata, Lacertidae) from the Junggar Basin in Northwest China. *Mitochondrial DNA B* **2021**, *6*, 2012–2014. https://doi.org/10.1080/23802359.2021.1923417.
121. Su, X.; Liu, J.; Chen, D.; Guo X. Next-generation sequencing yields a nearly complete mitochondrial genome of the multiocellated racerunner (*Eremias multiocellata*) in Northwest China. *Mitochondrial DNA B* **2019**, *4*, 1430–1431. https://doi.org/10.1080/23802359.2019.1598810.
122. Guo, X.; Huo, X.; Liu, J.; Chirikova, M.A. Complete mitochondrial genome of the Kyrghyz racerunner (*Eremias nikolskii* Bedriaga, 1905) from Kyrgyzstan. *Mitochondrial DNA B* **2022**, *7*, 983–985. https://doi.org/10.1080/23802359.2022.2080599.
123. Khan, M.A.; Jablonski, D.; Nadeem, M.S.; Masroor, R.; Kehlmaier, C.; Spitzweg, C.; Fritz, U. Molecular phylogeny of *Eremias* spp. from Pakistan contributes to a better understanding of the diversity of racerunners. *J. Zool. Syst. Evol. Res.* **2021**, *59*, 466–483. https://doi.org/10.1111/jzs.12426.
124. Pouyani, E.R.; Pouyani, N.R.; Noureini, S.K.; Joger, U.; Wink, M. Molecular phylogeny of the *Eremias persica* complex of the Iranian plateau (Reptilia: Lacertidae), based on mtDNA sequences. *Zool. J. Linn. Soc.* **2010**, *158*, 641–660. https://doi.org/10.1111/j.1096-3642.2009.00553.x.
125. Du, Y.; Qiu, Q.B.; Tong, Q.L.; Lin, L.H. The complete mitochondrial genome of *Eremias przewalskii* (Squamata: Lacertidae). *Mitochondrial DNA A* **2016**, *27*, 1918–1919. https://doi.org/10.3109/19401736.2014.971286.
126. Pouyani, E.R.; Noureini, S.K.; Pouyani, N.R.; Joger, U.; Wink, M. Molecular phylogeny and intraspecific differentiation of the *Eremias velox* complex of the Iranian plateau and Central Asia (Sauria, Lacertidae). *J. Zool. Syst. Evol. Res.* **2012**, *50*, 220–229. https://doi.org/10.1111/j.1439-0469.2012.00662.x.
127. Zhou, T.; Li, D.; Dujsebayeva, T.N.; Liu, J.; Guo, X. Complete mitochondrial genome of Stummer's racerunner (*Eremias stummeri*) from Kazakhstan. *Mitochondrial DNA A* **2016**, *27*, 4340–4341. https://doi.org/10.3109/19401736.2015.1089491.

128. Tian, L.L.; Guo, X.G. Complete mitochondrial genomes of five racerunners (Lacertidae: *Eremias*) and comparison with other lacertids: Insights into the structure and evolution of the control region. *Genes* **2022**, *13*, 726. https://doi.org/10.3390/genes13050726.
129. Tong, Q.L.; Yao, Y.T.; Lin, L.H.; Ji, X. The complete mitochondrial genome of *Eremias vermiculata* (Squamata: Lacertidae). *Mitochondrial DNA A* **2016**, *27*, 1447–1448. https://doi.org/10.3109/19401736.2014.953086.
130. Wang, S.; Liu, J.; Chirikova, M.A.; Zhang, B.; Guo, X. The complete mitochondrial genome of *Eremias yarkandensis* (Reptilia, Squamata, Lacertidae) from Kyrgyzstan. *Mitochondrial DNA B* **2022**, *7*, 443–445. https://doi.org/10.1080/23802359.2022.2047119.
131. Maca-Meyer, N.; Carranza, S.; Rando, J.C.; Arnold, E.N.; Cabrera, V.M. Status and relationships of the extinct giant Canary Island lizard *Gallotia goliath* (Reptilia: Lacertidae), assessed using ancient mtDNA from its mummified remains. *Biol. J. Linn. Soc.* **2003**, *80*, 659–670. https://doi.org/10.1111/j.1095-8312.2003.00265.x.
132. Carranza, S.; Arnold, E.N.; Amat, F. DNA phylogeny of Lacerta (*Iberolacerta*) and other lacertine lizards (Reptilia: Lacertidae): did competition cause long-term mountain restriction? *Syst. Biodivers.* **2004**, *2*, 57–77. https://doi.org/10.1017/S1477200004001355.
133. Carranza, S.; Arnold, E.N.; Thomas, R.H.; López-Jurado, L.F. Status of the extinct giant lacertid lizard *Gallotia simonyi simonyi* (Reptilia: Lacertidae) assessed using mtDNA sequences from museum specimens. *Herpetol. J.* **1999**, *9*, 83–86.
134. Noonan, B.P.; Pramuk, J.B.; Bezy, R.L.; Sinclair, E.A.; de Queiroz, K.; Sites Jr, J.W. Phylogenetic relationships within the lizard clade Xantusiidae: Using trees and divergence times to address evolutionary questions at multiple levels. *Mol. Phylogenet. Evol.* **2013**, *69*, 109–122. https://doi.org/10.1016/j.ympev.2013.05.017.
135. Stanley, E.L.; Bauer, A.M.; Jackman, T.R.; Branch, W.R.; Mouton, P.L.F.N. Between a rock and a hard polytomy: rapid radiation in the rupicolous girdled lizards (Squamata: Cordylidae). *Mol. Phylogenet. Evol.* **2011**, *58*, 53–70. https://doi.org/10.1016/j.ympev.2010.08.024.
136. Engleder, A.; Haring, E.; Kirchhof, S.; Mayer, W. Multiple nuclear and mitochondrial DNA sequences provide new insights into the phylogeny of South African Lacertids (Lacertidae, Eremiadinae). *J. Zool. Syst. Evol. Res.* **2013**, *51*, 132–143. https://doi.org/doi.org/10.1111/jzs.12012.
137. Arribas, O.; Carranza, S.; Odierna, G. Description of a new endemic species of mountain lizard from Northwestern Spain: *Iberolacerta galani* sp. nov. (Squamata: Lacertidae). *Zootaxa* **2006**. *1240*, 1–55. https://doi.org/10.5281/zenodo.172862.
138. Qin, P.S.; Tao, C.R.; Yin, S.; Li, H.M.; Zeng, D.L.; Qin, X.M. Complete mitochondrial genome of *Lacerta agilis* (Squamata, Lacertidae). *Mitochondrial DNA* **2014**, *25*, 416–417. https://doi.org/10.3109/19401736.2013.809436.
139. Kolora, S.R.; Faria, R.; Weigert, A.; Schaffer, S.; Grimm, A.; Henle, K.; Sahyoun, A.H.; Stadler, P.F.; Nowick, K.; Bleidorn, C.; et al. The complete mitochondrial genome of *Lacerta bilineata* and comparison with its closely related congener *L. viridis*. *Mitochondrial DNA A* **2015**, *28*, 116–118. https://doi.org/10.3109/19401736.2015.1111349.
140. Saberi-Pirooz, R.; Rajabi-Maham, H.; Ahmadzadeh, F.; Kiabi, B.H.; Javidkar, M.; Carretero, M.A. Pleistocene climate fluctuations as the major driver of genetic diversity and distribution patterns of the Caspian green lizard, *Lacerta strigata* Eichwald, 1831. *Ecol. Evol.* **2021**, *11*, 6927–6940. https://doi.org/10.1002/ece3.7543.
141. Godinho, R.; Crespo, E.; Ferrand, N.; Harris, D.J. Phylogeny and evolution of the green lizards, *Lacerta* spp. (Squamata: Lacertidae) based on mitochondrial and nuclear DNA sequences. *Amphib-Reptil* **2005**, *26*, 271–285. DOI: 10.1163/156853805774408667.
142. Beyerlein, P.; Mayer, W. *Lacerta kulzeri*-Its phylogenetic relationships as indicated by DNA sequences. *Natura Croatica* **1999**, *8*, 181.
143. Böhme, M.U.; Fritzsch, G.; Tippmann, A.; Schlegel, M.; Berendonk, T.U. The complete mitochondrial genome of the green lizard *Lacerta viridis viridis* (Reptilia: Lacertidae) and its phylogenetic position within squamate reptiles. *Gene* **2007**, *394*, 69–77. https://doi.org/10.1016/j.gene.2007.02.006.
144. Childers, J.L.; Kirchhof, S.; Bauer, A.M. Lizards of a different stripe: phylogenetics of the *Pedioplanis undata* species complex (Squamata, Lacertidae), with the description of two new species. *Zoosyst. Evol.* **2021**, *97*, 249–272. https://doi.org/10.3897/zse.97.61351.
145. Lamb, T.; Bauer, A.M. *Meroles* revisited: complementary systematic inference from additional mitochondrial genes and complete taxon sampling of southern Africa's desert lizards. *Mol. Phylogenet. Evol.* **2003**, *29*, 360–364. https://doi.org/10.1016/S1055-7903(03)00137-4.
146. Podnar, M.; Pinsker, W.; Mayer, W. Complete mitochondrial genomes of three lizard species and the systematic position of the Lacertidae (Squamata).*J. Zool. Syst. Evol. Res.* **2009**, *47*, 35–41. https://doi.org/10.1111/j.1439-0469.2008.00515.x.
147. Tamar, K.; Carranza, S.; In den Bosch, H.; Sindaco, R.; Moravec, J.; Meiri, S. Hidden relationships and genetic diversity: Molecular phylogeny and phylogeography of the Levantine lizards of the genus *Phoenicolacerta* (Squamata: Lacertidae). *Mol. Phylogenet. Evol.* **2015**, *91*, 86–97. https://doi.org/10.1016/j.ympev.2015.05.002.
148. Oliverio, M.; Bologna, M.A.; Mariottini, P. Molecular biogeography of the Mediterranean lizards *Podarcis* Wagler, 1830 and *Teira* Gray, 1838 (Reptilia, Lacertidae). *J. Biogeogr.* **2000**, *27*, 1403–1420. https://doi.org/10.1046/j.1365-2699.2000.00517.x.

149. Harris, D.J.; Sa-Sousa, P. Molecular phylogenetics of Iberian wall lizards (*Podarcis*): is *Podarcis hispanica* a species complex? *Mol. Phylogenet. Evol.* **2002**, *23*, 75–81. https://doi.org/10.1163/156853805774408667
150. Poulakakis, N.; Lymberakis, P.; Antoniou, A.; Chalkia, D.; Zouros, E.; Mylonas, M.; Valakos, E. Molecular phylogeny and biogeography of the wall-lizard *Podarcis erhardii* (Squamata: Lacertidae). *Mol. Phylogenet. Evol.* **2003**, *28*, 38–46. https://doi.org/10.1016/s1055-7903(03)00037-x.
151. Salvi, D.; Pinho, C.; Mendes, J.; Harris, D.J. Fossil-calibrated time tree of *Podarcis* wall lizards provides limited support for biogeographic calibration models.*Mol. Phylogenet. Evol.* **2021**, *161*, 107–169. https://doi.org/10.1016/j.ympev.2021.107169.
152. Oliverio, M.; Bologna, M.A.; Monciotti, A.; Annesi, F.; Mariottini, P. Molecular phylogenetics of the Italian *Podarcis* lizards (Reptilia, Lacertidae). *Ital. J. Zool.* **1998**, *65*, 315–324. https://doi.org/10.1046/j.1365-2699.2000.00517.x.
153. Brown, R.P.; Terrasa, B.; Pérez-Mellado, V.; Castro, J.A.; Hoskisson, P.A.; Picornell, A.; Ramon, M.M. Bayesian estimation of post-Messinian divergence times in Balearic Island lizards. *Mol. Phylogenet. Evol.* **2008**, *48*, 350–358. https://doi.org/10.1016/j.ympev.2008.04.013.
154. Rodríguez, V.; Buades, J.M.; Brown, R.P. Terrasa, B.; Pérez-Mellado, V.; Corti, C.; Delaugerre, M.; Castro, J.A; Picornell, A.; Ramon, M.M. Evolutionary history of *Podarcis tiliguerta* on Corsica and Sardinia. *BMC Evol. Biol.* **2017**, *17*, 1–12. https://doi.org/10.1186/s12862-016-0860-4.
155. Taverne, M.; Gillies, N.K.; Krajnović, M.; Lisičić, D.; Mira, Ó.; Petricioli, D.; Sabolić, I.; Štambuk, A.; Tadić, Z.; Vigliotti, C.; et al. Proximate and ultimate drivers of variation in bite force in the insular lizards *Podarcis melisellensis* and *Podarcis sicula*. *Biol. J. Linn. Soc.* **2020**, *131*, 88–108. https://doi.org/10.1093/biolinnean/blaa091.
156. Podnar, M.; Mayer, W.; Tvrtković, N. Mitochondrial phylogeography of the Dalmatian wall lizard, *Podarcis melisellensis* (Lacertidae). *Org. Divers. Evol.* **2004**, *4*, 307–317. https://doi.org/10.1111/j.1439-0469.2008.00515.x.
157. Buades, J.M.; Rodríguez, V.; Terrasa, B.; Pérez-Mellado, V.; Brown, R.P.; Castro, J.A.; Picornell, A.; Ramon, M.M. Variability of the mc1r Gene in Melanic and Non-Melanic *Podarcis lilfordi* and *Podarcis pityusensis* from the Balearic Archipelago. *PLoS One* **2013**, *8*, e53088. https://doi.org/10.1371/journal.pone.0053088.
158. Rehák, I.; Fischer, D.; Kratochvíl, L.; Rovatsos, M. Origin and haplotype diversity of the northernmost population of *Podarcistauricus* (Squamata, Lacertidae): Do lizards respond to climate change and go north? *Biodiversity Data Journal* **2022**, *10*, e82156. https://doi.org/10.3897/BDJ.10.e82156.
159. Ma, W.W.; Liu, H.; Zhao, W.G.; Liu, P. The complete mitochondrial genome of *Takydromus amurensis* (Squamata: Lacertidae). *Mitochondrial DNA B* **2016**, *1*, 214–215. https://doi.org/10.1080/23802359.2016.1155091.
160. Tseng, S.P.; Wang, C.J.; Li, S.H.; Lin, S.M. Within-island speciation with an exceptional case of distinct separation between two sibling lizard species divided by a narrow stream. *Mol. Phylogenet. Evol.* **2015**, *90*, 164–75. https://doi.org/10.1016/j.ympev.2015.04.022.
161. Lin, S.M.; Chen, C.A.; Lue, K.Y. Molecular phylogeny and biogeography of the grass lizards genus *Takydromus* (Reptilia: Lacertidae) of East Asia. *Mol. Phylogenet. Evol.* **2002**, *22*, 276–288. https://doi.org/10.1006/mpev.2001.1059.
162. Wang, J.; Lyu, Z.T.; Yang, C.Y.; Li, Y.L.; Wang, Y.Y. A new species of the genus *Takydromus* (Squamata, Lacertidae) from southwestern Guangdong, China. *ZooKeys* **2019**, *871*, 119. https://doi.org/10.3897/zookeys.871.35947.
163. Wu, L.X.; Luo, K.N.; Ding, G.H. Complete mitochondrial genome of *Takydromus kuehnei* (Squamata: *Takydromus*) and its phylogenetic analysis. *Mitochondrial DNA B* **2022**, *7*, 764–765. https://doi.org/10.1080/23802359.2022.2070440.
164. Hu, J.G.; Peng, L.F.; Tang, X.S.; Huang, S. The complete mitochondrial genome of *Takydromus septentrionalis* (Reptilia: Lacertidae). *Mitochondrial DNA B* **2019**, *4*, 2193–2194. https://doi.org/10.1080/23802359.2019.1623123.
165. Qin, P.S.; Zeng, D.L.; Hou, L.X.; Yang, X.W.; Qin, X.M. Complete mitochondrial genome of *Takydromus sexlineatus* (Squamata, Lacertidae). *Mitochondrial DNA* **2015**, *26*, 465–466. https://doi.org/10.3109/19401736.2013.830299.
166. Tang, X.S.; Chen, J.M.; Huang, S. Mitochondrial genome of the Chung-an ground lizard *Takydromus sylvaticus* (Reptilia: Lacertidae). *Mitochondrial DNA* **2014**, *25*, 319–320. https://doi.org/10.3109/19401736.2013.800488.
167. Yu, D.N.; Ji, X. The complete mitochondrial genome of *Takydromus wolteri* (Squamata: Lacertidae). *Mitochondrial DNA* **2013**, *24*, 3–5. https://doi.org/10.3109/19401736.2012.710223.
168. Silva-Rocha, I.; Sá-Sousa, P.; Fariña, B.; Carretero, M.A. Molecular analysis confirms Madeira as source for insular and continental introduced populations of *Teira dugesii* (Sauria: Lacertidae). *Salamandra* **2016**, *523*, 269–272.
169. Ahmadzadeh, F.; Carretero, M.A.; Harris, D.J.; Perera, A.; Böhme, W. A molecular phylogeny of the eastern group of ocellated lizard genus *Timon* (Sauria: Lacertidae) based on mitochondrial and nuclear DNA sequences. *Amphib-Reptil* **2012**, *33*, 1–10. https://doi.org/10.1163/156853811x619718.
170. Liu, P.; Zhu, D.; Zhao, W.G.; Ji, X. The complete mitochondrial genome of the common lizard *Zootoca vivipara* (Squamata: Lacertidae). *Mitochondrial DNA A* **2016**, *27*, 1944–1945. https://doi.org/10.3109/19401736.2014.971299.

Table S4 Climatic factors from WorldClim used in ENM and their contribution rates.

ID	Environment Variable	Contribution Rates with SD	Permutation importance
Bio1	Annul mean temperature	38.28±6.20	31.14
Bio2	Mean diurnal range	1.21±0.80	2.28
Bio3	Isothermality	0.34±0.40	0.17
Bio4	Temperature seasonality	9.34±3.08	1.00
Bio5	Max Temperature of Warmest Month	2.53±2.07	9.28
Bio12	Annual precipitation	21.50±4.23	50.25
Bio14	Precipitation of driest month	15.30±8.00	0.94
Bio15	Precipitation seasonality (coefficient of variation)	11.56±6.68	4.95

Table S5 Climatic factors from PaleoClim used in ENM and their contribution rates.

ID	Environment Variable	Contribution Rates with SD	Permutation importance
Bio1	Annul mean temperature	41.61±4.86	51.93
Bio4	Temperature seasonality	12.44±3.07	3.474
Bio12	Annual precipitation	20.13±2.07	42.54
Bio14	Precipitation of driest month	21.61±8.49	0.71
Bio15	Precipitation seasonality (coefficient of variation)	4.21±4.08	1.36

Table S6 Occurrence records with coordinates for *Eremias argus* retrieved from literature and GBIF.

Site number	Longitude (E)	Latitude (N)	Reference
1	126.89	38.33	[23]
2	125.83	39.07	[23]
3	128.12	38.65	[23]
4	126.35	40.42	[23]
5	117.13	42.2	[25]
6	118.3	32.3	[32]
7	108.9	34	[32]
8	112.6	35.1	[32]
9	100.5	36.4	[32]
10	114.5	36.6	[32]
11	113.6	37.9	[32]
12	107.5	39.5	[32]
13	126.6	45.7	[32]
14	101.71	43.1	[77]
15	100.43	43.66	[77]
16	103.72	44.13	[77]
17	110.13	44.69	[77]
18	113.13	45.3	[77]
19	105.42	45.48	[77]
20	112.68	45.67	[77]
21	97.2	45.75	[77]
22	108.64	46.37	[77]
23	91.54	46.5	[77]
24	113.79	46.94	[77]
25	114.18	47.29	[77]
26	92.18	47.48	[77]
27	104.17	47.83	[77]
28	104.31	47.84	[77]
29	114.46	48.06	[77]
30	101.92	43.22	[78]
31	104.42	43.58	[78]
32	112.75	43.73	[78]
33	98.87	45.45	[78]
34	112.77	45.7	[78]
35	117.67	46.83	[78]
36	102.83	49.42	[78]
37	106.25	42.46	[79]
38	105.25	42.48	[79]
39	106.79	42.53	[79]
40	105.34	42.65	[79]
41	109.9	42.73	[79]
42	109.71	42.8	[79]
43	109.64	42.86	[79]
44	98.66	42.87	[79]

45	98.82	42.88	[79]
46	98.64	42.93	[79]
47	98.09	42.95	[79]
48	98.66	42.96	[79]
49	98.69	42.98	[79]
50	108.91	43	[79]
51	98.71	43.01	[79]
52	109.38	43.04	[79]
53	101.16	43.07	[79]
54	109.15	43.08	[79]
55	101.56	43.09	[79]
56	97.98	43.11	[79]
57	107.48	43.11	[79]
58	102.02	43.16	[79]
59	102	43.18	[79]
60	107.2	43.19	[79]
61	97.87	43.21	[79]
62	105.8	43.22	[79]
63	101.05	43.23	[79]
64	98.99	43.25	[79]
65	99.01	43.25	[79]
66	100.98	43.26	[79]
67	102.16	43.28	[79]
68	106.09	43.3	[79]
69	97.79	43.31	[79]
70	109.15	43.33	[79]
71	105.01	43.35	[79]
72	102.51	43.38	[79]
73	102.43	43.39	[79]
74	103.92	43.4	[79]
75	99.11	43.41	[79]
76	100.57	43.44	[79]
77	102.84	43.44	[79]
78	103.41	43.44	[79]
79	101.24	43.48	[79]
80	102.91	43.48	[79]
81	102.94	43.49	[79]
82	97.91	43.52	[79]
83	100.03	43.54	[79]
84	103.02	43.55	[79]
85	104.04	43.55	[79]
86	100.07	43.58	[79]
87	99.14	43.61	[79]
88	110.58	43.62	[79]
89	97.96	43.63	[79]
90	101.18	43.63	[79]

91	99.16	43.65	[79]
92	101.23	43.65	[79]
93	97.95	43.68	[79]
94	100.95	43.72	[79]
95	100.94	43.75	[79]
96	97.98	43.98	[79]
97	99.73	43.98	[79]
98	101.39	43.99	[79]
99	97.98	44.13	[79]
100	99.46	44.13	[79]
101	99.26	44.17	[79]
102	110.23	44.18	[79]
103	98.13	44.39	[79]
104	98.17	44.56	[79]
105	99.3	44.63	[79]
106	94.92	44.66	[79]
107	97.56	44.73	[79]
108	110.14	44.79	[79]
109	97.32	44.81	[79]
110	94.96	44.84	[79]
111	110.16	44.87	[79]
112	94.98	44.89	[79]
113	96.25	44.93	[79]
114	96.78	44.93	[79]
115	96.26	44.94	[79]
116	96.82	45.06	[79]
117	95.13	45.12	[79]
118	92.16	45.13	[79]
119	95.44	45.14	[79]
120	95.45	45.14	[79]
121	95.49	45.14	[79]
122	109.98	45.15	[79]
123	91.41	45.17	[79]
124	95.94	45.21	[79]
125	91.08	45.24	[79]
126	90.94	45.26	[79]
127	93.64	45.26	[79]
128	99.51	45.3	[79]
129	93.2	45.36	[79]
130	93.61	45.38	[79]
131	92.4	45.43	[79]
132	92.5	45.48	[79]
133	93.59	45.51	[79]
134	92.15	45.53	[79]
135	92.86	45.54	[79]
136	93.07	45.54	[79]

137	92.34	45.56	[79]
138	90.97	45.59	[79]
139	93.31	45.59	[79]
140	91.11	45.71	[79]
141	93.23	45.73	[79]
142	92.5	45.75	[79]
143	91.18	45.76	[79]
144	96.9	45.82	[79]
145	99.26	45.82	[79]
146	96.35	45.91	[79]
147	93.13	45.99	[79]
148	91.26	46.03	[79]
149	91.11	46.1	[79]
150	108.71	46.11	[79]
151	94.55	46.13	[79]
152	91.07	46.14	[79]
153	95.51	46.14	[79]
154	94.91	46.15	[79]
155	91.58	46.17	[79]
156	95.11	46.2	[79]
157	95.26	46.26	[79]
158	95.39	46.29	[79]
159	108.86	46.34	[79]
160	95.84	46.36	[79]
161	95.69	46.37	[79]
162	95.81	46.37	[79]
163	91.4	46.68	[79]
164	96.76	46.68	[79]
165	103.64	47.4	[79]
166	103.7	47.41	[79]
167	103.76	47.48	[79]
168	92.42	47.74	[79]
169	126.38	36.41	[88]
170	126.38	36.41	[171]
171	126.28	36.65	[172]
172	126.36	36.4	[173]
173	114.47	36.6	[174]
174	105.94	38.57	[175]
175	109.31	42.13	[176]
176	126.20	36.80	[177]
177	127.70	37.80	[177]
178	127.10	37.60	[177]
179	126.60	36.00	[177]
180	127.20	37.20	[177]
181	127.20	36.90	[177]
182	126.80	37.80	[177]

183	127.10	36.50	[177]
184	117.10	36.20	[177]
185	105.00	48.00	[177]
186	113.50	46.30	[177]
187	117.80	47.50	[177]
188	102.50	36.20	[177]
189	109.00	33.90	[177]
190	115.90	40.30	[177]
191	121.40	37.50	[177]
192	119.40	39.80	[177]
193	114.30	30.60	[177]
194	106.00	38.60	[177]
195	121.70	37.40	[177]
196	116.40	40.30	[177]
197	114.30	38.10	[177]
198	118.90	39.20	[177]
199	103.44	47.34	[177]
200	103.80	36.12	[177]
201	104.25	49.19	[177]
202	105.30	47.88	[177]
203	107.54	51.76	[177]
204	109.50	41.42	[177]
205	109.65	38.67	[177]
206	110.29	39.95	[177]
207	110.50	42.07	[177]
208	110.51	41.62	[177]
209	112.16	42.09	[177]
210	112.73	45.85	[177]
211	113.90	41.10	[177]
212	115.72	43.34	[177]
213	115.75	43.36	[177]
214	115.82	40.38	[177]
215	116.14	43.83	[177]
216	116.20	40.66	[177]
217	117.27	48.59	[177]
218	122.38	42.82	[177]
219	122.47	42.77	[177]
220	123.17	42.83	[177]

References

23. Szyndlar, Z. Distributional records for turtles and lizards from North Korea. *Herpetol. Rev.* **1991**, *22*, 27.

25. Zeng, Z.G.; Bi, J.H.; Li, S.R.; Chen, S.Y.; Du, W.G. Habitat alteration influences a desert steppe lizard community: implications of species specific preferences and performance. *Herpetol. Monogr.* **2016**, *30*, 34–48. https://doi.org/10.1655/HERPMONOGRAPHS-D-14-00008.1.

32. Zhao, Q.; Liu, H.X.; Luo, L.G.; Ji, X. Comparative population genetics and phylogeography of two lacertid lizards (*Eremias argus* and *E. brenchleyi*) from China. *Mol. Phylogenet. Evol.* **2011**, *58*, 478–491. https://doi.org/. https://doi.org/10.1016/j.ympev.2010.12.017.

77. Alberto, S.V.; Marta, C.; Mario, G.P.; Judit, V.; José, G.A. Amphibians and reptiles from Zoltan Kaszab's expeditions to Mongolia held at the Hungarian Natural History Museum. *Acta Zool. Acad. Sci. H.* **2019**, *65*, 143–166. https://doi.org/10.17109/AZH.65.2.143.2019.
78. Ananjeva, N.B.; Munkhbayar, K.; Orlov, N.L.; Orlova, V.F.; Semenov, D.V.; Terbish, K. *Amphibians and reptiles of Mongolia. Reptiles of Mongolia*; KMK Press: Moscow, Russia, 1997. (in Russian with English summary)
79. Buehler, M.D.; Zoljargal, P.; Purvee, E. Batsaikhan, N.; Ananjeva, N.B.; Orlov, N.L.; Panpenfuss, T.J.; Roldán-Piña, D. The results of four recent joint expedition to the Gobi Desert: lacertids and agamids. *Russ. J. Herpetol.* **2021**, *28*, 15–32. https://doi.org/10.30906/1026-2296-2021-28-1-15-32.
88. Chang, M.H.; Song, J.Y.; Koo, K.S. Effect of coastal dune restoration on the population of endangered Mongolian racerunner (*Eremias argus*) in the Republic of Korea. *J. Coast. Conserv.* **2021**, *25*, 29. https://doi.org/10.1007/s11852-021-00820-9.
171. Kim, J.K.; Song, J.Y.; Lee, J.H.; Park, D. Physical characteristics and age structure of Mongolian racerunner (*Eremias argus*; Larcertidae; Reptilia). *J. Ecol. Field Biol.* **2010**, *33*, 325–331. https://doi.org/10.5141/JEFB.2010.33.4.325.
172. Kim, B.N.; Kyeong, K.G.; Park, D. Mating behavior of the Mongolian racerunner (*Eremias argus*; Lacertidae, Reptilia). *Anim. Cells Syst.* **2012**, *16*, 4, 337–342. https://doi.org/10.1080/19768354.2012.657242.
173. Song, J.; Koo, K.; Chang, M. Movement and home range of the Mongolian racerunner, *Eremias argus* (Squamata: lacertidae): A preliminary result. *Korean J. Herpetol.* **2010**, *2*, 17–21.
174. Ma, L.; Guo, K.; Su, S.; Lin, L.H.; Xia, Y.; Ji, X. Age-related reproduction of female Mongolian racerunners (*Eremias argus*; Lacertidae): Evidence of reproductive senescence. *J. Exp. Zool.* **2019**, *331*, 290–298. https://doi.org/10.1002/jez.2264.
175. Zhang, Z.R.; Zhu, Q.; Chen, J.D.; Khattak, R.H.; Li, Z.; Teng, L.; Liu, Z. Insights into the composition of gut microbiota in response to environmental temperature: The case of the Mongolia racerunner (*Eremias argus*). *Glob. Ecol. Conserv.* **2022**, *36*, e02125. https://doi.org/10.1016/j.gecco.2022.e02125.
176. Huang, X.B.; Wu, H.H.; Tu, X.B.; Zhang, Z.; Su, H.; Shi, Y.; Wang, G.; Cao, G.; Nong, X.; Zhang, Z. Diets structure of a common lizard *Eremias argus* and their effects on grasshoppers: Implications for a potential biological agent. *J. Asia-Pac. Entomol.* **2016**, *19*, 133–138. https://doi.org/10.1016/j.aspen.2015.12.013.
177. *GBIF*: The Global Biodiversity Information Facility (2022) What is GBIF? [Cited 7 Oct 2023.] Available from URL: https://www.gbif.org/what-is-gbif.

Table S7 Coordinates used for ecological niche modeling.

Site number	Lon	Lat	Reference
1	126.89	38.33	[23]
2	125.83	39.07	[23]
3	128.12	38.65	[23]
4	126.35	40.42	[23]
5	117.13	42.2	[25]
6	118.3	32.3	[32]
7	112.6	35.1	[32]
8	113.13	45.3	[77]
9	105.42	45.48	[77]
10	114.18	47.29	[77]
11	114.46	48.06	[77]
12	117.67	46.83	[78]
13	102.83	49.42	[79]
14	105.25	42.48	[79]
15	106.79	42.53	[79]
16	98.82	42.88	[79]
17	97.98	43.11	[79]
18	107.48	43.11	[79]
19	102.02	43.16	[79]
20	109.15	43.33	[79]
21	103.41	43.44	[79]
22	97.91	43.52	[79]
23	100.07	43.58	[79]
24	110.58	43.62	[79]
25	101.39	43.99	[79]
26	99.3	44.63	[79]
27	96.25	44.93	[79]
28	109.98	45.15	[79]
29	92.5	45.48	[79]
30	96.9	45.82	[79]
31	99.26	45.82	[79]
32	108.71	46.11	[79]
33	94.55	46.13	[79]
34	91.58	46.17	[79]
35	96.76	46.68	[79]
36	103.64	47.4	[79]
37	92.42	47.74	[79]
38	126.38	36.41	[171]
39	114.47	36.6	[173]
40	105.94	38.57	[175]
41	109.31	42.13	[176]
42	108.83	33.99	This study
43	114.37	34.81	This study
44	104.17	35.97	This study

45	100.35	36.28	This study
46	110.41	36.72	This study
47	121.70	36.84	This study
48	103.81	36.88	This study
49	106.17	37.45	This study
50	109.78	38.17	This study
51	107.70	38.18	This study
52	107.27	39.10	This study
53	122.17	39.56	This study
54	110.85	40.05	This study
55	115.82	40.38	[177]
56	105.30	47.88	[177]
57	117.09	35.79	[177]
58	108.68	39.27	[177]
59	111.81	40.58	[177]
60	108.50	41.34	This study
61	110.84	41.44	This study
62	113.48	41.55	This study
63	110.33	42.48	This study
64	112.58	42.86	This study
65	120.34	42.89	This study
66	107.46	51.73	This study
67	118.03	43.24	This study
68	122.24	43.24	This study
69	115.69	43.33	This study
70	113.13	43.76	This study
71	114.72	43.95	This study
72	115.92	44.20	This study
73	116.12	44.92	This study
74	126.22	45.58	This study
75	118.35	47.63	This study
76	117.38	48.29	This study
77	106.18	50.24	This study

References

23. Szyndlar, Z. Distributional records for turtles and lizards from North Korea. *Herpetol. Rev.* **1991**, *22*, 27.
25. Zeng, Z.G.; Bi, J.H.; Li, S.R.; Chen, S.Y.; Du, W.G. Habitat alteration influences a desert steppe lizard community: implications of species specific preferences and performance. *Herpetol. Monogr.* **2016**, *30*, 34–48. https://doi.org/10.1655/HERPMONOGRAPHS-D-14-00008.1.
32. Zhao, Q.; Liu, H.X.; Luo, L.G.; Ji, X. Comparative population genetics and phylogeography of two lacertid lizards (*Eremias argus* and *E. brenchleyi*) from China. *Mol. Phylogenet. Evol.* **2011**, *58*, 478–491. https://doi.org/10.1016/j.ympev.2010.12.017.
77. Alberto, S.V.; Marta, C.; Mario, G.P.; Judit, V.; José, G.A. Amphibians and reptiles from Zoltan Kaszab's expeditions to Mongolia held at the Hungarian Natural History Museum. *Acta Zool. Acad. Sci. H.* **2019**, *65*, 143–166. https://doi.org/10.17109/AZH.65.2.143.2019.
78. Ananjeva, N.B.; Munkhbayar, K.; Orlov, N.L.; Orlova, V.F.; Semenov, D.V.; Terbish, K. *Amphibians and reptiles of Mongolia. Reptiles of Mongolia*; KMK Press: Moscow, Russia, 1997. (in Russian with English summary)

79. Buehler, M.D.; Zoljargal, P.; Purvee, E. Batsaikhan, N.; Ananjeva, N.B.; Orlov, N.L.; Panpenfuss, T.J.; Roldán-Piña, D. The results of four recent joint expedition to the Gobi Desert: lacertids and agamids. *Russ. J. Herpetol.* **2021**, *28*, 15–32. https://doi.org/10.30906/1026-2296-2021-28-1-15-32.

171. Kim, J.K.; Song, J.Y.; Lee, J.H.; Park, D. Physical characteristics and age structure of Mongolian racerunner (*Eremias argus*; Larcertidae; Reptilia). *J. Ecol. Field Biol.* **2010**, *33*, 325–331. https://doi.org/10.5141/JEFB.2010.33.4.325.

173. Song, J.; Koo, K.; Chang, M. Movement and home range of the Mongolian racerunner, *Eremias argus* (Squamata: lacertidae): A preliminary result. *Korean J. Herpetol.* **2010**, *2*, 17–21.

174. Ma, L.; Guo, K.; Su, S.; Lin, L.H.; Xia, Y.; Ji, X. Age-related reproduction of female Mongolian racerunners (*Eremias argus*; Lacertidae): Evidence of reproductive senescence. *J. Exp. Zool.* **2019**, *331*, 290–298. https://doi.org/10.1002/jez.2264.

175. Zhang, Z.R.; Zhu, Q.; Chen, J.D.; Khattak, R.H.; Li, Z.; Teng, L.; Liu, Z. Insights into the composition of gut microbiota in response to environmental temperature: The case of the Mongolia racerunner (*Eremias argus*). *Glob. Ecol. Conserv.* **2022**, *36*, e02125. https://doi.org/10.1016/j.gecco.2022.e02125.

176. Huang, X.B.; Wu, H.H.; Tu, X.B.; Zhang, Z.; Su, H.; Shi, Y.; Wang, G.; Cao, G.; Nong, X.; Zhang, Z. Diets structure of a common lizard *Eremias argus* and their effects on grasshoppers: Implications for a potential biological agent. *J. Asia-Pac. Entomol.* **2016**, *19*, 133–138. https://doi.org/10.1016/j.aspen.2015.12.013.

177. *GBIF*: The Global Biodiversity Information Facility (2022) What is GBIF? [Cited 7 Oct 2023.] Available from URL: https://www.gbif.org/what-is-gbif.

Table S8 Hierarchical analysis of AMOVA for *Eremias argus*.

Source of variation	*d.f.*	Sum of squares	Variance components	Percentage of variation	*Fct*/*Fsc*/*Fst*
Among groups	6	2202.005	5.24249 Va	34.98	0.34983
Among populations Within groups	100	3195.820	4.96200 Vb	33.11	0.50928
Within populations	507	2419.261	4.78115 Vc	31.90	0.68095
Total	613	7817.086	14.98564		

Table S9 Descriptive statistics by subclade/clades of *Eremias argus*.

Clade/ Subclade	*N*	Tajima's *D*	MNPD	ND	HD	Fu's *Fs*	OMMD	*SSD*	*Rg*	R_2
Ia	254	–1.466	27.930	0.027	0.982	–32.342***	Multimodal	0.003758	0.00204475	0.16350***
Ib	72	–2.094*	3.764	0.004	0.396	–1.035	Multimodal	0.036891	0.29763057	0.16098***
Ic	26	–0.703	17.822	0.017	0.858	0.596	Multimodal	0.036288 **	0.05775148**	0.16164***
Id	55	–0.895	26.656	0.026	0.966	–4.929**	Multimodal	0.005882	0.00729676 **	0.16133***
Ie	36	0.337	28.400	0.027	0.868	2.613	Multimodal	0.031325***	0.0469614***	0.16236***
If	105	0.136	26.398	0.025	0.925	6.700***	Multimodal	0.018891 *	0.02207033 ***	0.16195***
IIb	64	–0.663	23.546	0.023	0.954	–5.578**	Multimodal	0.009910	0.01964405 ***	0.16116***
IIa	2	—	—	—	—	—	—	—	—	—
Clade I	548	–1.609	25.992	0.025	0.981	–32.532***	Multimodal	0.004680	0.00164071	0.16152***
Clade II	66	–0.735	23.766	0.023	0.956	–5.727**	Multimodal	0.009265	0.01651767 ***	0.16105***

N, number of haplotypes; MNBD, mean number of base-pair differences; ND, nucleotide diversity; HD, haplotype diversity; OMMD, observed modality of mismatch distribution; *SSD*, sum of square deviation between the observed and simulated mismatch distributions; *Rg*, raggedness index; R_2, R_2 statistics.*$P < 0.05$; **$P < 0.01$; ***$P < 0.001$.
